# Supplementary material for: Discovery of Ureido-Based Apcin Analogues as Cdc20-specific Inhibitors against Cancer
Source: Pharmaceuticals (Basel). 2023 Feb 15;16(2):304. doi: 10.3390/ph16020304 (PMC9964651; doi:10.3390/ph16020304)
Supplement: Supplementary file 1 [file pharmaceuticals-16-00304-s001.zip › pharmaceuticals-2190358-supplementary.pdf]

Supplementary Information

Discovery of ureido-like Apcin analogs as Cdc20-specific inhibitors against cancer

Yiqin He [1,2,3], Xiangyang Le [1,2,3], Gaoyun Hu [1,2,3], Qianbin Li [1,2,3], Zhuo Chen [1,2,3]*

[1] Department of Medicinal Chemistry, Xiangya School of Pharmaceutical Sciences, Central South University, Changsha 410013, Hunan, China

[2] Hunan Key Laboratory of Organ Fibrosis; Changsha 410013, Hunan, China

[3] Hunan Key Laboratory of Diagnostic and Therapeutic Drug Research for Chronic Diseases; Changsha 410013, Hunan, China

* Correspondence: (Email: cz_job@csu.edu.cn) +86-826500370.

Contents

NMR, HRMS and HMBC spectra of compounds page 2-48

Compound 1

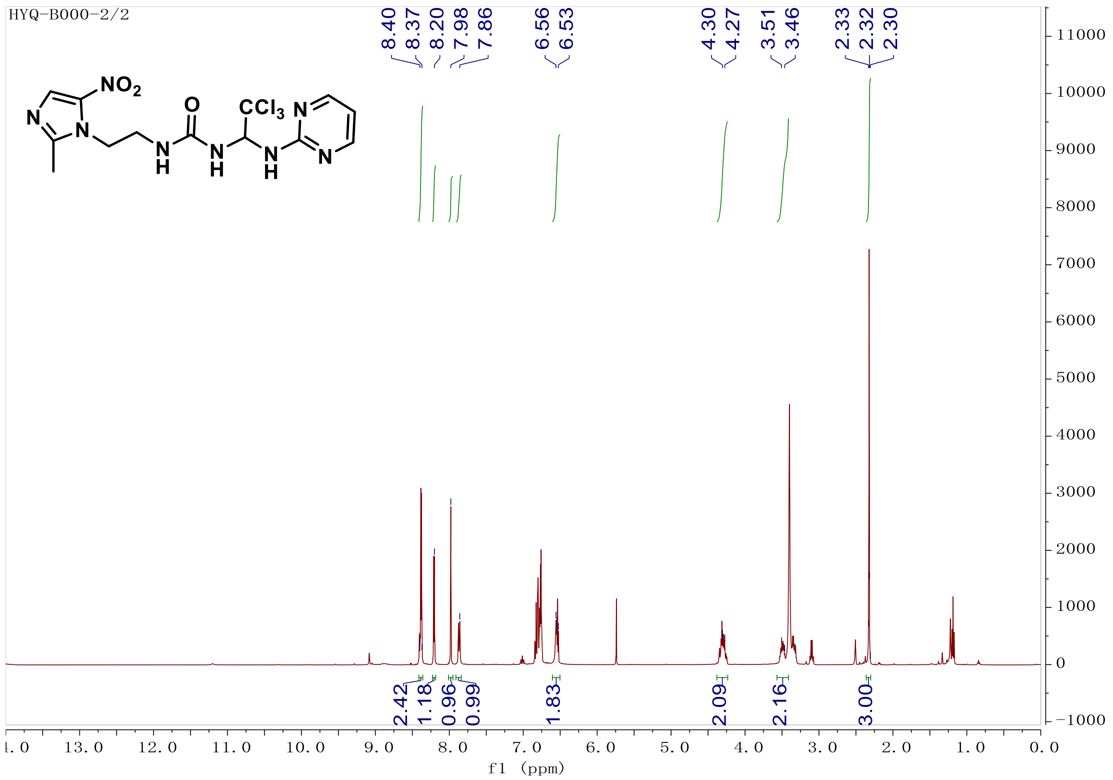
HYQ-B000-2/2
NO2
N
N
O
CCl3
N
N
H
H
H
N
N
8.40
8.37
8.20
7.98
7.86
6.56
6.53
4.30
4.27
3.51
3.46
2.33
2.32
2.30
2.42
1.18
0.96
0.99
1.83
2.09
2.16
3.00
f1 (ppm)

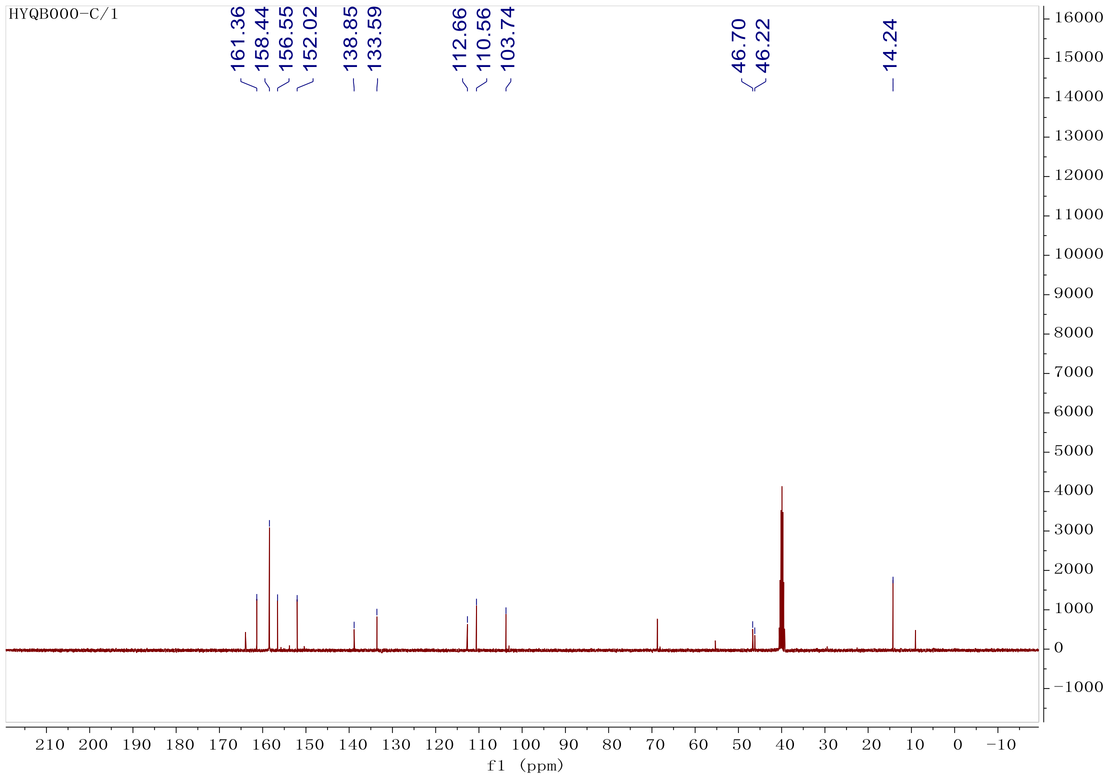
HYQB000-C/1
161.36
158.44
156.55
152.02
138.85
133.59
112.66
110.56
103.74
46.70
46.22
14.24
f1 (ppm)

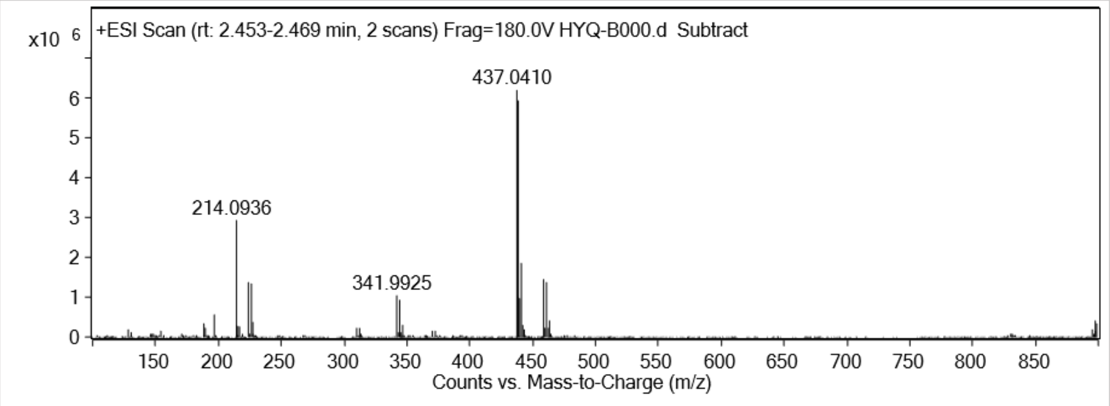

Peak List

m/z	z	Abund
214.0936	1	2913896.75
223.9545	1	1386203.63
225.9516	1	1322869.38
341.9925	1	1032641.06
437.041	1	6189377.5
438.0437	1	985039.63
439.0382	1	5931451.5
441.0356	1	1867002
459.0229	1	1439060.38
461.0201	1	1388403.75

Compound 2

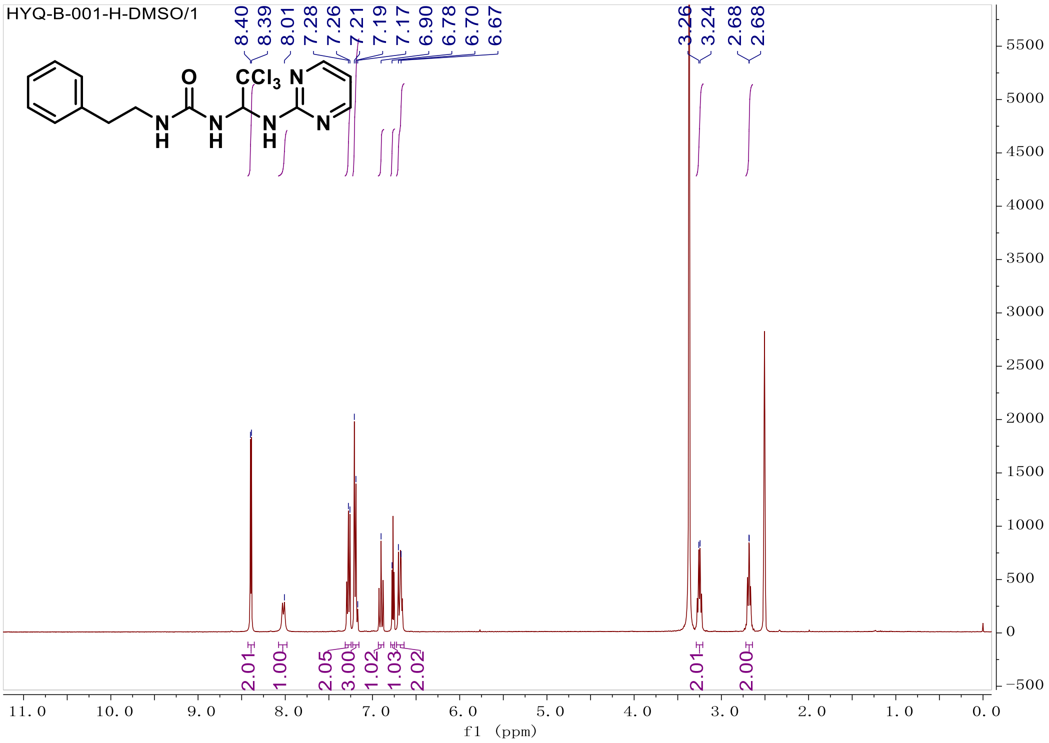

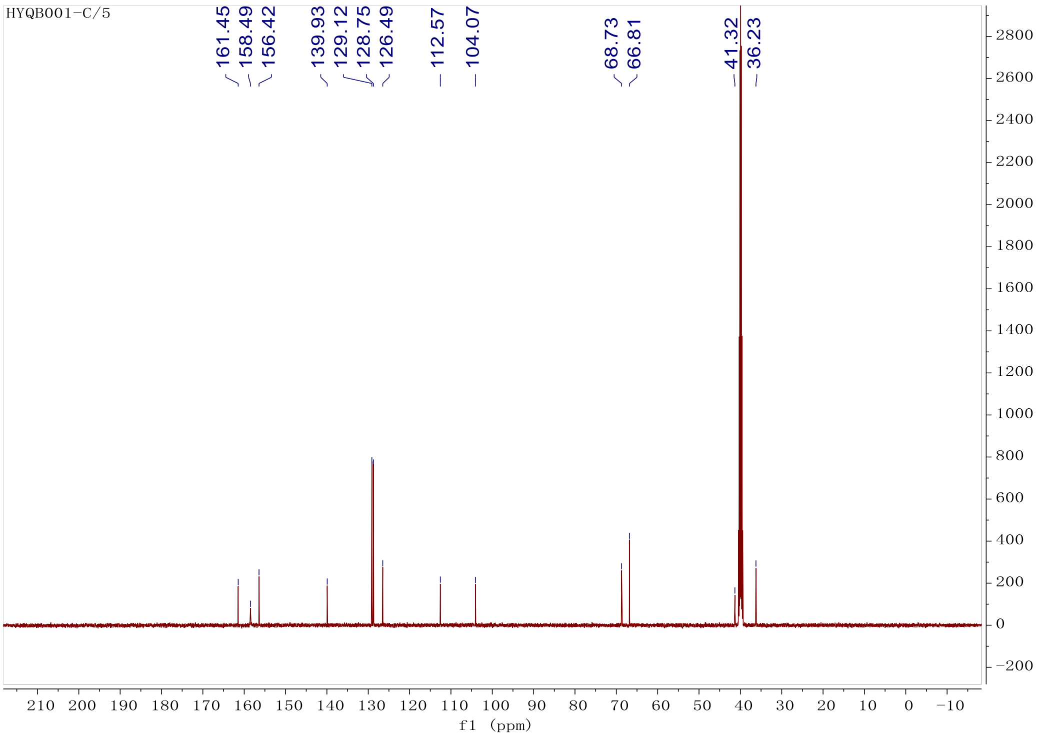

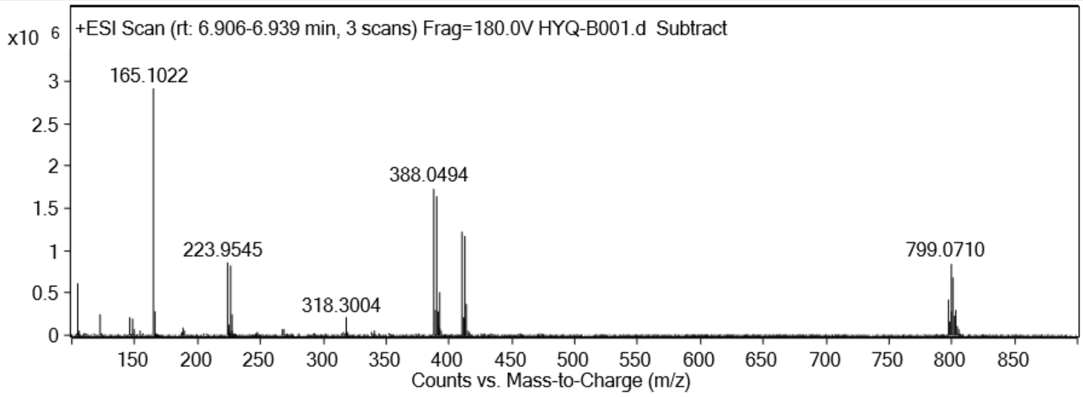

Peak List

m/z	z	Abund
105.0699	1	616980.81
165.1022	1	2918582.25
223.9545	1	858449.63
225.9515	1	813062
388.0494	1	1737970.25
390.0466	1	1640183.38
410.0313	1	1227437.25
412.0286	1	1173123.88
799.071	1	847249
801.0687	1	684362

Compound 3

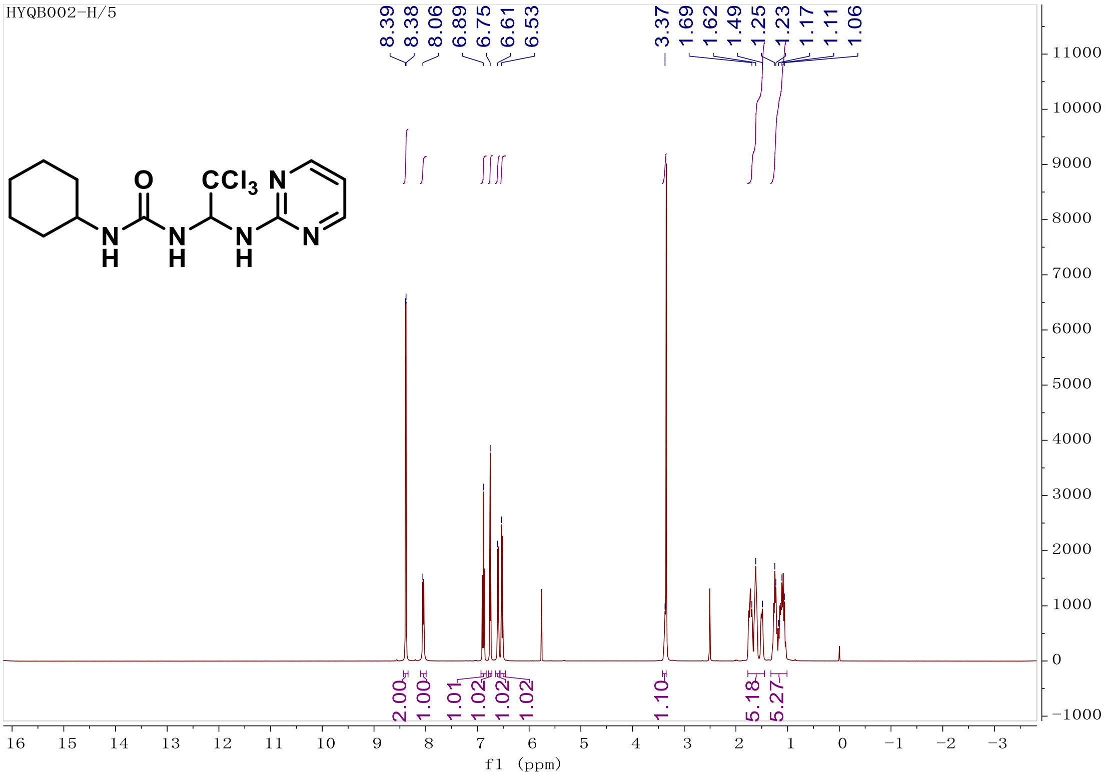
HYQB002-H/5
8.39
8.38
8.06
6.89
6.75
6.61
6.53
3.37
1.69
1.62
1.49
1.25
1.23
1.17
1.11
1.06
2.00
1.00
1.01
1.02
1.02
1.02
1.10
5.18
5.27
f1 (ppm)

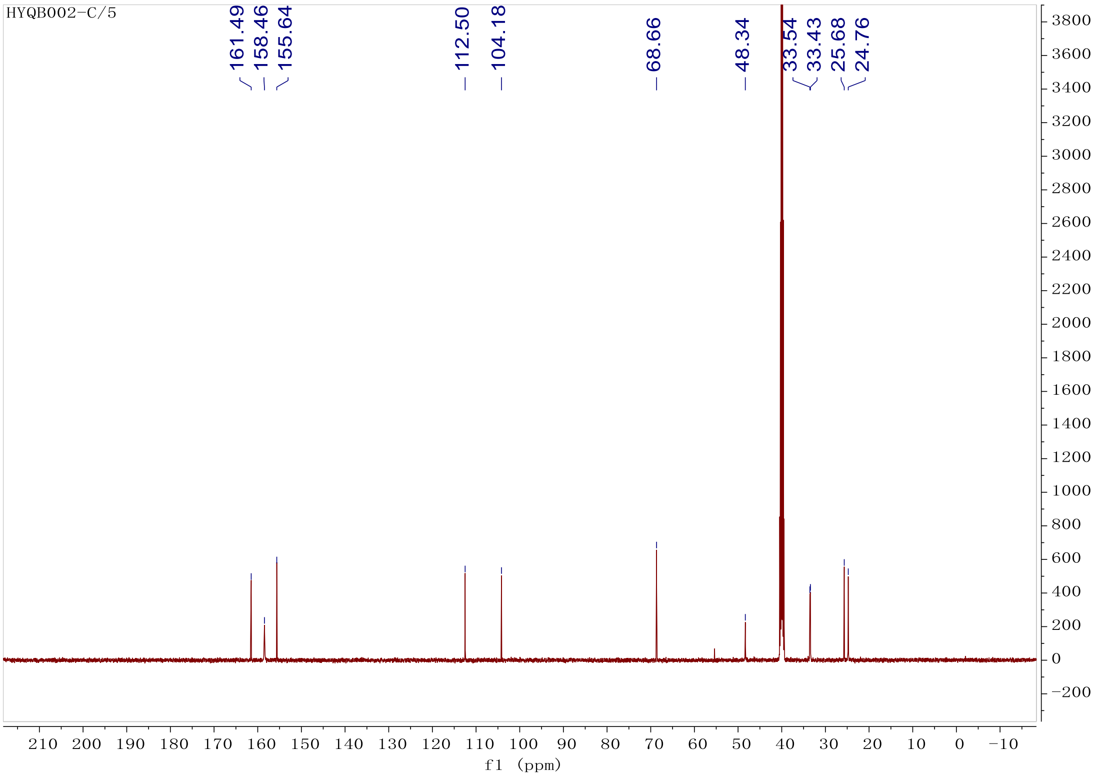
HYQB002-C/5
161.49
158.46
155.64
112.50
104.18
68.66
48.34
33.54
33.43
25.68
24.76
f1 (ppm)

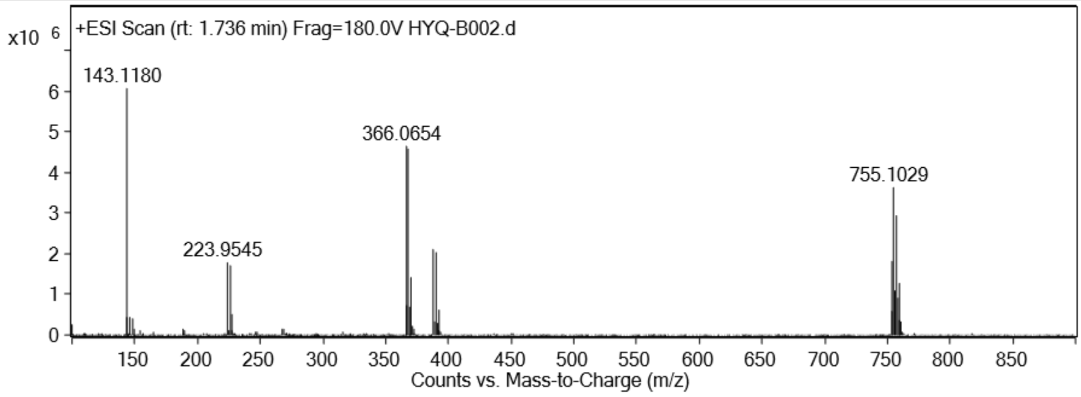

Peak List

m/z	z	Abund
143.118	1	6070176.5
223.9545	1	1794369.88
225.9516	1	1695645.38
366.0654	1	4636283
368.0626	1	4570204
388.0474	1	2100329
390.0445	1	2036202.5
753.1056	1	1813054.63
755.1029	1	3644184.75
757.1003	1	2950037

Compound 4

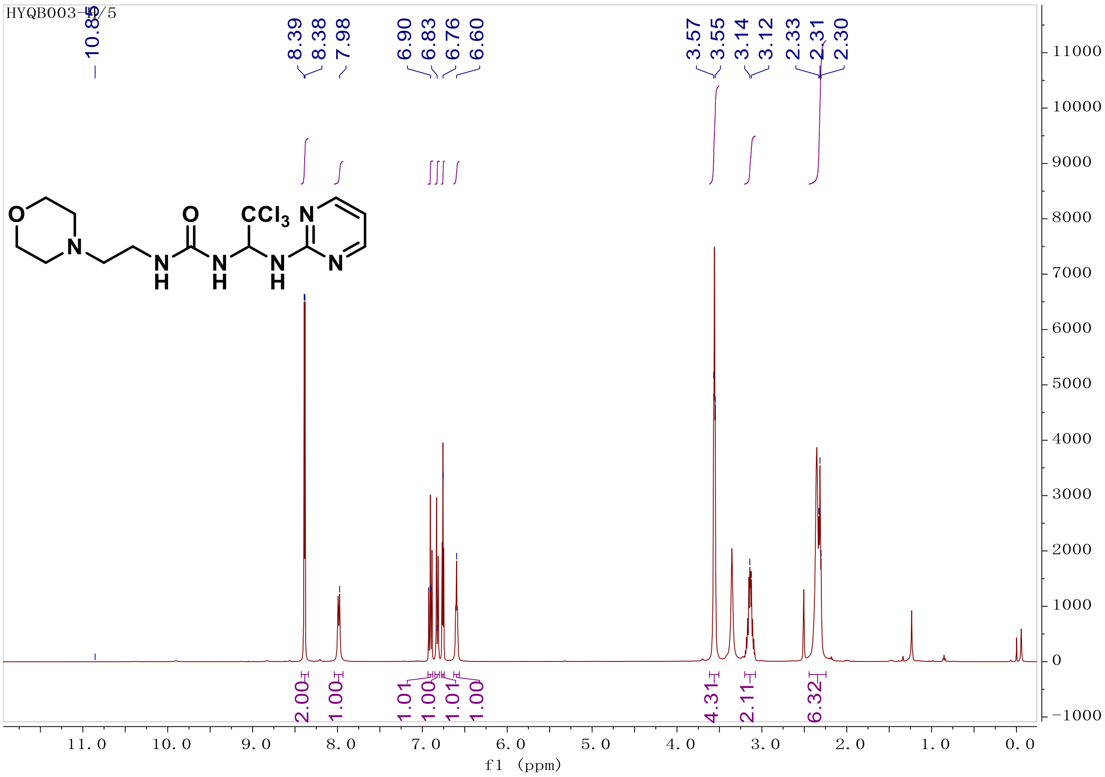

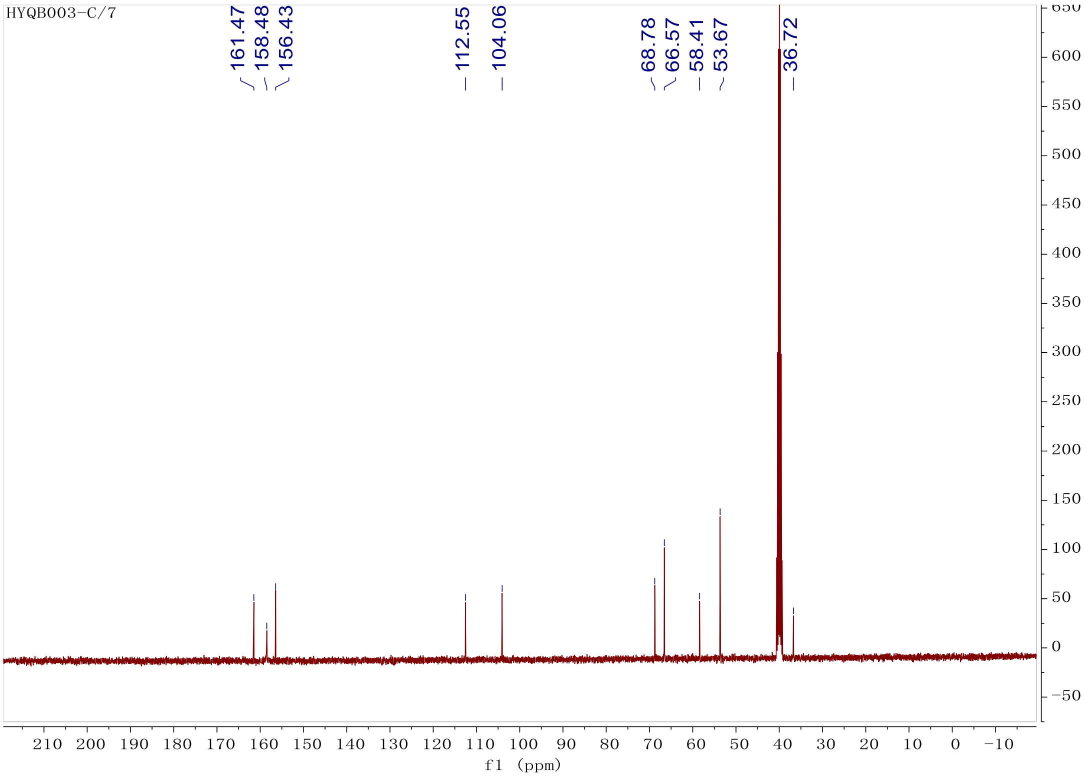

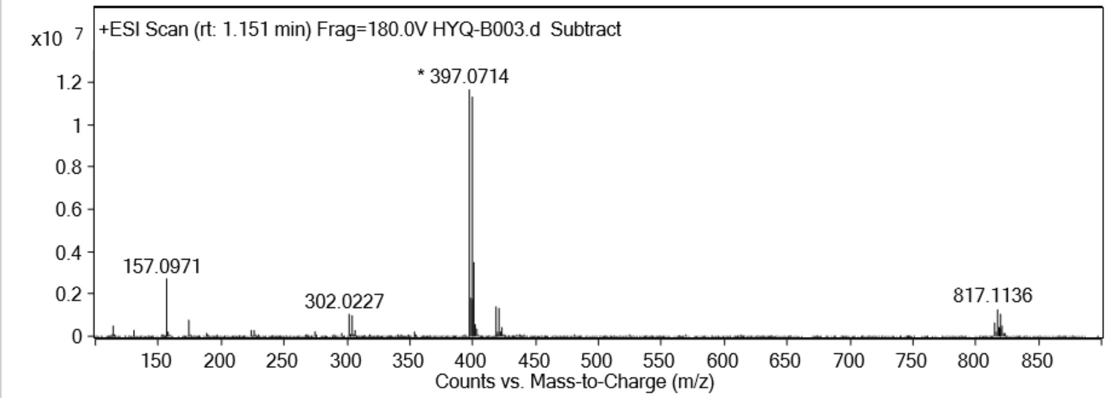

Peak List

m/z	z	Abund
157.0971	1	2711610.25
397.0714	1	11661401
398.0737	1	1811432.63
399.0685	1	11339091
400.0708	1	1740182
401.0654	1	3462262.5
419.0528	1	1401176.25
421.05	1	1321380.38
817.1136	1	1293721.5
819.1111	1	1064166.38

Compound 5

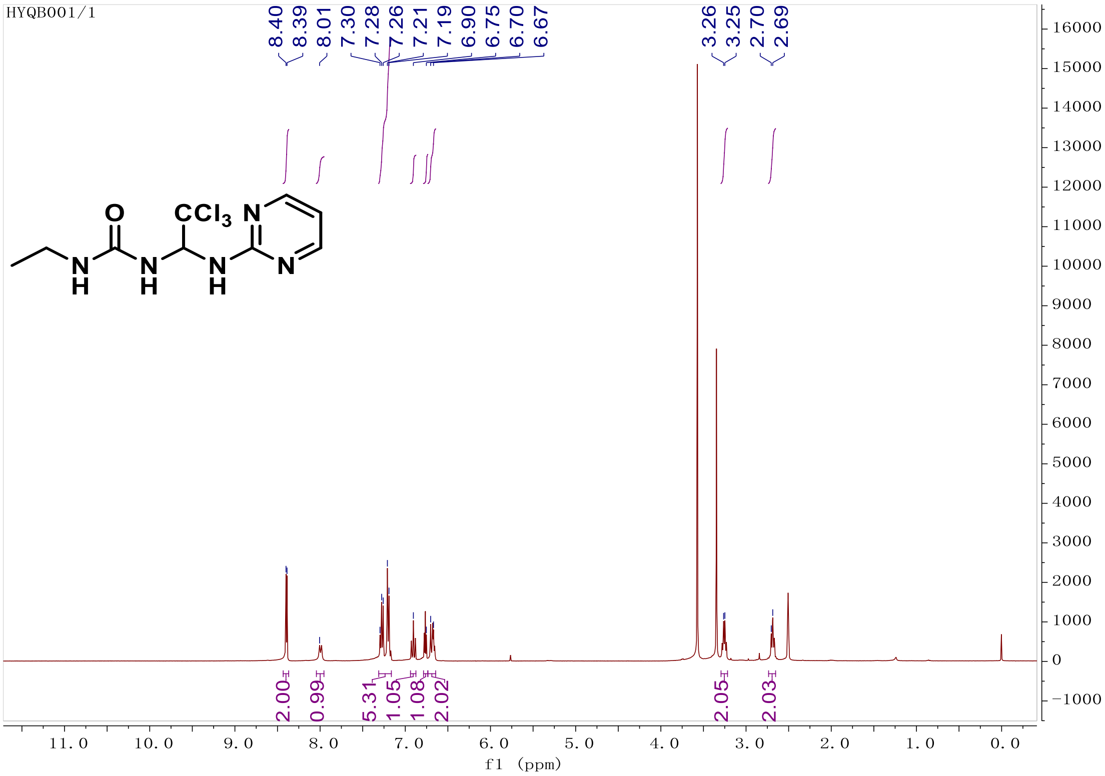
HYQB001/1
8.40
8.39
8.01
7.30
7.28
7.26
7.21
7.19
6.90
6.75
6.70
6.67
3.26
3.25
2.70
2.69
CCl3
O
N
N
N
N
H
H
H
2.00
0.99
5.31
1.05
1.08
2.02
2.05
2.03
f1 (ppm)

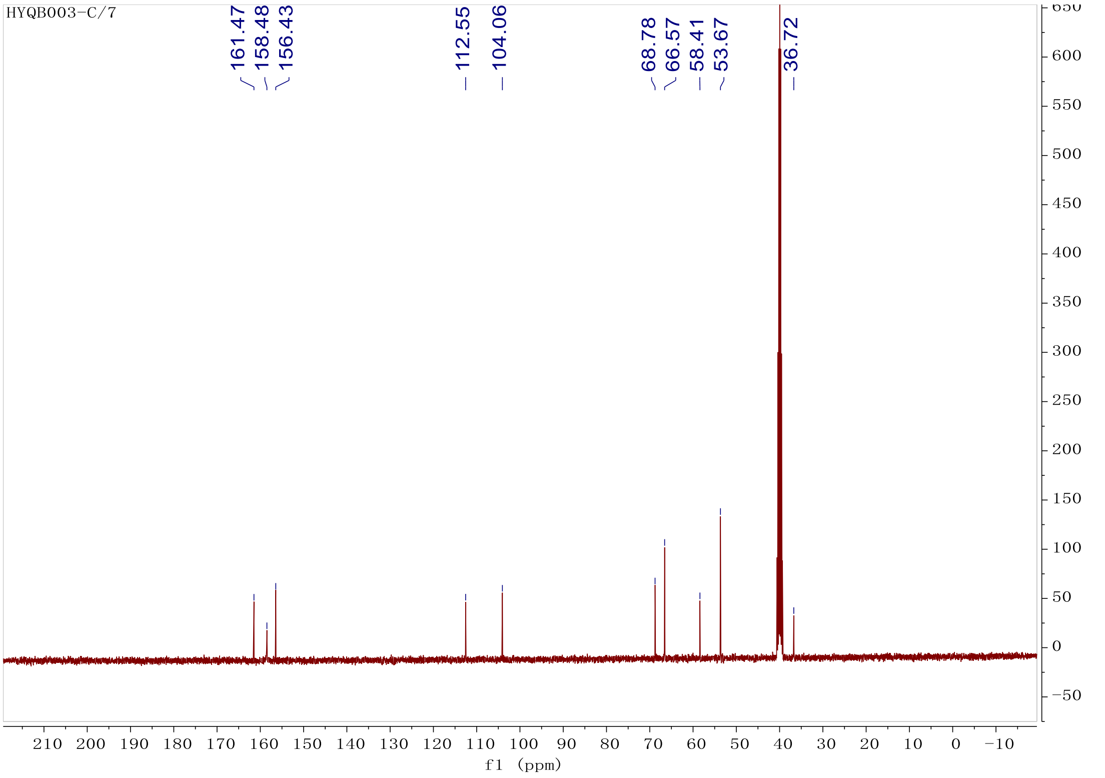
HYQB003-C/7
161.47
158.48
156.43
112.55
104.06
68.78
66.57
58.41
53.67
36.72
f1 (ppm)

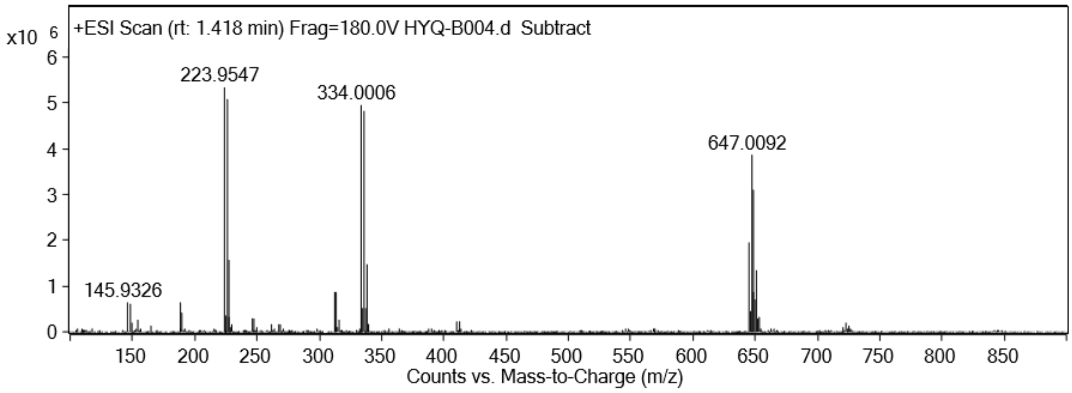

Peak List

m/z	z	Abund
223.9547	1	5329879
225.9518	1	5086102.5
227.9488	1	1556247.63
334.0006	1	4929472
335.9977	1	4800584.5
337.9949	1	1480485.63
645.0121	1	1946913
647.0092	1	3872751
649.0065	1	3084400.5
651.0039	1	1337346.38

Compound 6

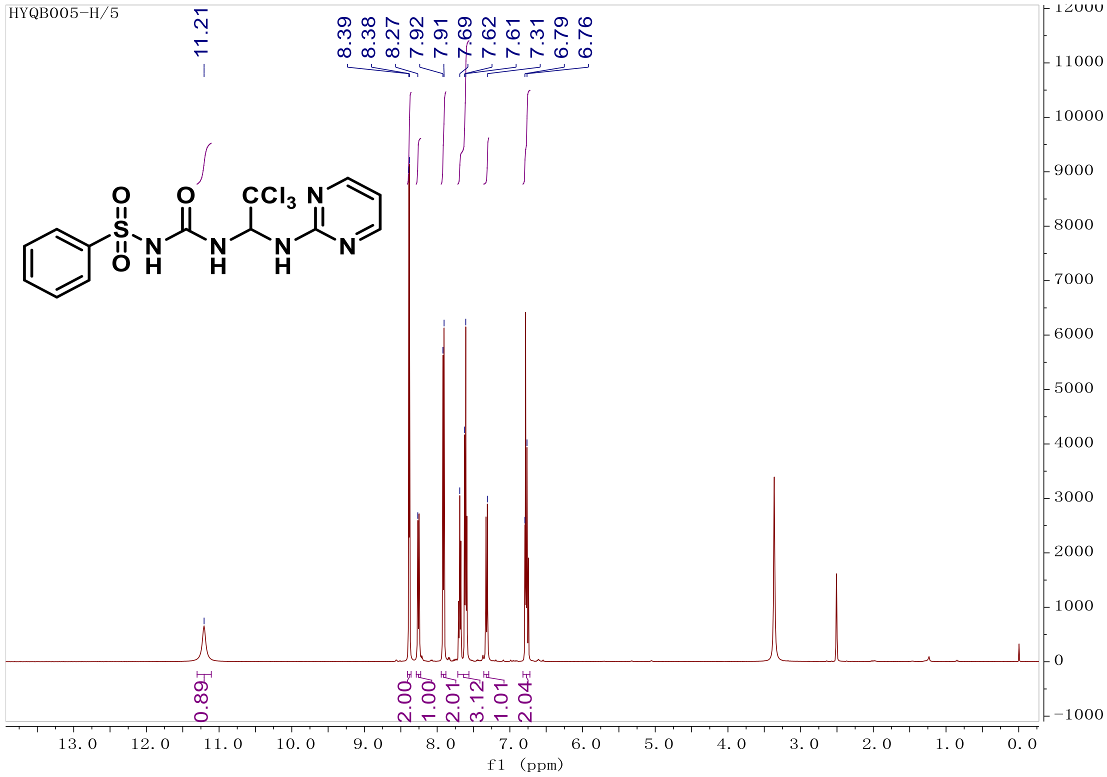

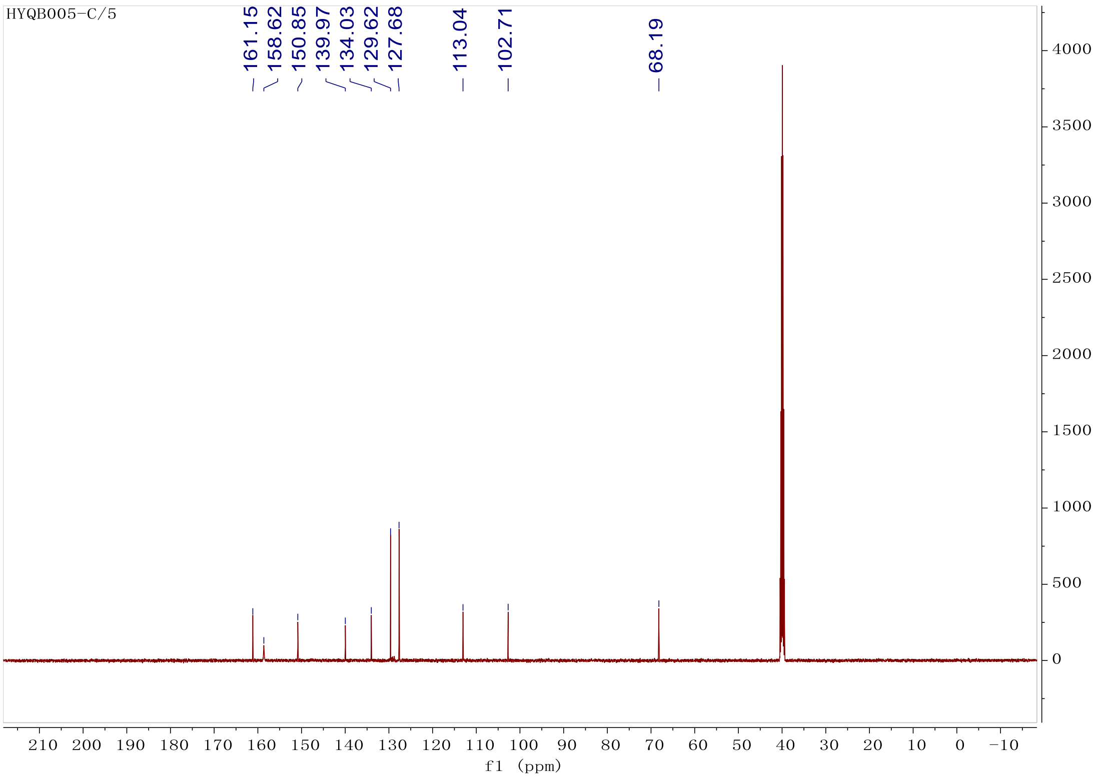

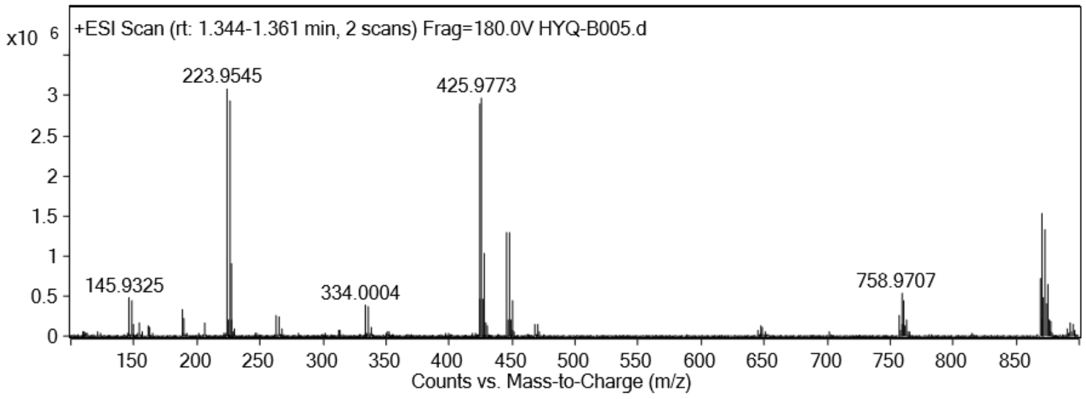

Peak List

m/z	z	Abund
223.9545	1	3085585.5
225.9516	1	2938656
227.9488	1	912903.38
423.9802	1	2895090.75
425.9773	1	2975801.75
427.9746	1	1032621.13
445.962	1	1285488.5
447.9593	1	1298665.75
870.9325	1	1533252.25
872.9298	1	1330868

Compound 7

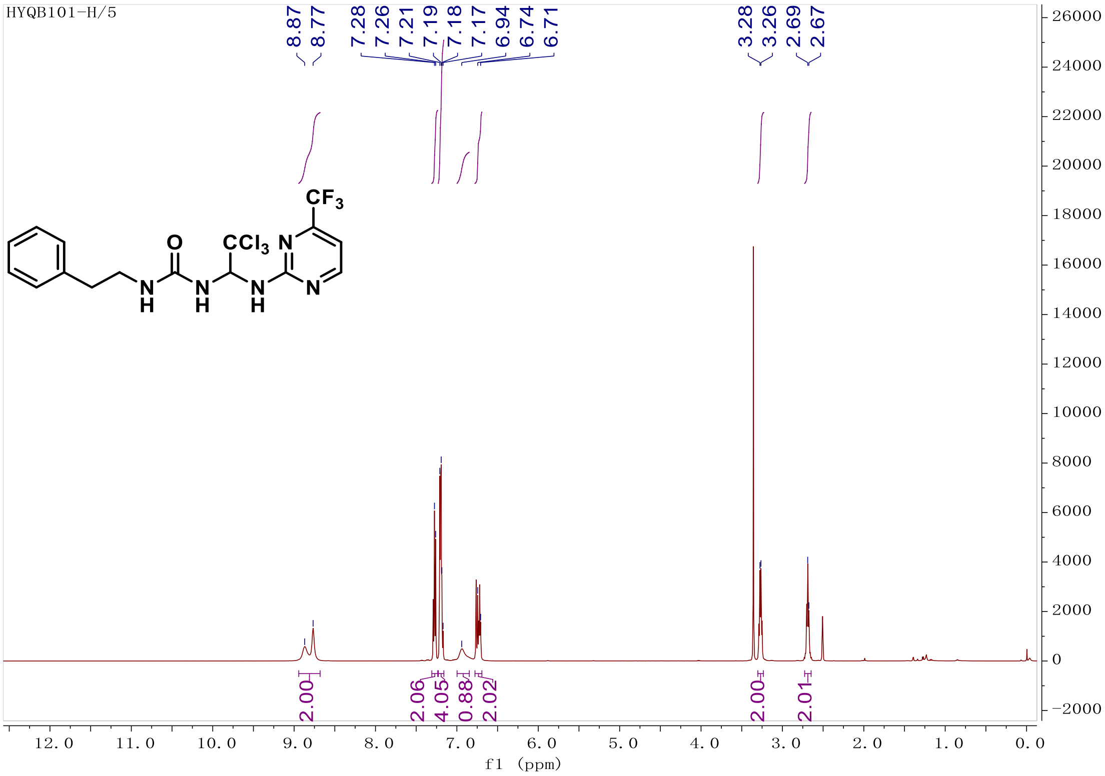
HYQB101-H/5
8.87
8.77
7.28
7.26
7.21
7.19
7.18
7.17
6.94
6.74
6.71
3.28
3.26
2.69
2.67
2.00
2.06
4.05
0.88
2.02
2.00
2.01
f1 (ppm)

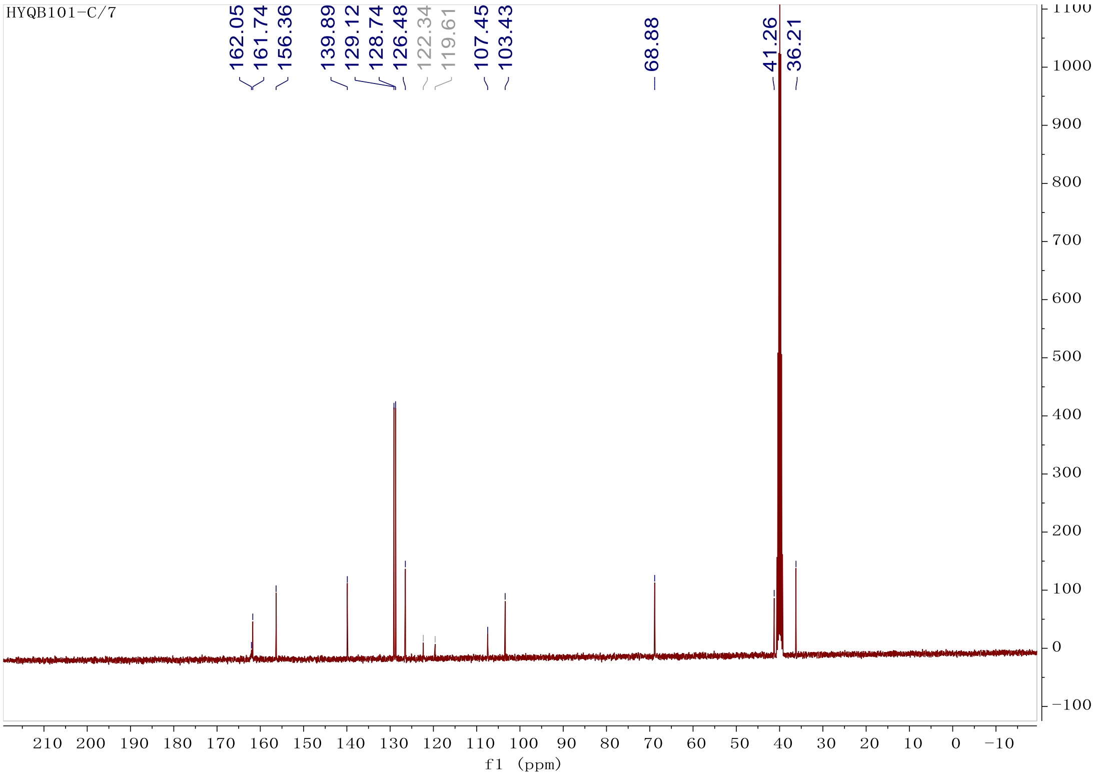
HYQB101-C/7
162.05
161.74
156.36
139.89
129.12
128.74
126.48
122.34
119.61
107.45
103.43
68.88
41.26
36.21
f1 (ppm)

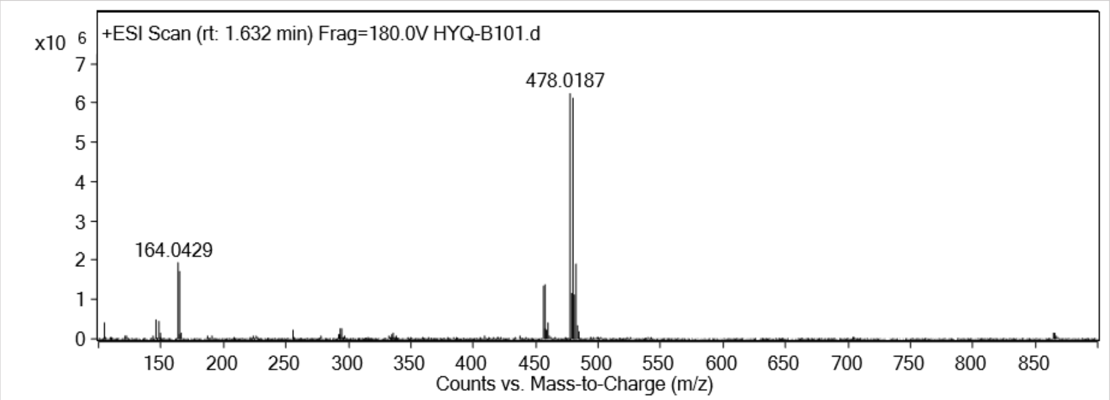

Peak List

m/z	z	Abund
145.9326	1	475618
164.0429	1	1944462.63
165.1022	1	1705610.25
456.0368	1	1357340.5
458.0338	1	1371877.75
478.0187	1	6249155
479.0217	1	1146384.5
480.0159	1	6138824
481.019	1	1127489.5
482.0133	1	1911719.75

Compound 8

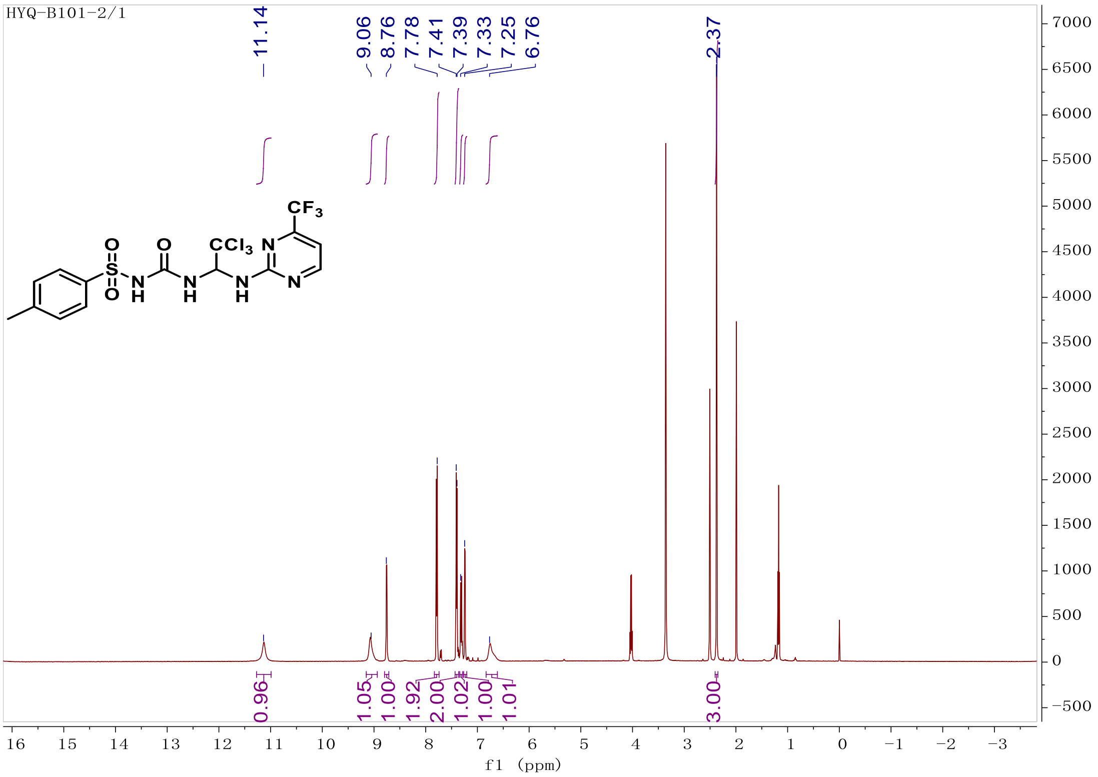

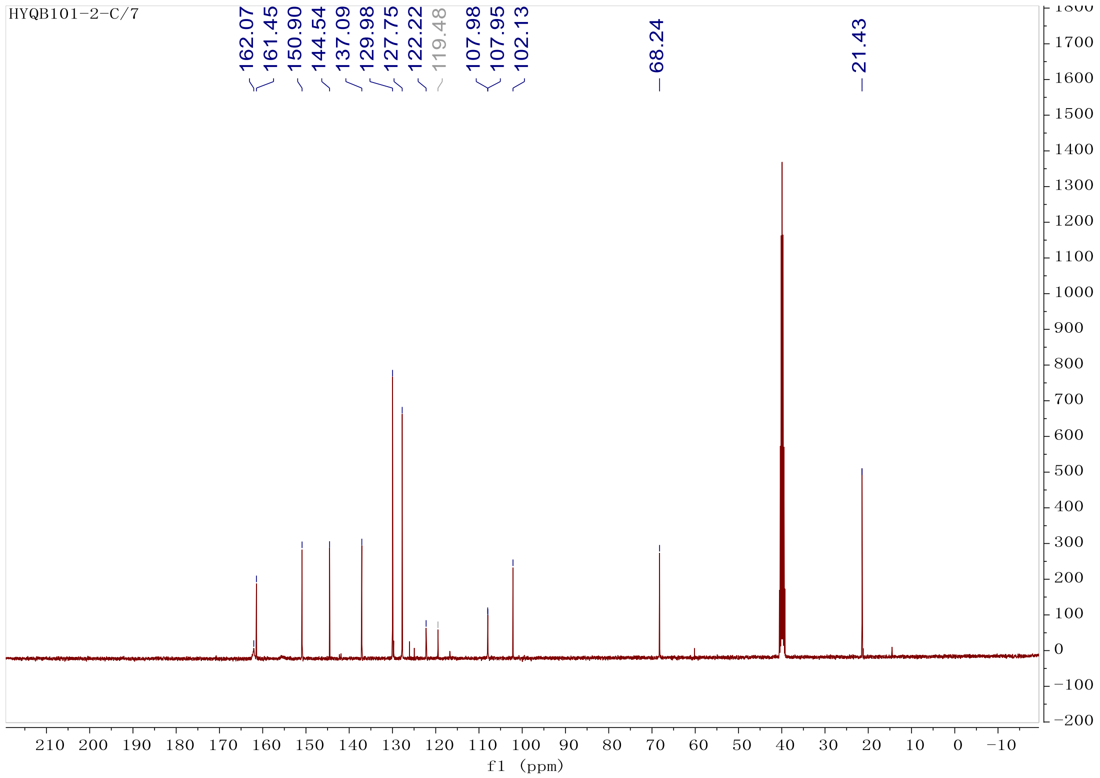

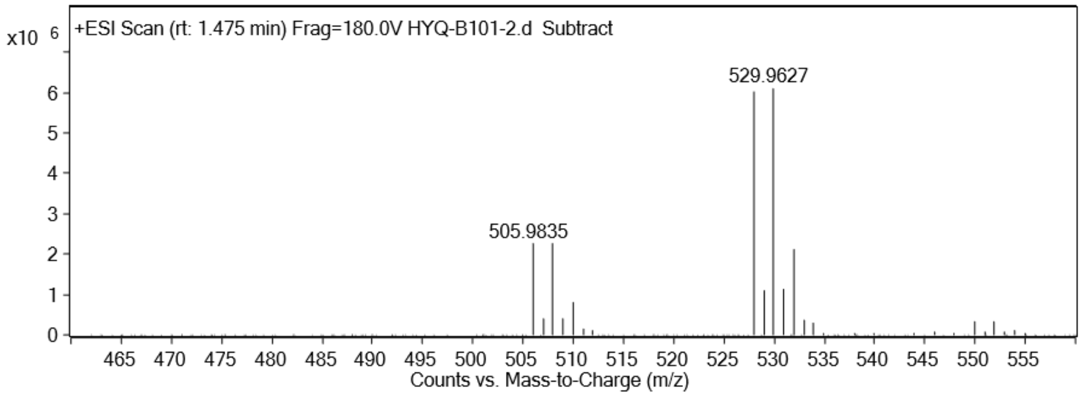

Peak List

m/z	z	Abund
164.0431	1	1858096.13
215.0487	1	973910.81
291.9423	1	957323
505.9835	1	2280364.5
507.9807	1	2272734.5
527.9656	1	6023426
528.9683	1	1102543.5
529.9627	1	6098051
530.9655	1	1123156.25
531.96	1	2106753.25

Compound 9

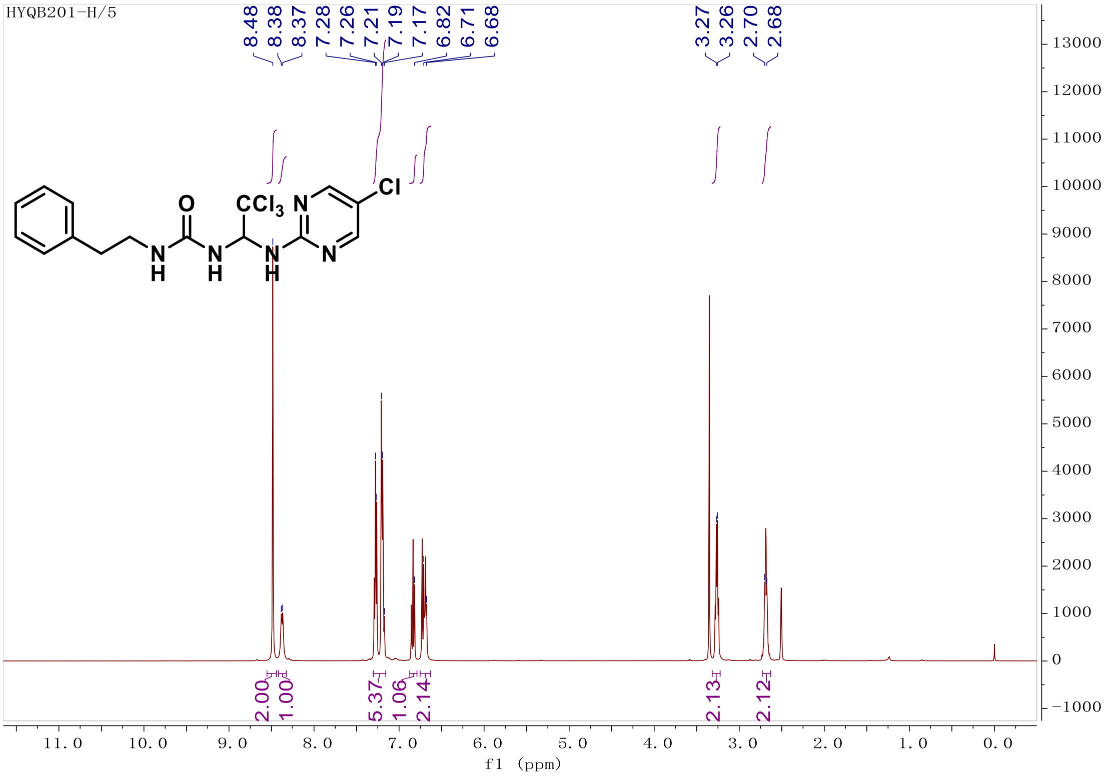
HYQB201-H/5
8.48
8.38
8.37
7.28
7.26
7.21
7.19
7.17
6.82
6.71
6.68
3.27
3.26
2.70
2.68
2.00
1.00
5.37
1.06
2.14
2.13
2.12
CCl3
Cl
O
N
H
f1 (ppm)

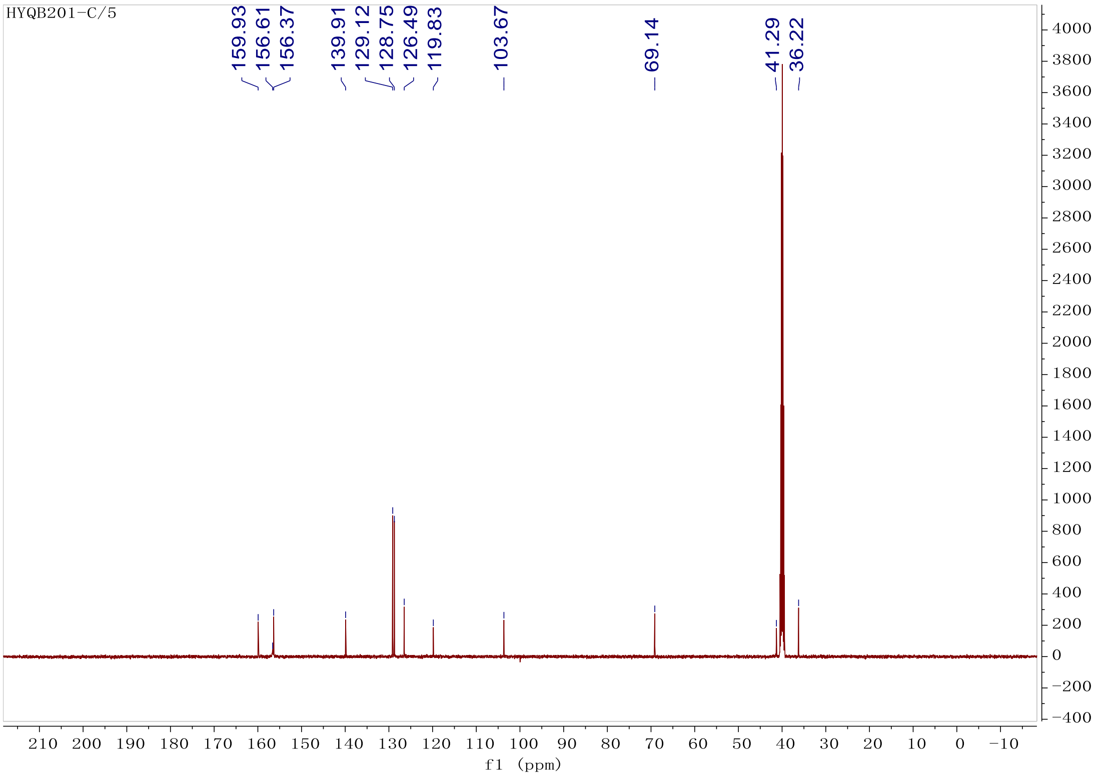
HYQB201-C/5
159.93
156.61
156.37
139.91
129.12
128.75
126.49
119.83
103.67
69.14
41.29
36.22
f1 (ppm)

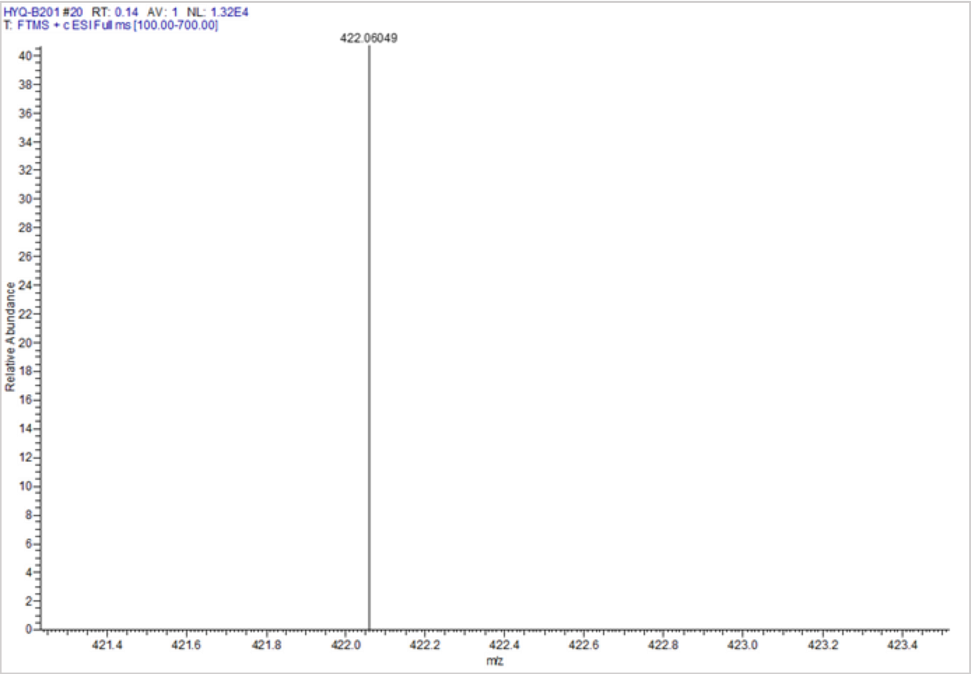
HYQ-B201 #20 RT: 0.14 AV: 1 NL: 1.32E4
T: FTMS + c ESI Full ms [100.00-700.00]
422.06049
Relative Abundance
m/z

Compound 10

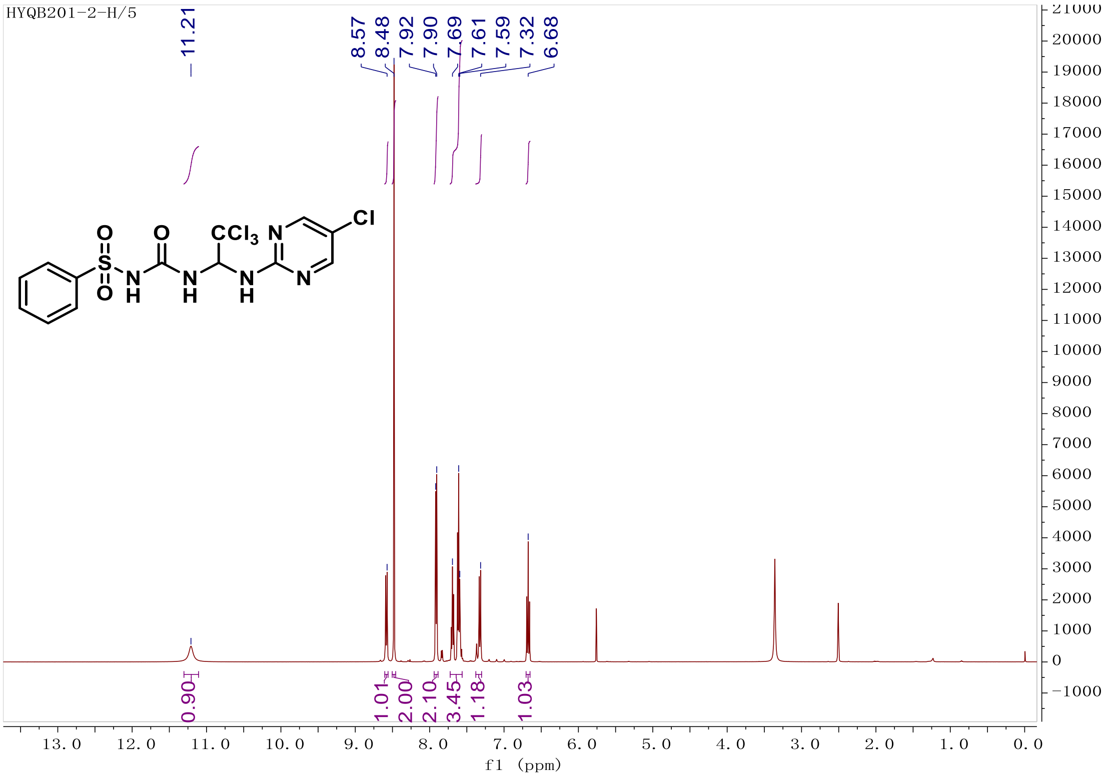
HYQB201-2-H/5
11.21
8.57
8.48
7.92
7.90
7.69
7.61
7.59
7.32
6.68
0.90
1.01
2.00
2.10
3.45
1.18
1.03
CCl3
Cl
f1 (ppm)

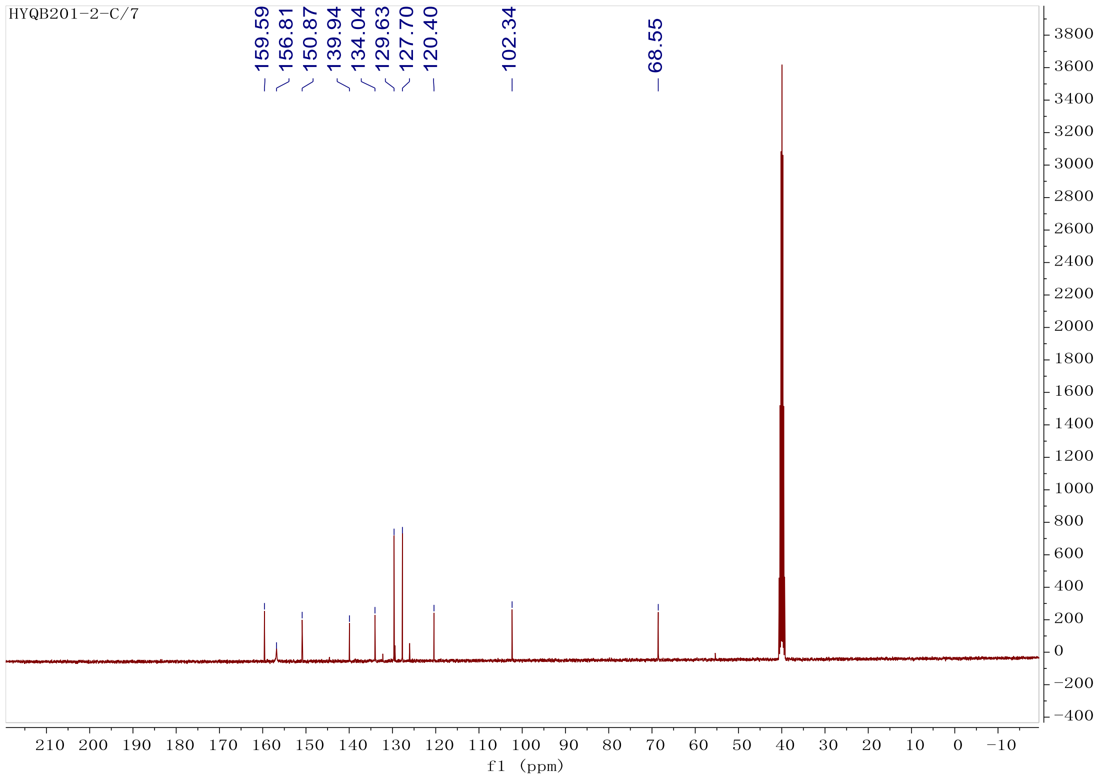

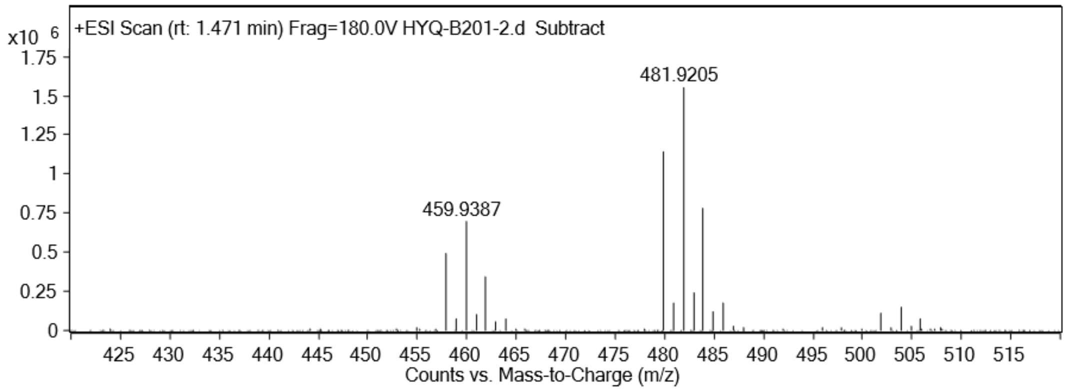

Peak List

m/z	z	Abund
130.0166	1	762194.75
257.9161	1	627702.06
259.913	1	884764
457.9419	1	492440.25
459.9387	1	694760.75
479.9235	1	1146159.88
481.9205	1	1553397.88
483.9179	1	782197
938.8556	1	455398.25
940.8529	1	548085.44

Compound 11

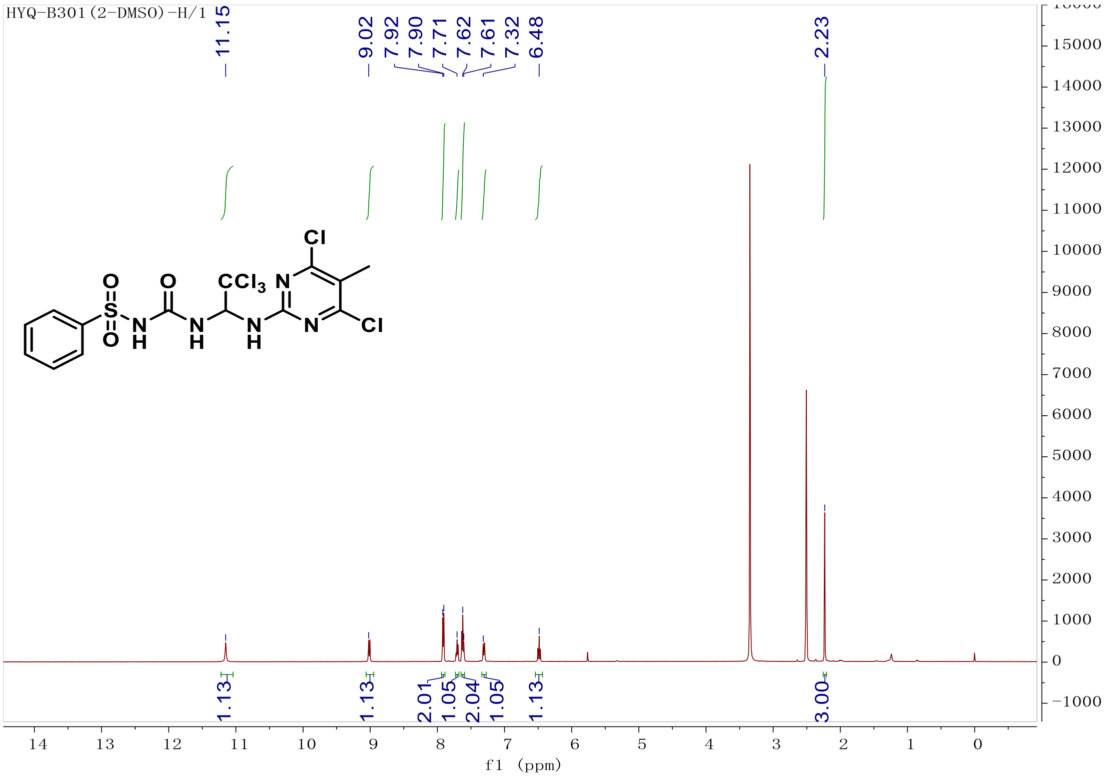
HYQ-B301(2-DMSO)-H/1
11.15
9.02
7.92
7.90
7.71
7.62
7.61
7.32
6.48
2.23
1.13
1.13
2.01
1.05
2.04
1.05
1.13
3.00
CCl3
Cl
Cl
O
O
O
S
N
H
N
H
N
H
N
N
f1 (ppm)

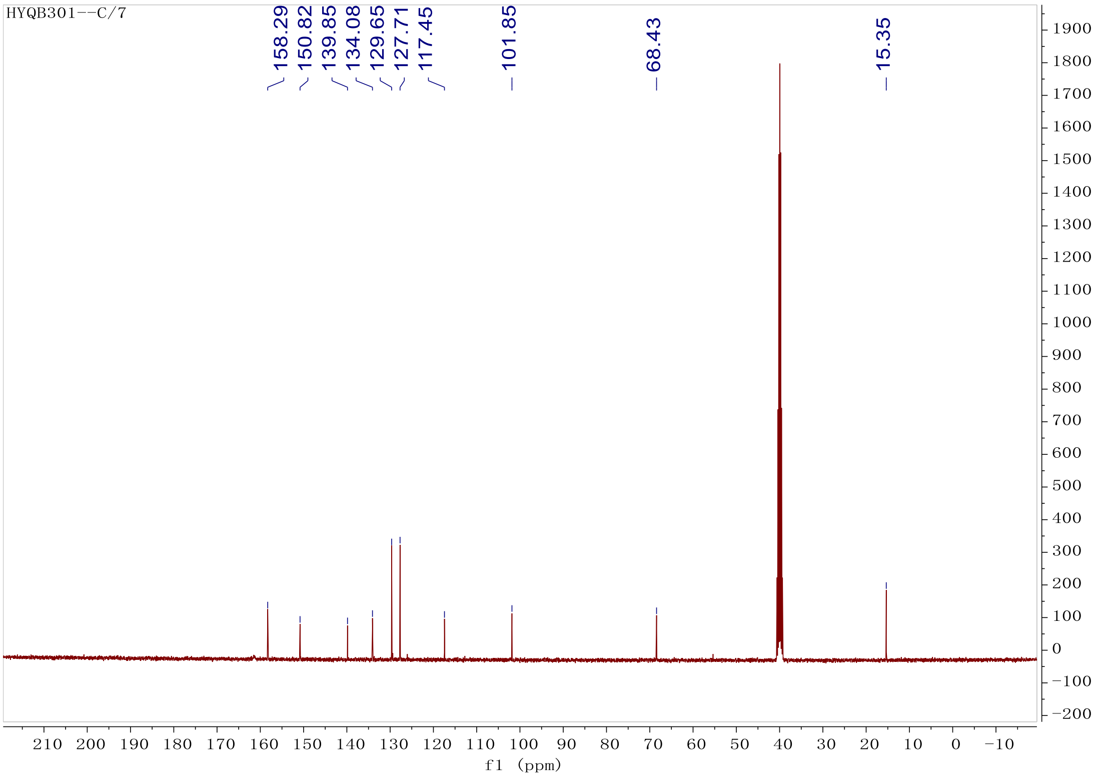
HYQB301--C/7
158.29
150.82
139.85
134.08
129.65
127.71
117.45
101.85
68.43
15.35
f1 (ppm)

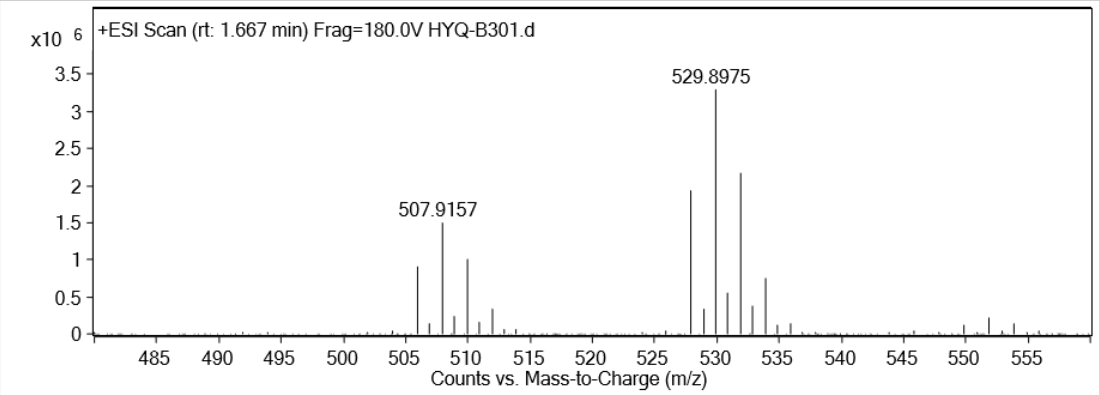

Peak List

m/z	z	Abund
505.9186	1	904639
507.9157	1	1507156.13
509.9129	1	999075
527.9005	1	1932596.88
529.8975	1	3295313
531.8948	1	2177240.5
1034.8093	1	1417809.38
1036.8063	1	2210369.5
1038.8035	1	2029533.25
1040.801	1	1222962.25

Compound 12

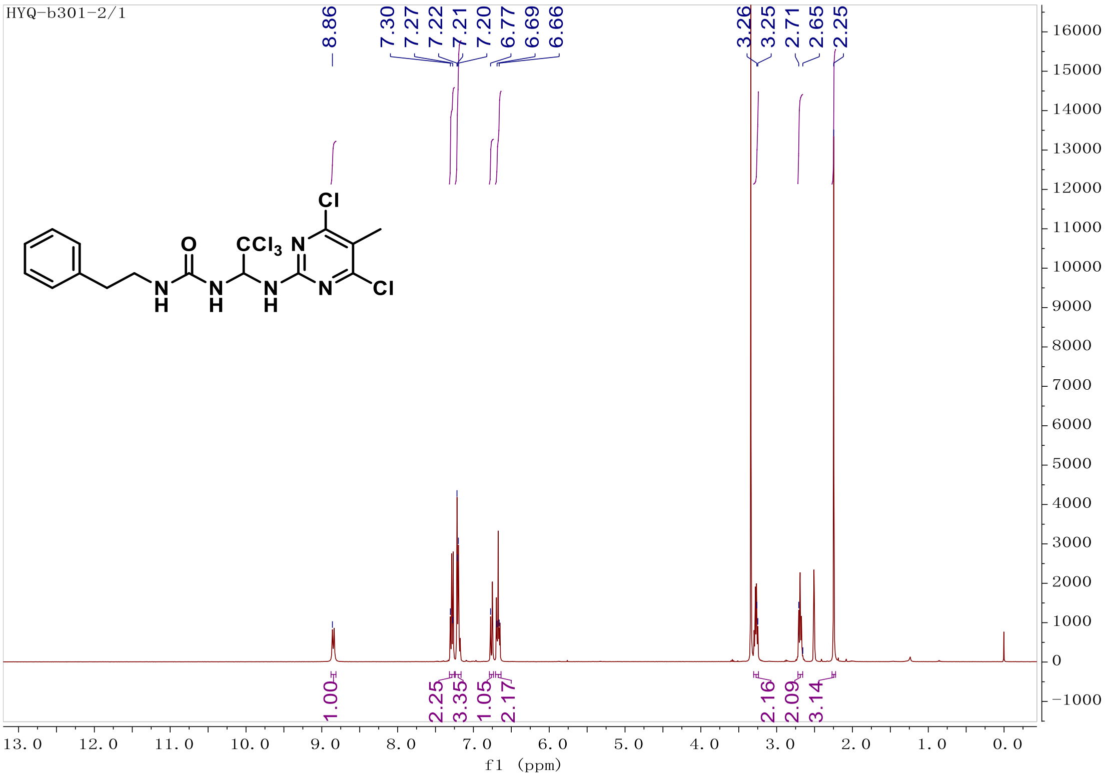

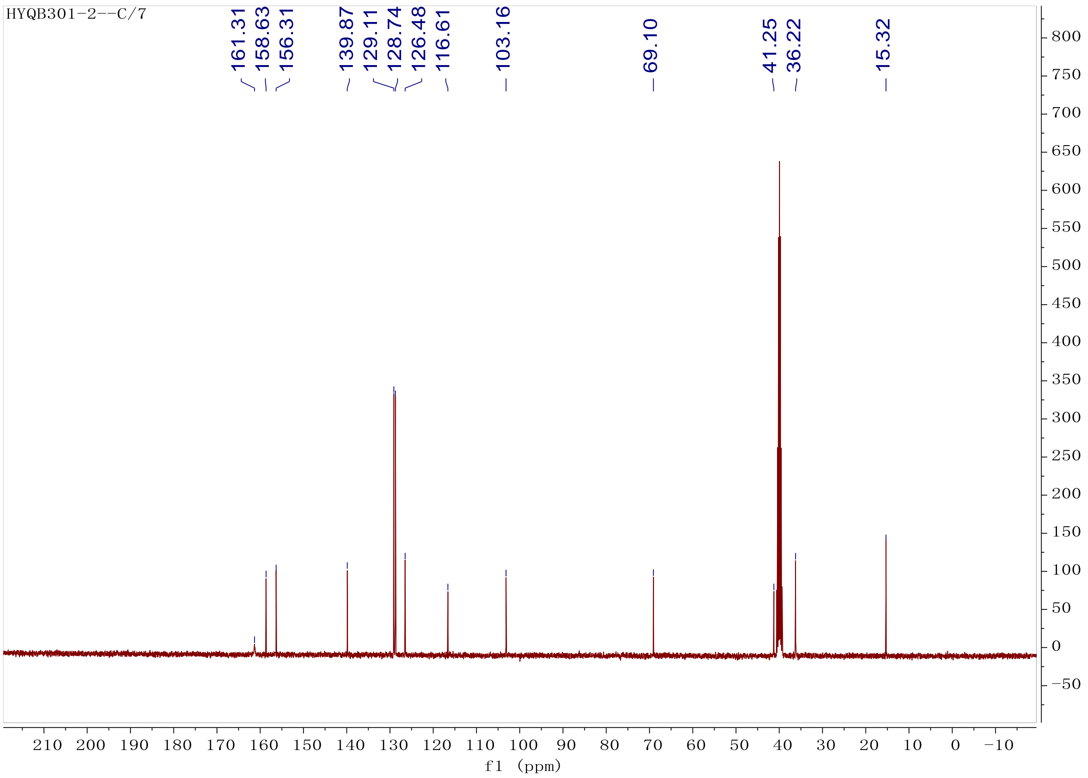

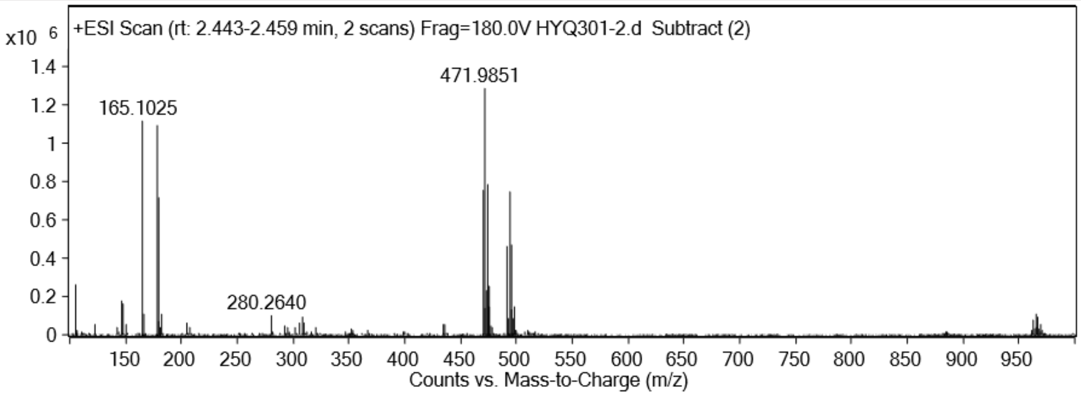

Peak List

m/z	z	Abund
105.0701	1	258868.42
165.1025	1	1116357.5
177.9936	1	1093033.25
179.9907	1	717894.5
469.988	1	751841
471.9851	1	1285317.88
473.9825	1	787652.13
491.9697	1	458376.5
493.9671	1	747647.38
495.9641	1	472444.06

Compound 13

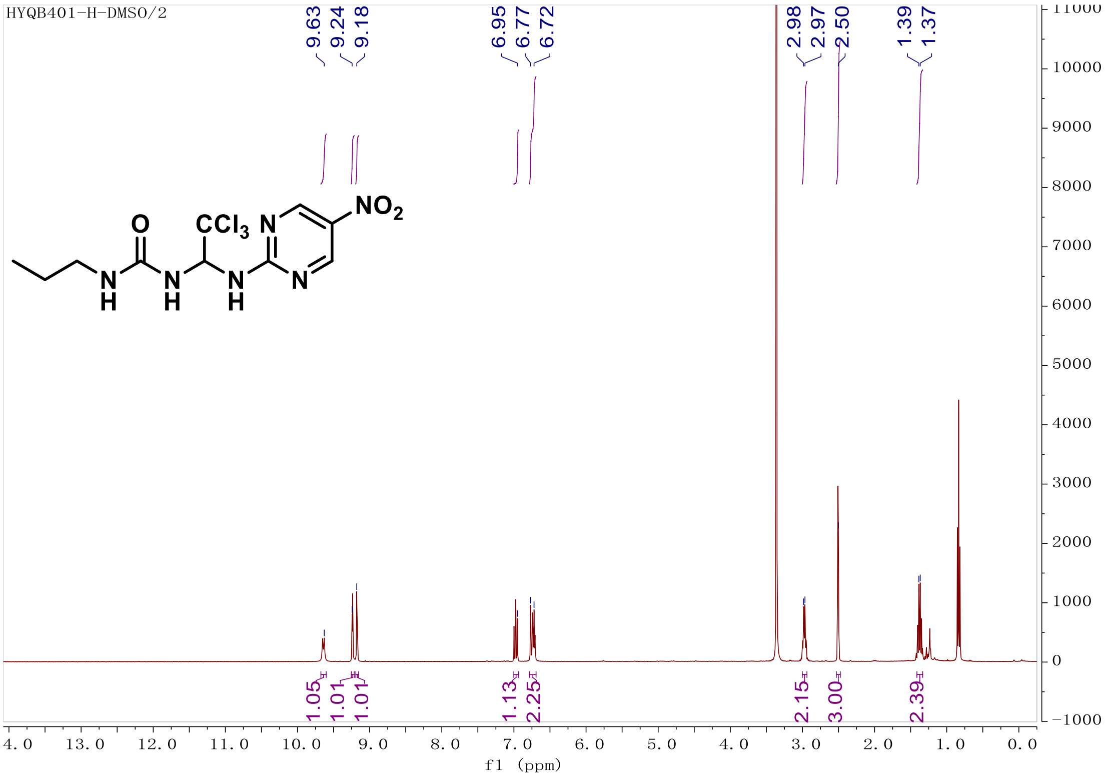

HYQB401-H-DMSO/2
9.63
9.24
9.18
6.95
6.77
6.72
2.98
2.97
2.50
1.39
1.37
1.05
1.01
1.01
1.13
2.25
2.15
3.00
2.39
f1 (ppm)

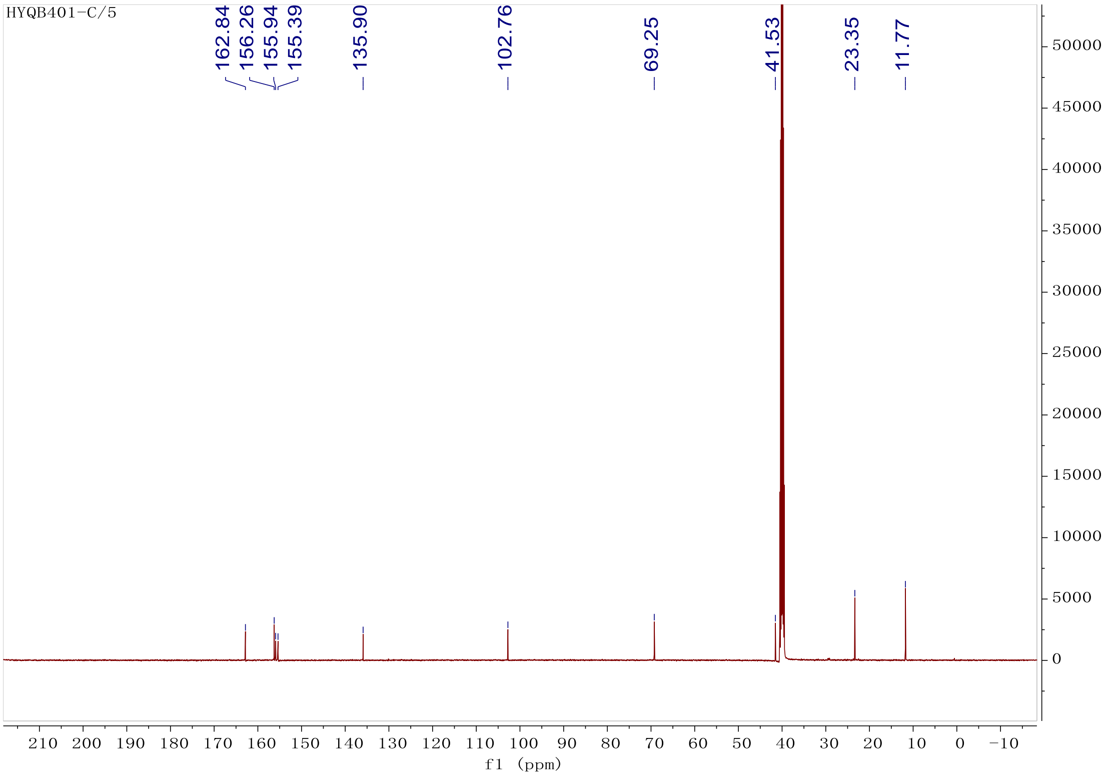

HYQB401-C/5
162.84
156.26
155.94
155.39
135.90
102.76
69.25
41.53
23.35
11.77
f1 (ppm)

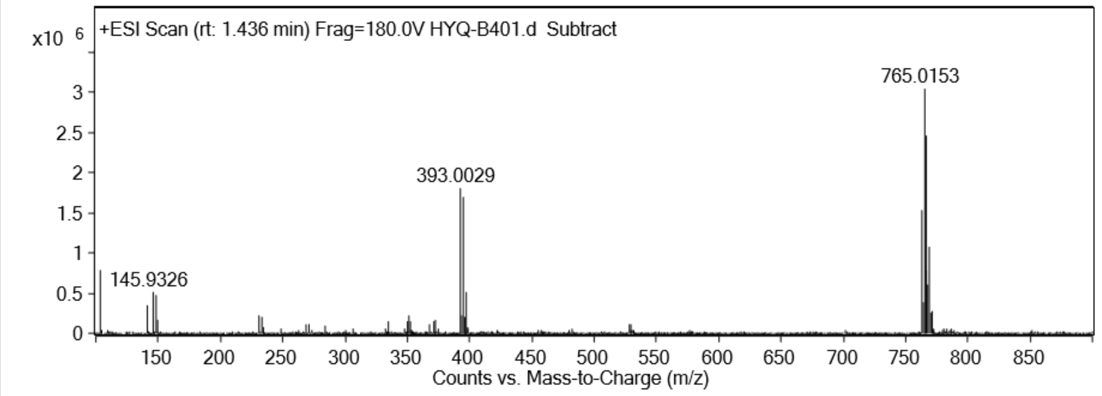

Peak List

m/z	z	Abund
103.0863	1	785083.38
393.0029	1	1802676.75
395.0001	1	1698995.75
396.9976	1	506862.19
763.018	1	1532030.25
765.0153	1	3043747.75
766.0179	1	781276.63
767.0126	1	2451718.75
768.0154	1	607341.31
769.0099	1	1074278.25

Compound 14

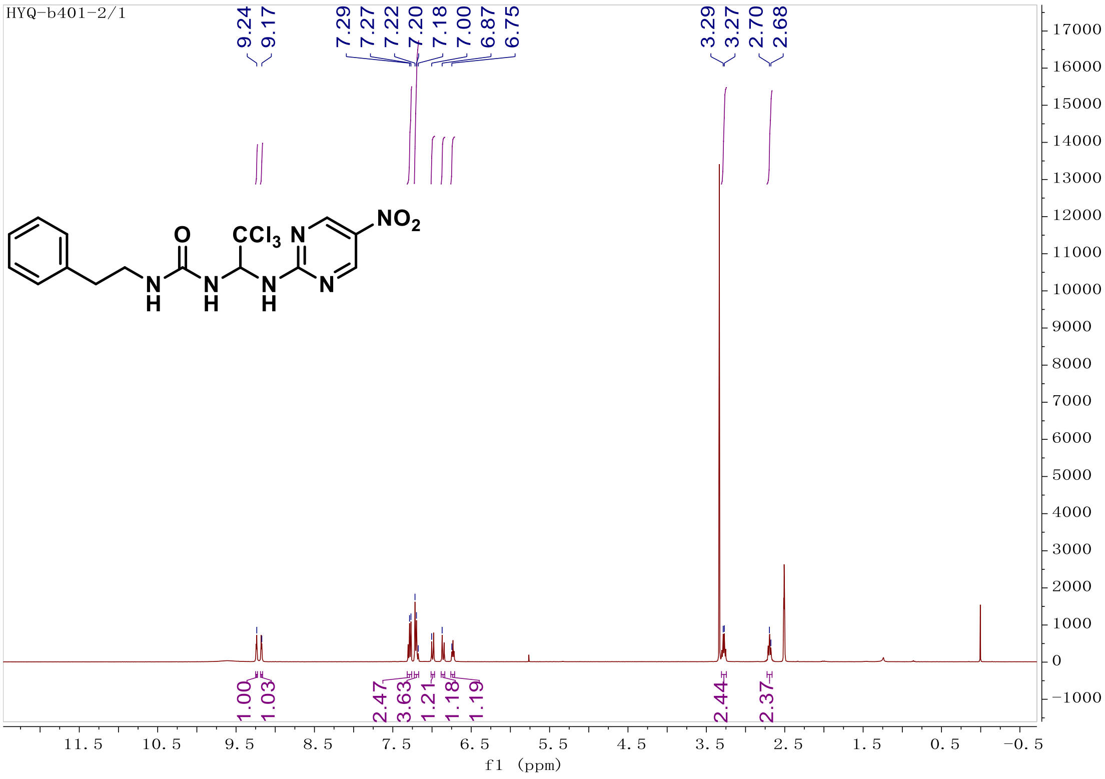

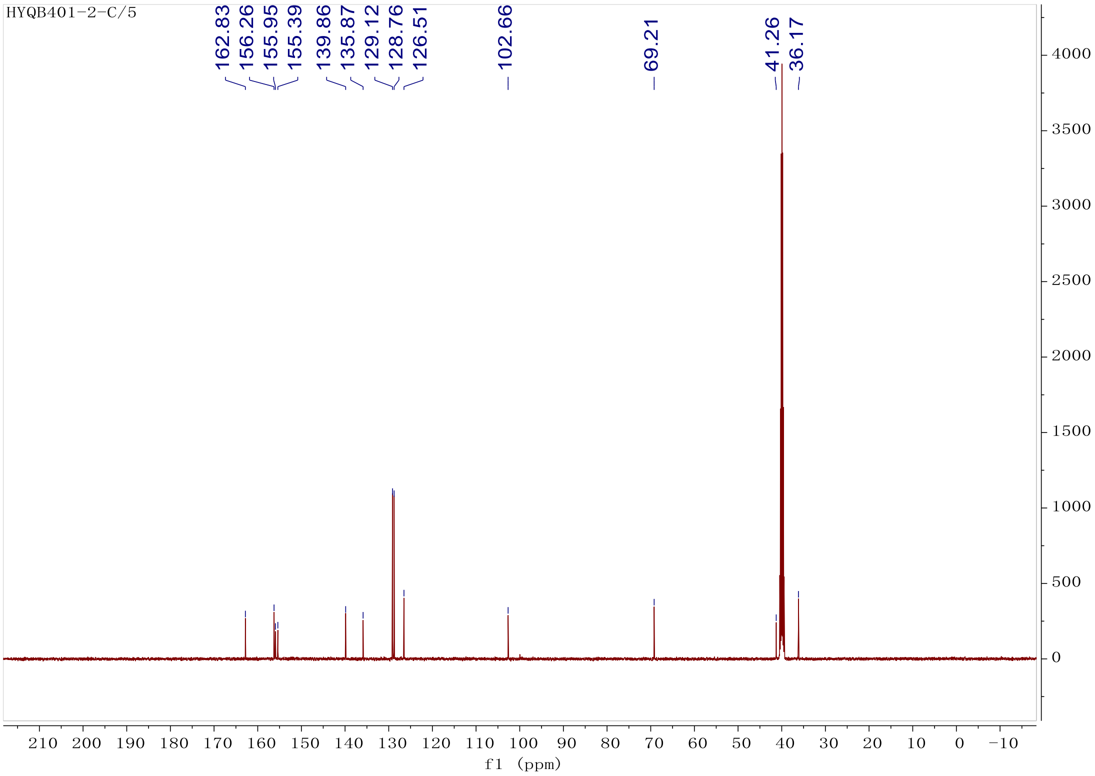

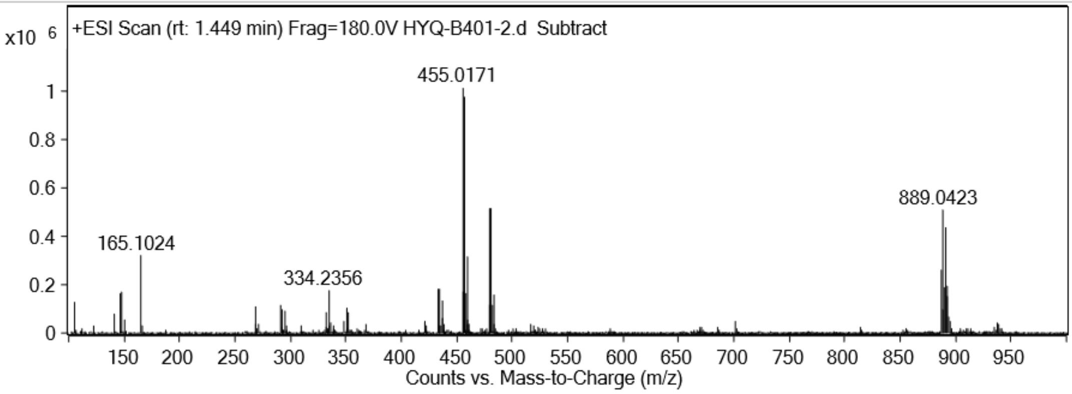

Peak List

m/z	z	Abund
165.1024	1	322023.72
455.0171	1	1016076.06
457.0144	1	978840.63
459.0118	1	315818.06
479.0787	1	517871.5
481.076	1	517401.22
887.0441	1	263722.22
889.0423	1	513587.41
891.0395	1	437112.5
893.0371	1	197534.16

Compound 15

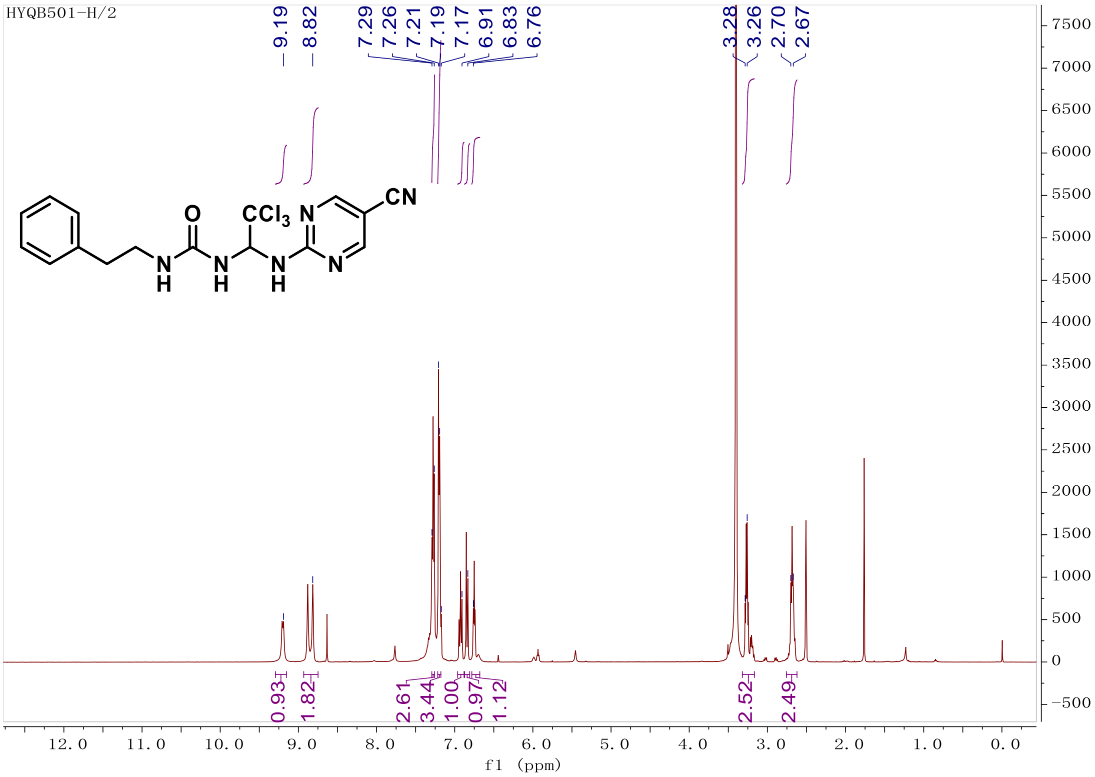
HYQB501-H/2
9.19
8.82
7.29
7.26
7.21
7.19
7.17
6.91
6.83
6.76
3.28
3.26
2.70
2.67
0.93
1.82
2.61
3.44
1.00
0.97
1.12
2.52
2.49
f1 (ppm)

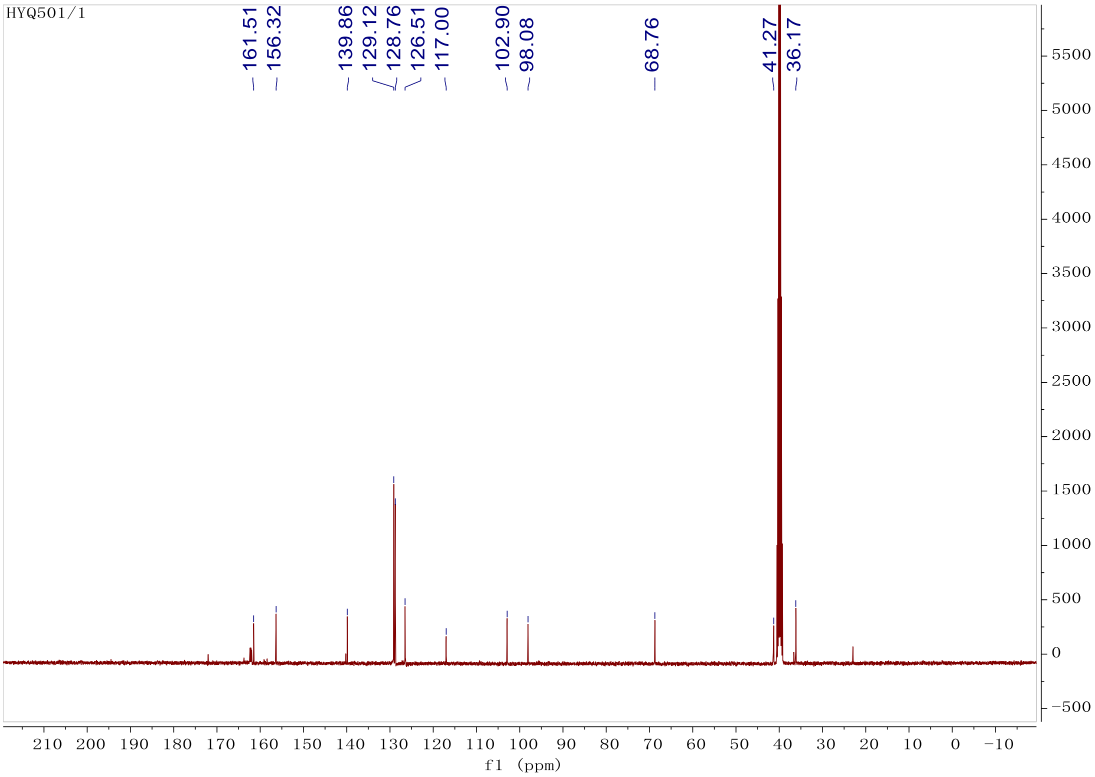
HYQ501/1
161.51
156.32
139.86
129.12
128.76
126.51
117.00
102.90
98.08
68.76
41.27
36.17
f1 (ppm)

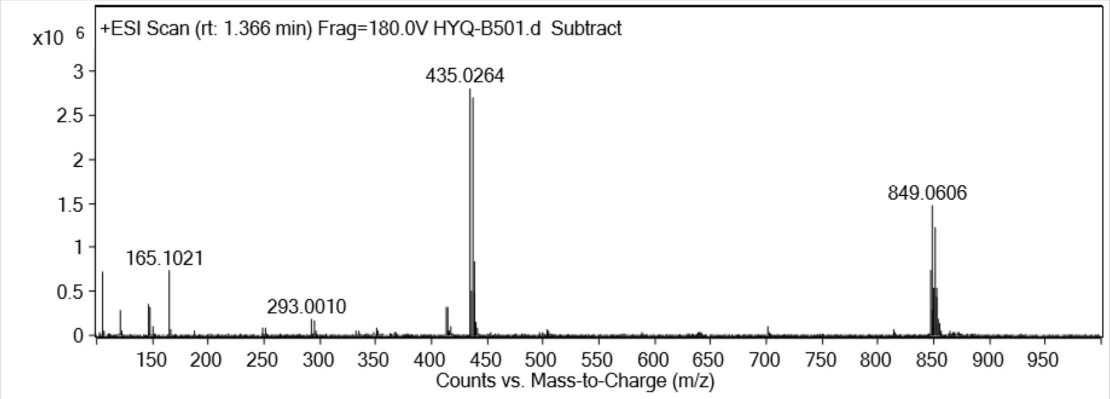

Peak List

m/z	z	Abund
105.0699	1	720554.63
165.1021	1	745874.38
435.0264	1	2805262.5
437.0235	1	2700237.5
439.0211	1	841481.81
847.0633	1	747202.5
849.0606	1	1470491.38
850.0632	1	543118.44
851.0579	1	1228079.75
853.0561	1	542665.75

Compound 16

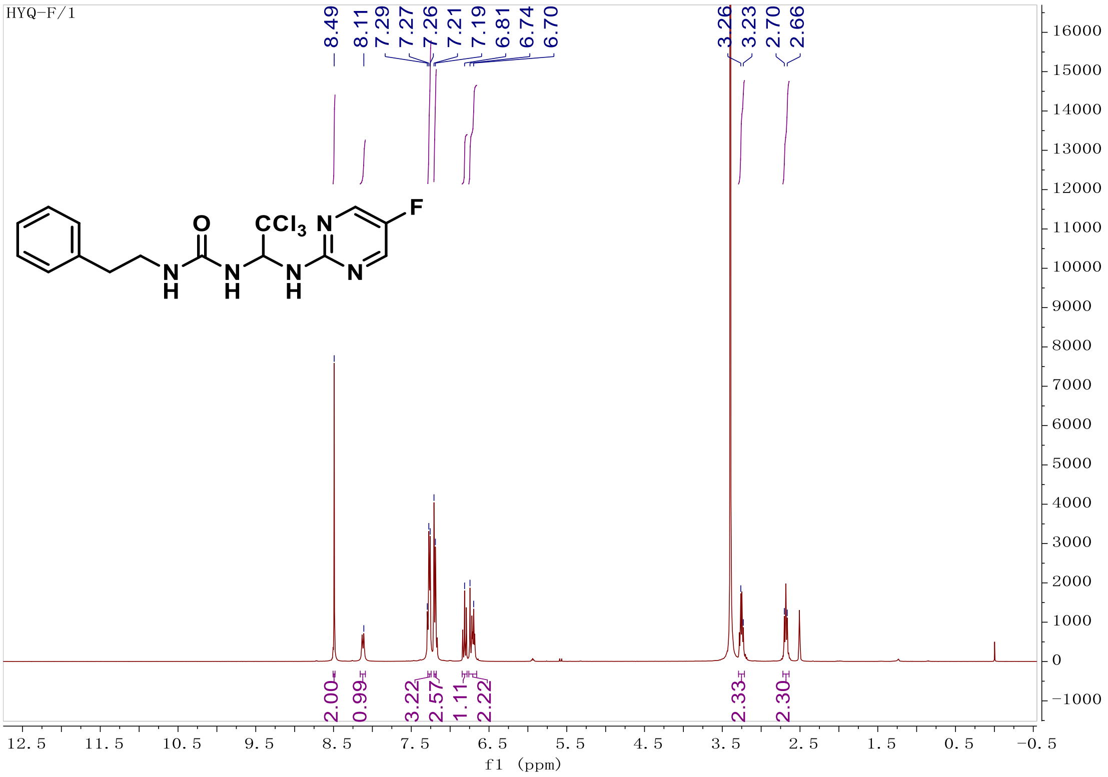

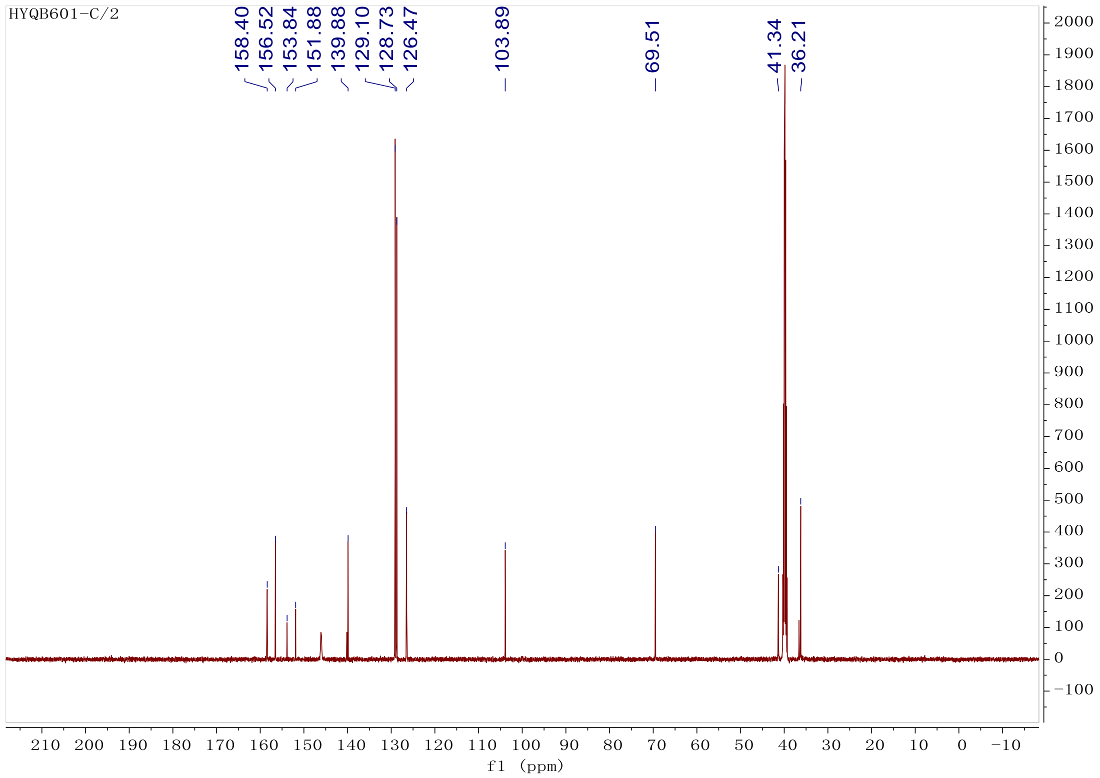

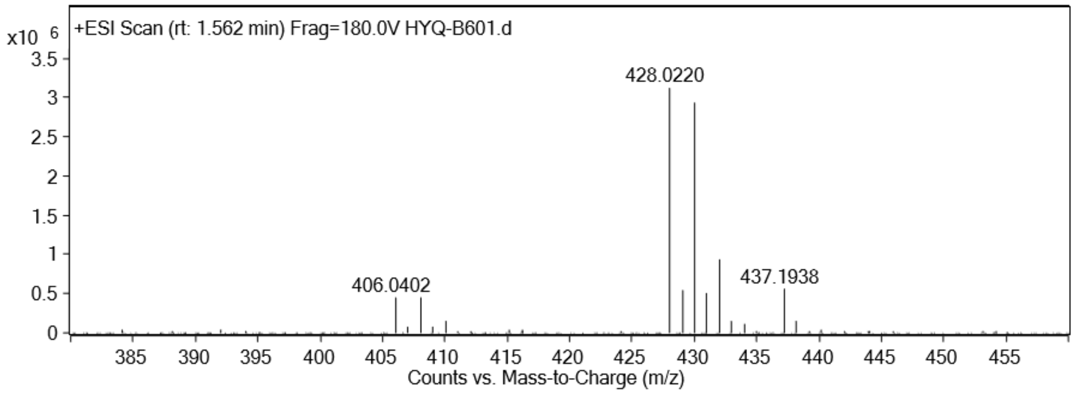

Peak List

m/z	z	Abund
105.0701	1	888271.75
114.0463	1	2514536.25
165.1023	1	2212465.25
269.1652	1	1812408.88
291.1471	1	1649376.88
428.022	1	3121466.25
430.0191	1	2925209.5
432.0166	1	933158.81
835.0517	1	1677685.13
837.0493	1	1391531.63

Compound 17

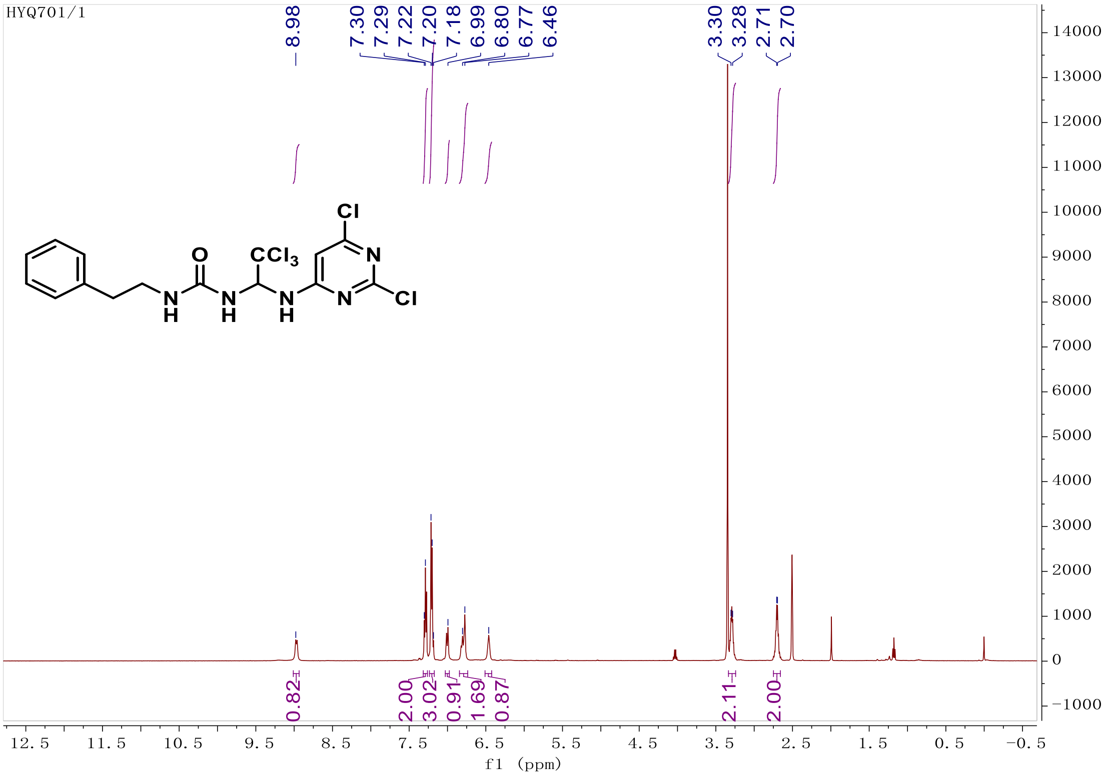

HYQ701/1
8.98
7.30
7.29
7.22
7.20
7.18
6.99
6.80
6.77
6.46
3.30
3.28
2.71
2.70
0.82
2.00
3.02
0.91
1.69
0.87
2.11
2.00
f1 (ppm)

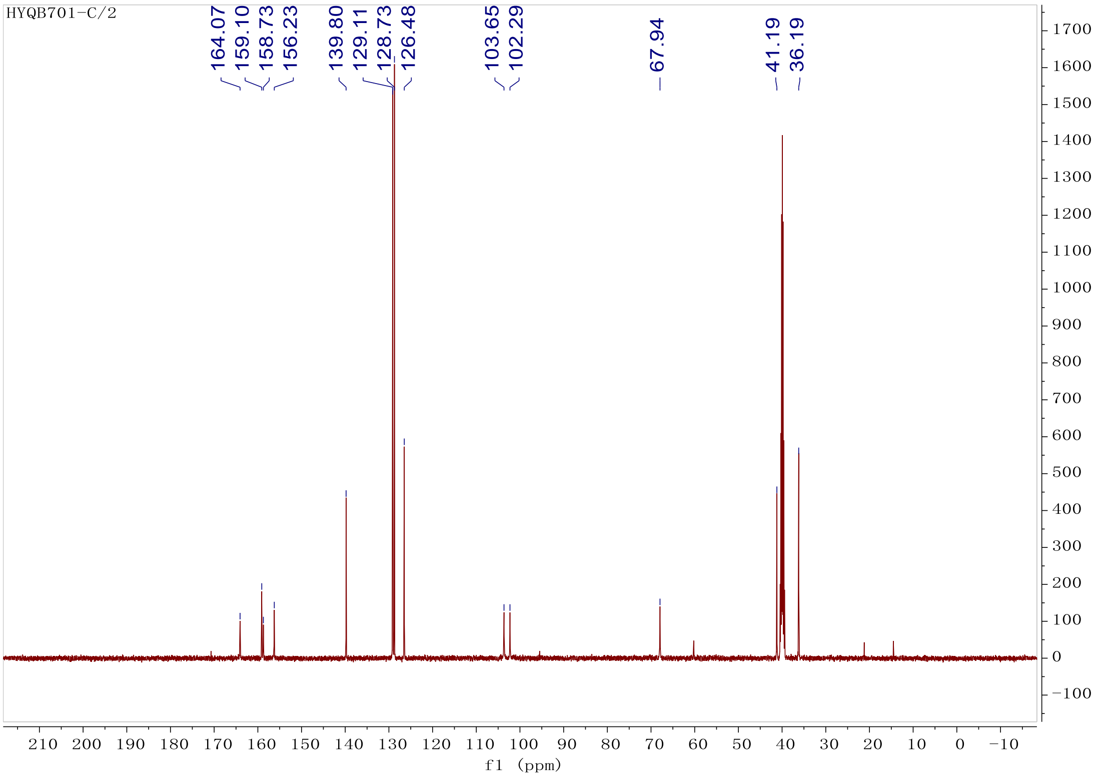

HYQB701-C/2
164.07
159.10
158.73
156.23
139.80
129.11
128.73
126.48
103.65
102.29
67.94
41.19
36.19
f1 (ppm)

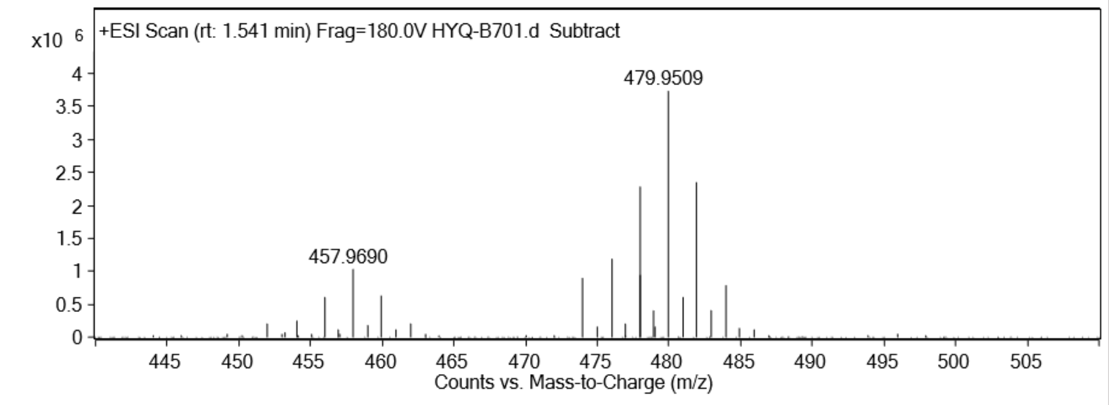

Peak List

m/z	z	Abund
437.1941	1	909386.56
457.969	1	1038226.06
459.9663	1	633230
474.0032	1	894705.75
476.0005	1	1187687.75
477.9537	1	2292353
477.9952	1	947097.63
479.9509	1	3737555
481.9478	1	2353788.5
483.9454	1	783087.38

Compound 18

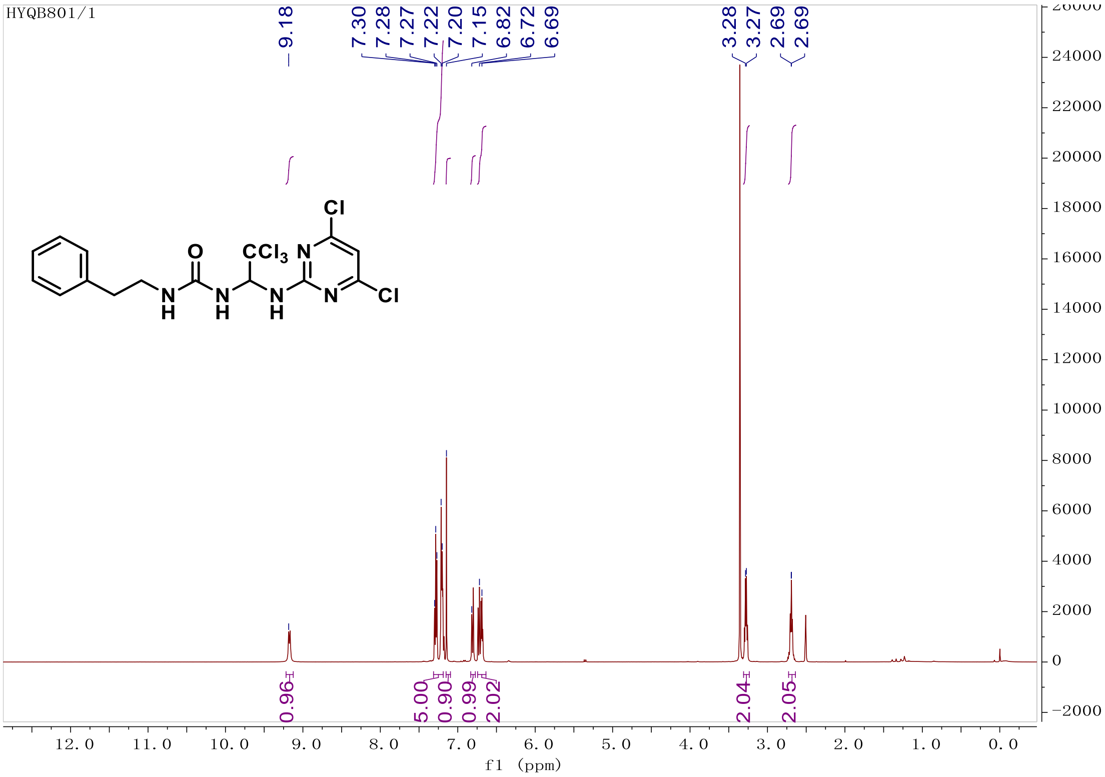

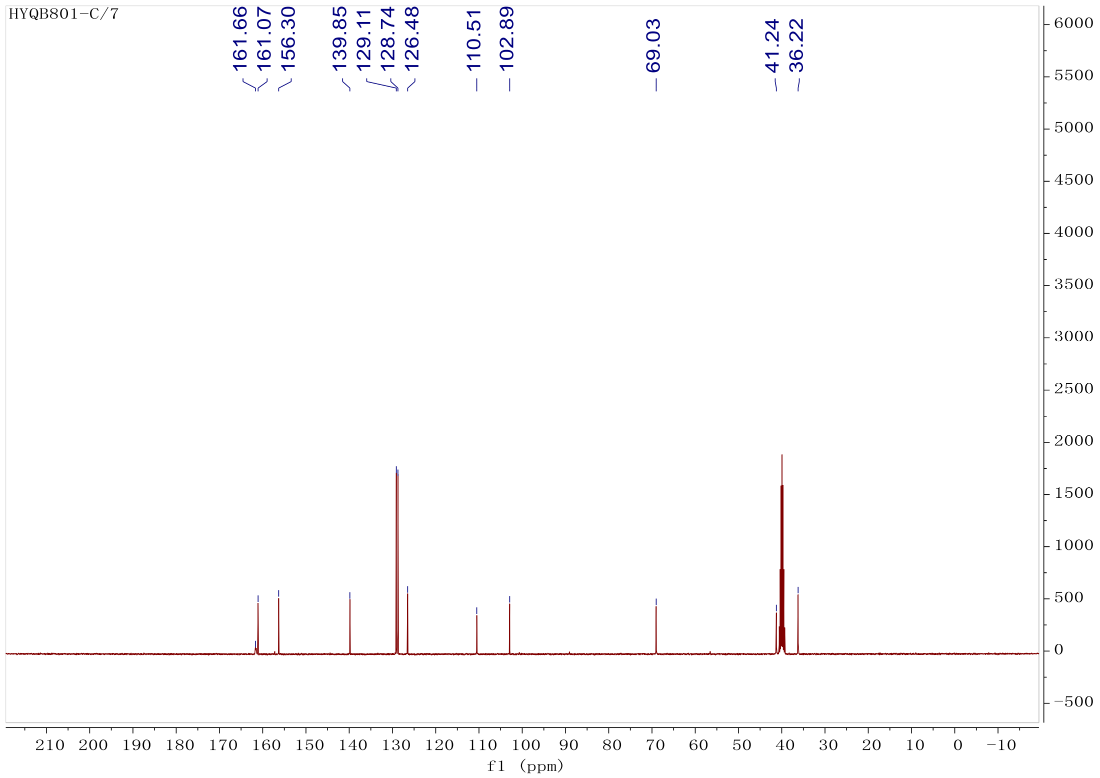

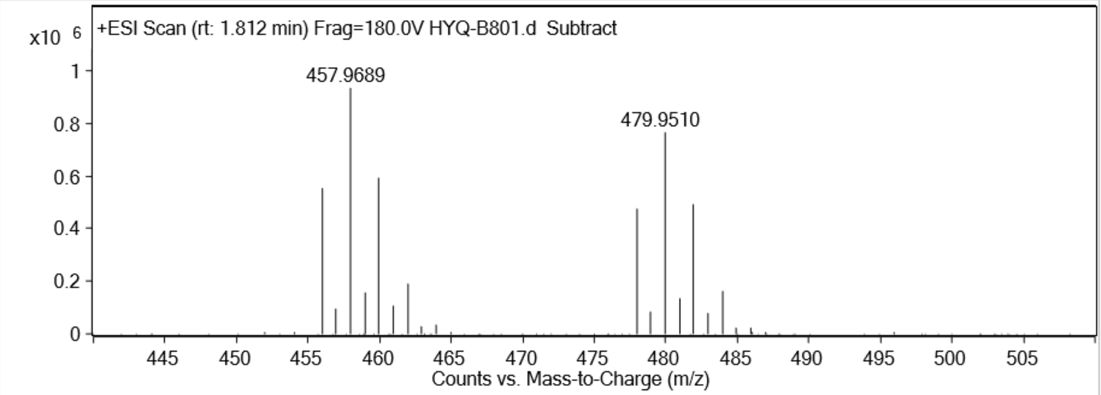

Peak List

m/z	z	Abund
145.9324	1	348627.97
163.9775	1	838498.63
165.1022	1	1191093.75
165.9748	1	496365.72
455.972	1	555500.81
457.9689	1	933681.19
459.9665	1	594948.31
477.9538	1	476758.88
479.951	1	766428
481.9483	1	492323.28

Compound 19

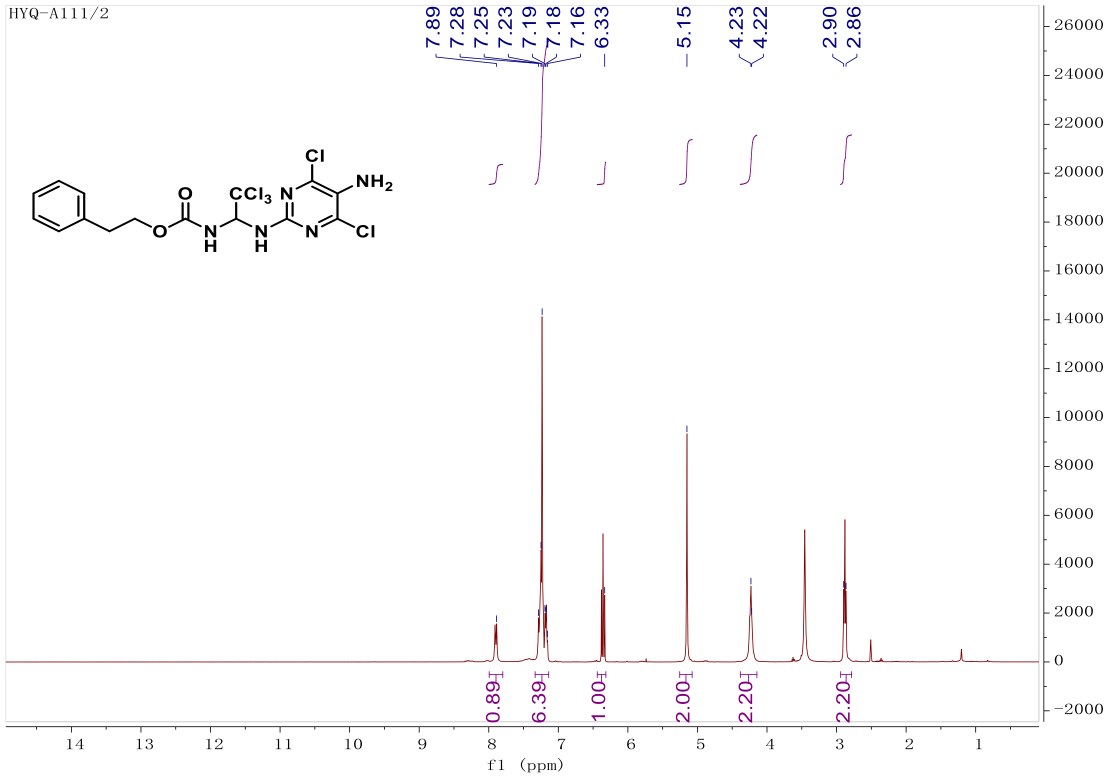
HYQ-A111/2
7.89
7.28
7.25
7.23
7.19
7.18
7.16
6.33
5.15
4.23
4.22
2.90
2.86
Cl
NH2
O
CCl3
N
O
N
H
N
H
N
Cl
0.89
6.39
1.00
2.00
2.20
2.20
f1 (ppm)

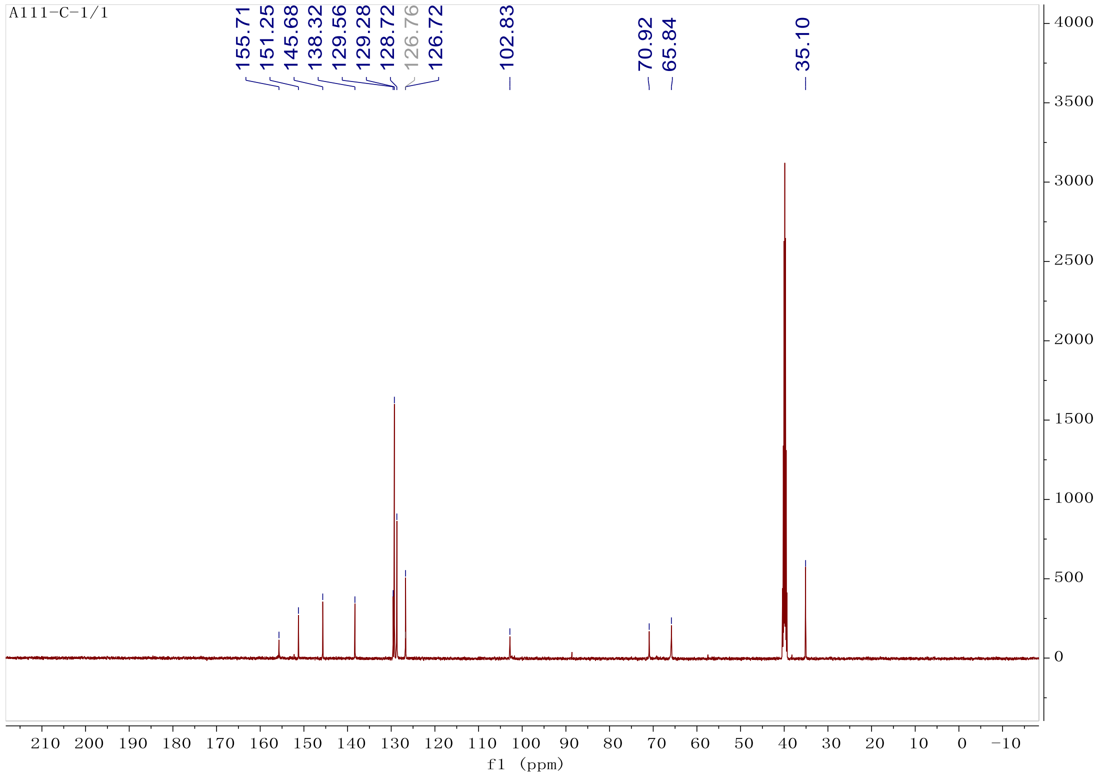
A111-C-1/1
155.71
151.25
145.68
138.32
129.56
129.28
128.72
126.76
126.72
102.83
70.92
65.84
35.10
f1 (ppm)

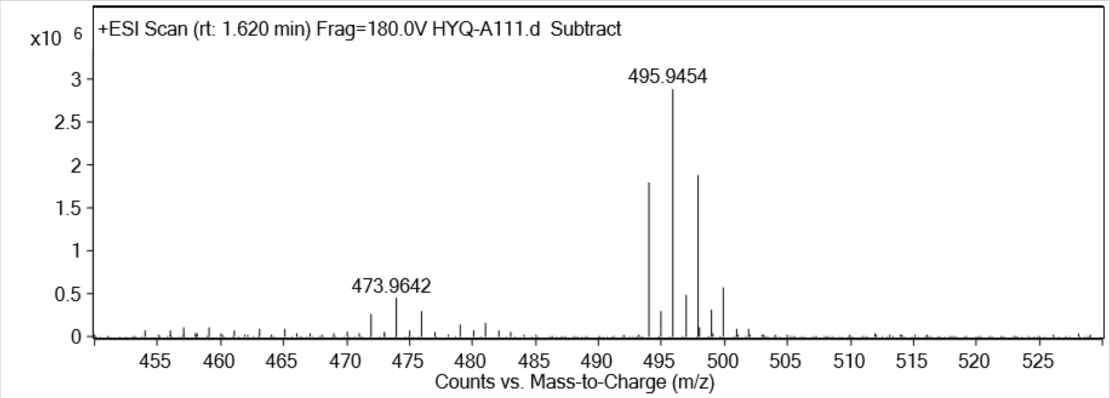

Peak List

m/z	z	Abund
105.0695	1	820466.38
178.9881	1	964302.94
180.9853	1	559908.38
306.8872	1	1546314.75
308.8842	1	2504377.25
310.8813	1	1548836.63
493.9483	1	1801089.38
495.9454	1	2881409.5
497.9427	1	1872262.88
499.9401	1	564606.31

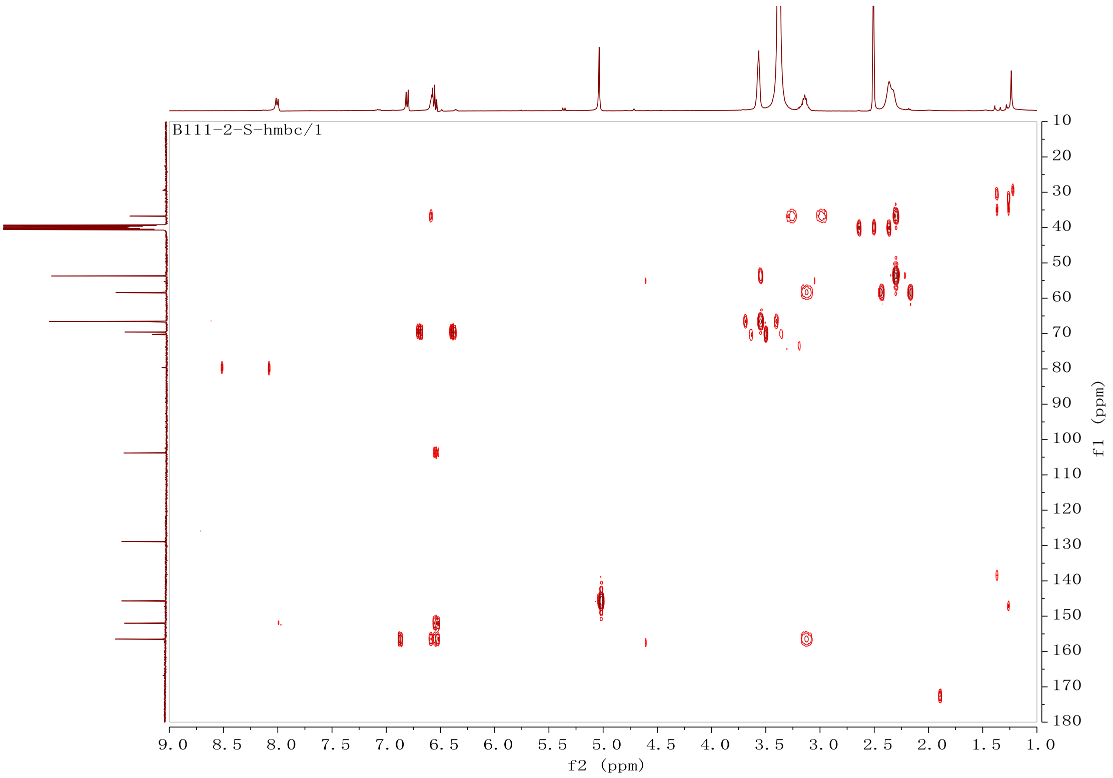

Compound 20

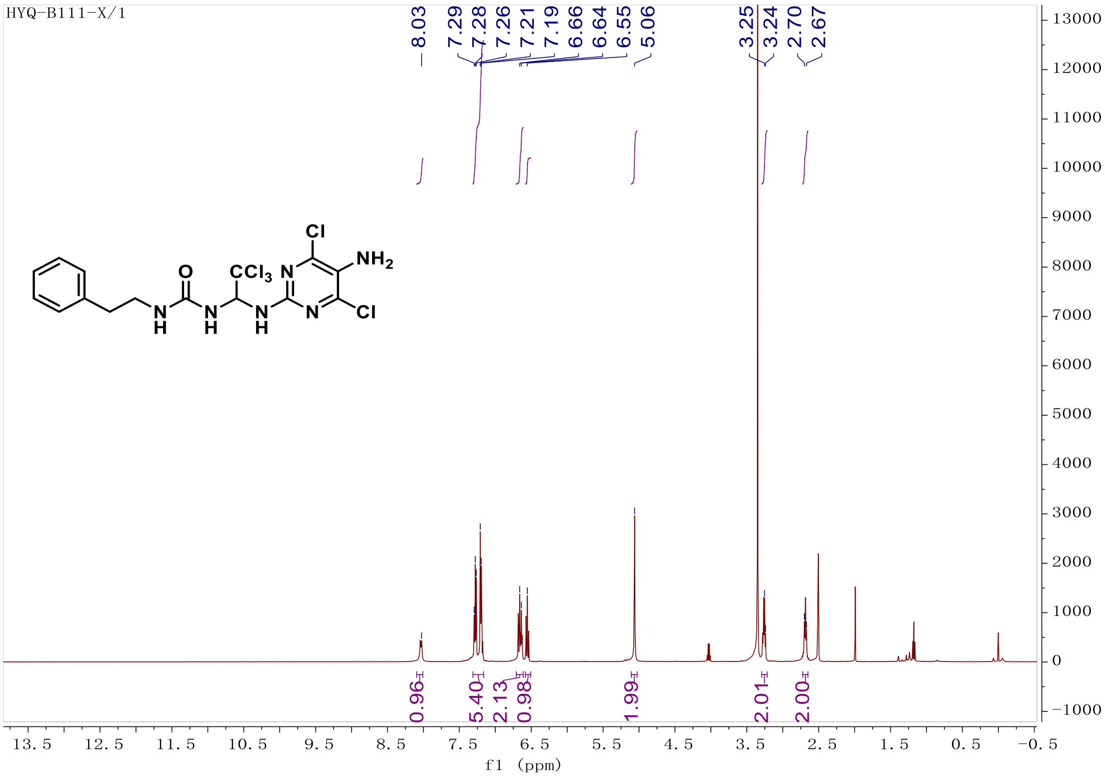
HYQ-B111-X/1
8.03
7.29
7.28
7.26
7.21
7.19
6.66
6.64
6.55
5.06
3.25
3.24
2.70
2.67
0.96
5.40
2.13
0.98
1.99
2.01
2.00
f1 (ppm)

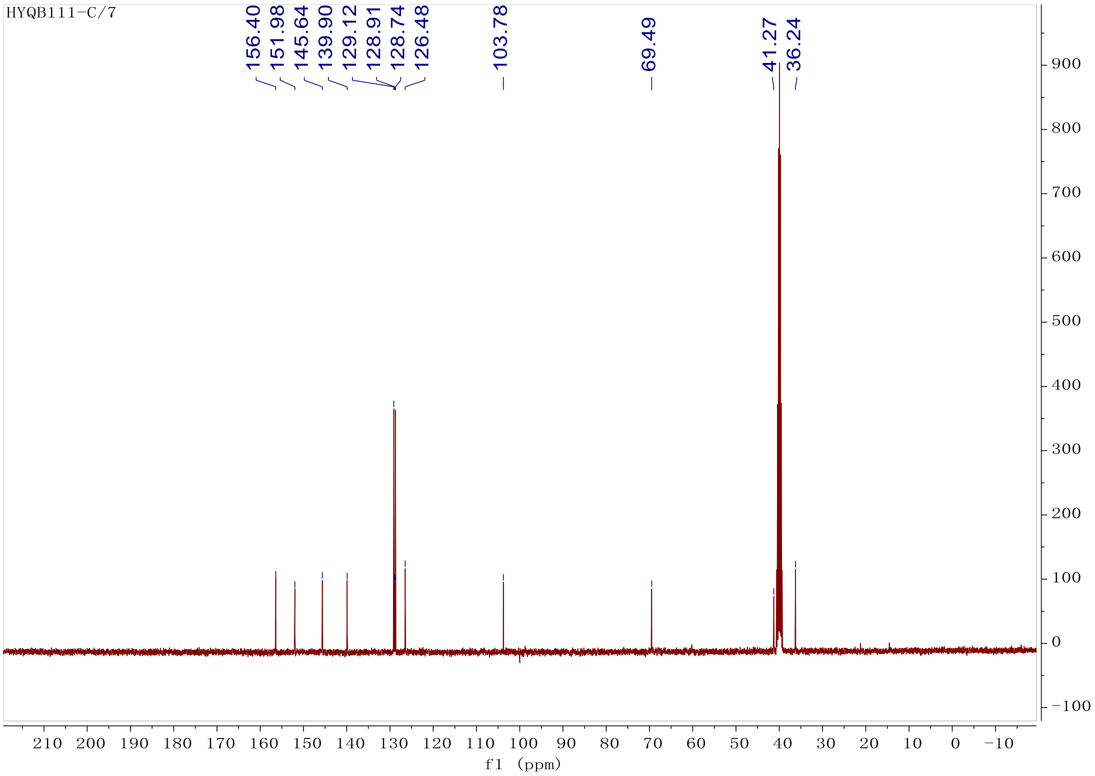
HYQB111-C/7
156.40
151.98
145.64
139.90
129.12
128.91
128.74
126.48
103.78
69.49
41.27
36.24
f1 (ppm)

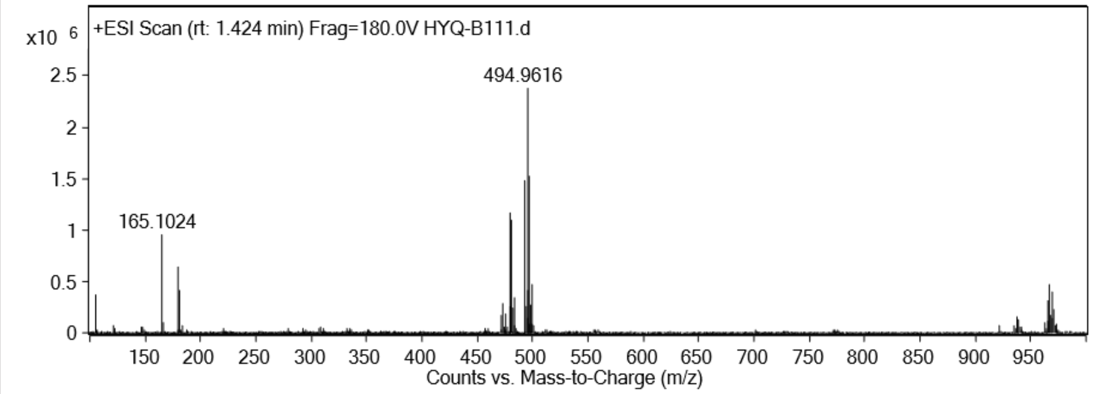

Peak List

m/z	z	Abund
165.1024	1	959769.5
178.9887	1	642487.75
479.0783	1	1173194.88
481.0756	1	1094816.75
492.9643	1	1482129.88
494.9616	1	2380009.5
495.9646	1	416777.94
496.9588	1	1527294.75
498.9563	1	474308.16
966.9346	1	463690.53

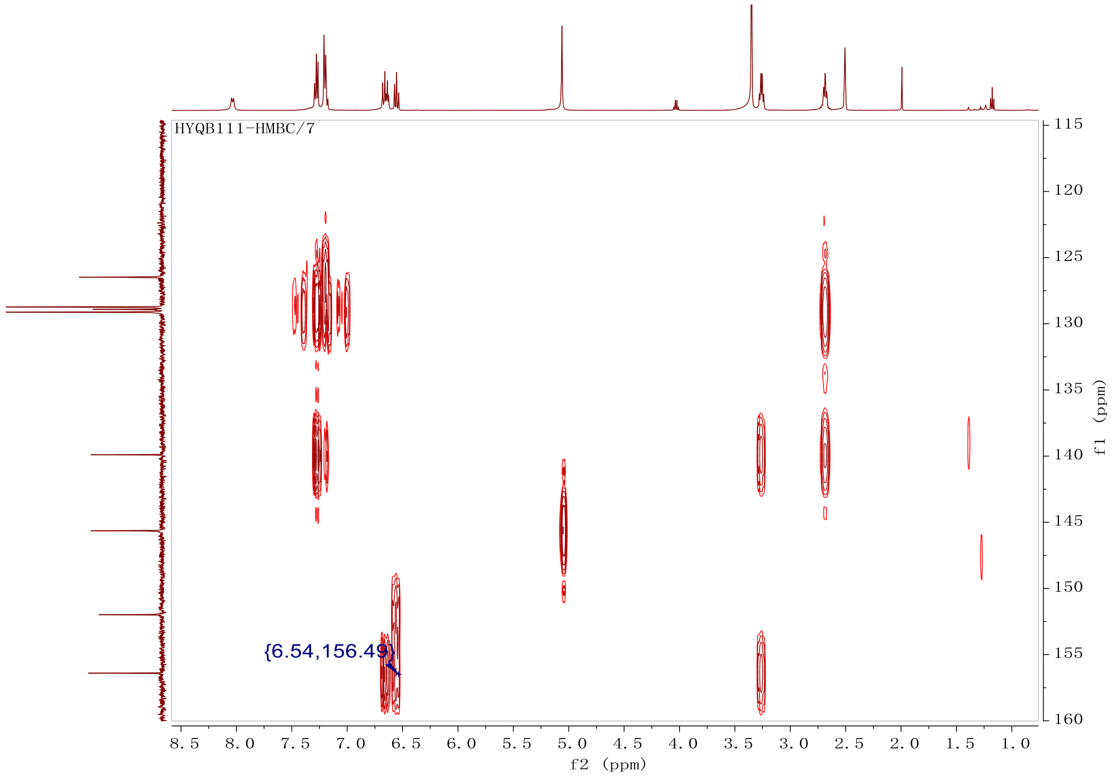

Compound 21

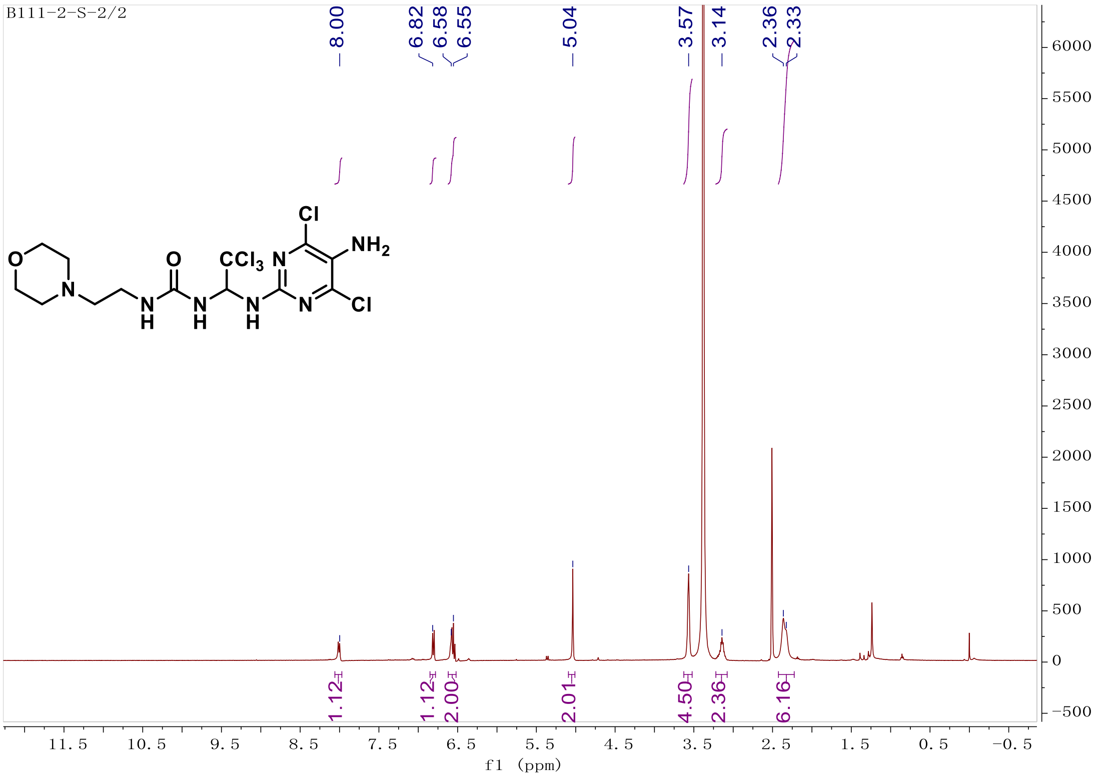
B111-2-S-2/2
8.00
6.82
6.58
6.55
5.04
3.57
3.14
2.36
2.33
1.12
1.12
2.00
2.01
4.50
2.36
6.16
Cl
NH2
O
CCl3
N
N
N
H
Cl
f1 (ppm)

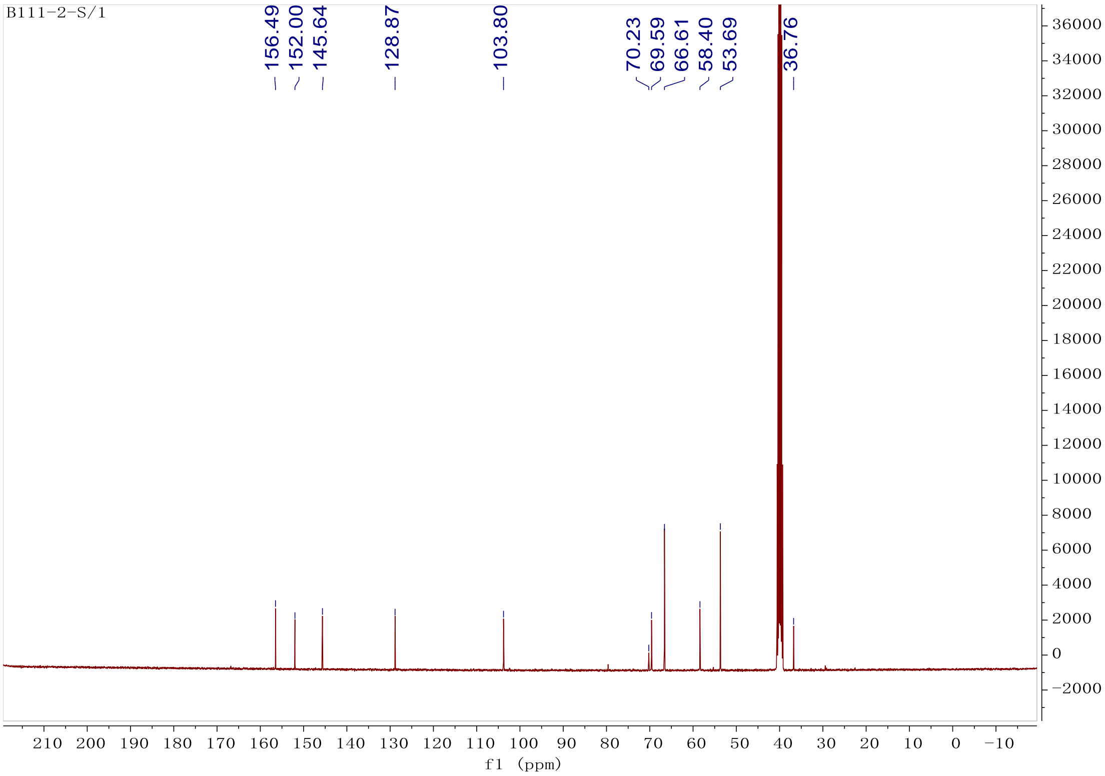
B111-2-S/1
156.49
152.00
145.64
128.87
103.80
70.23
69.59
66.61
58.40
53.69
36.76
f1 (ppm)

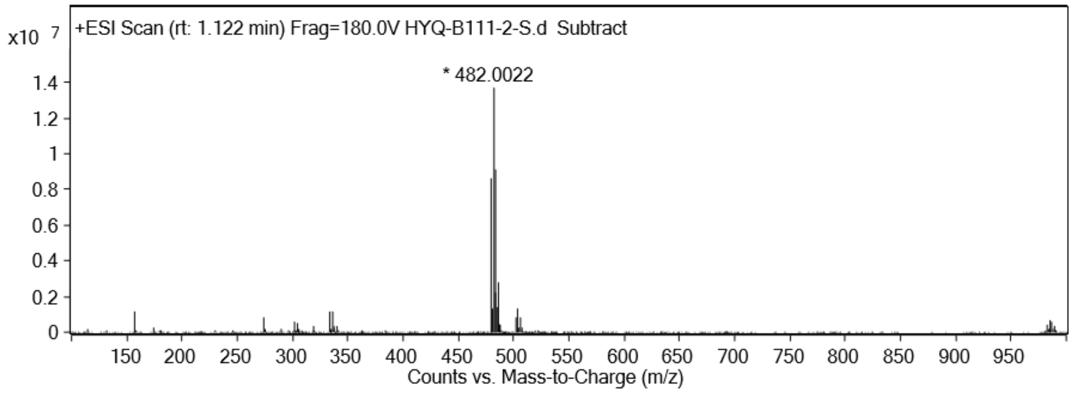

Peak List

m/z	z	Abund
480.0045	1	8586825
482.0022	1	13710828
483.9988	1	9087128
485.9958	1	2827894.25

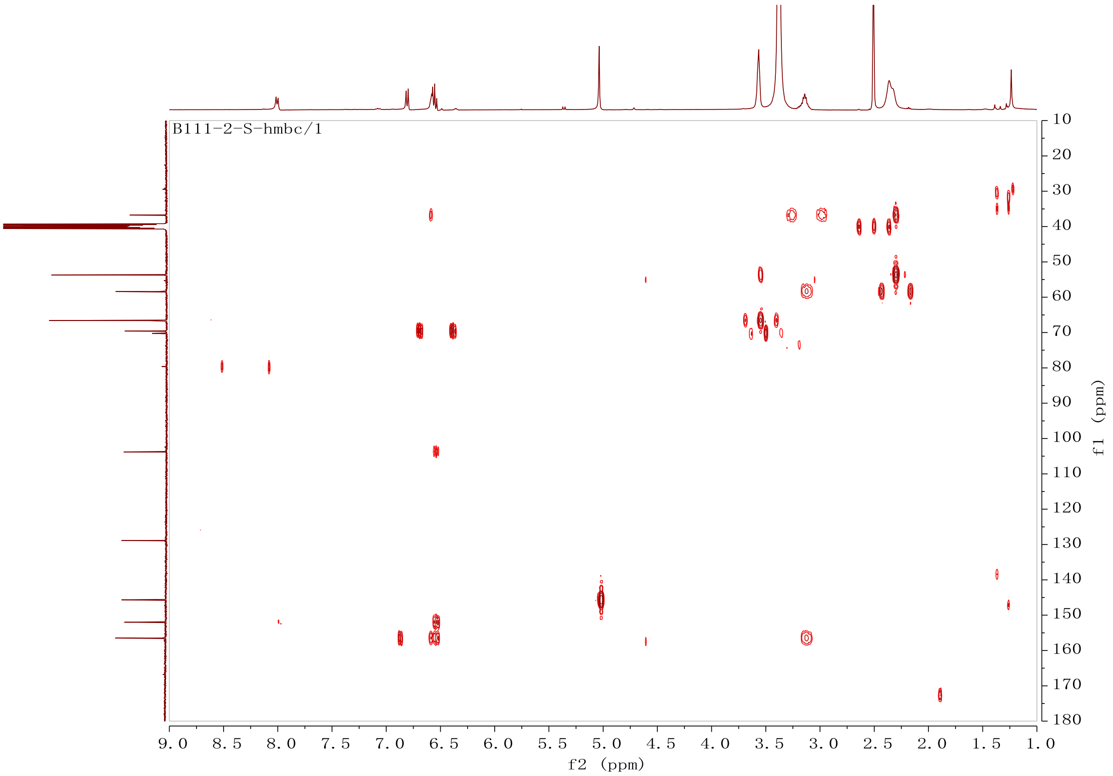

Compound 22

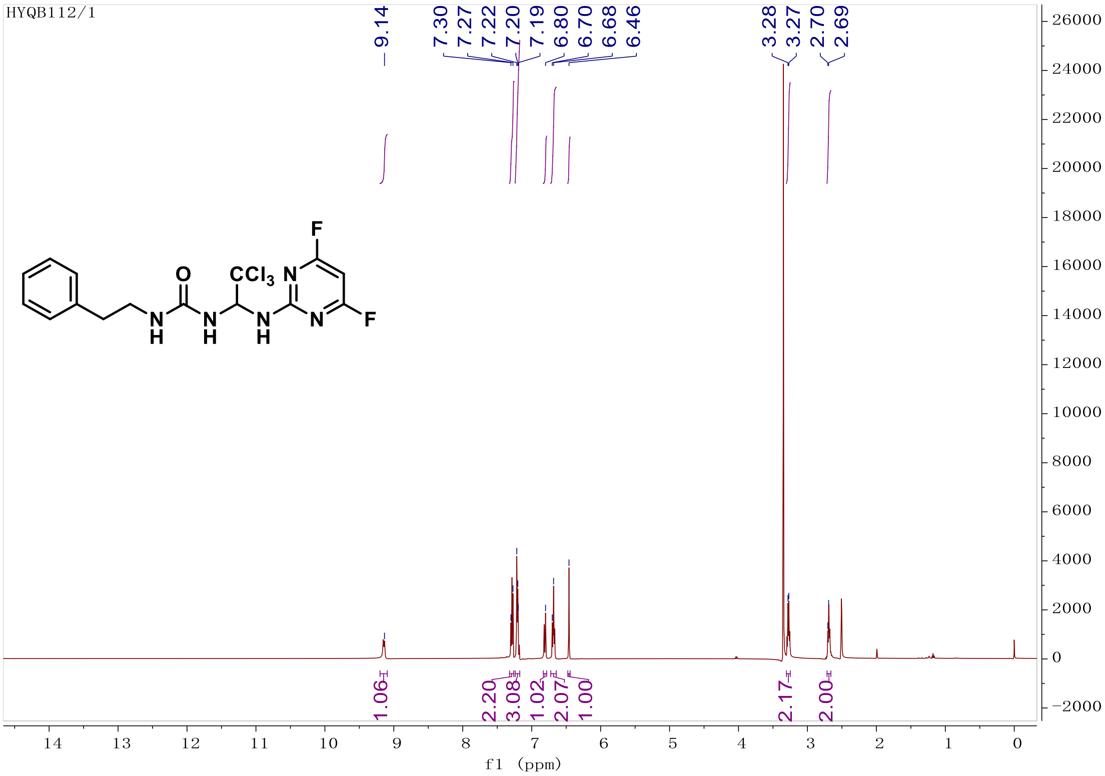
HYQB112/1
9.14
7.30
7.27
7.22
7.20
7.19
6.80
6.70
6.68
6.46
3.28
3.27
2.70
2.69
1.06
2.20
3.08
1.02
2.07
1.00
2.17
2.00
f1 (ppm)

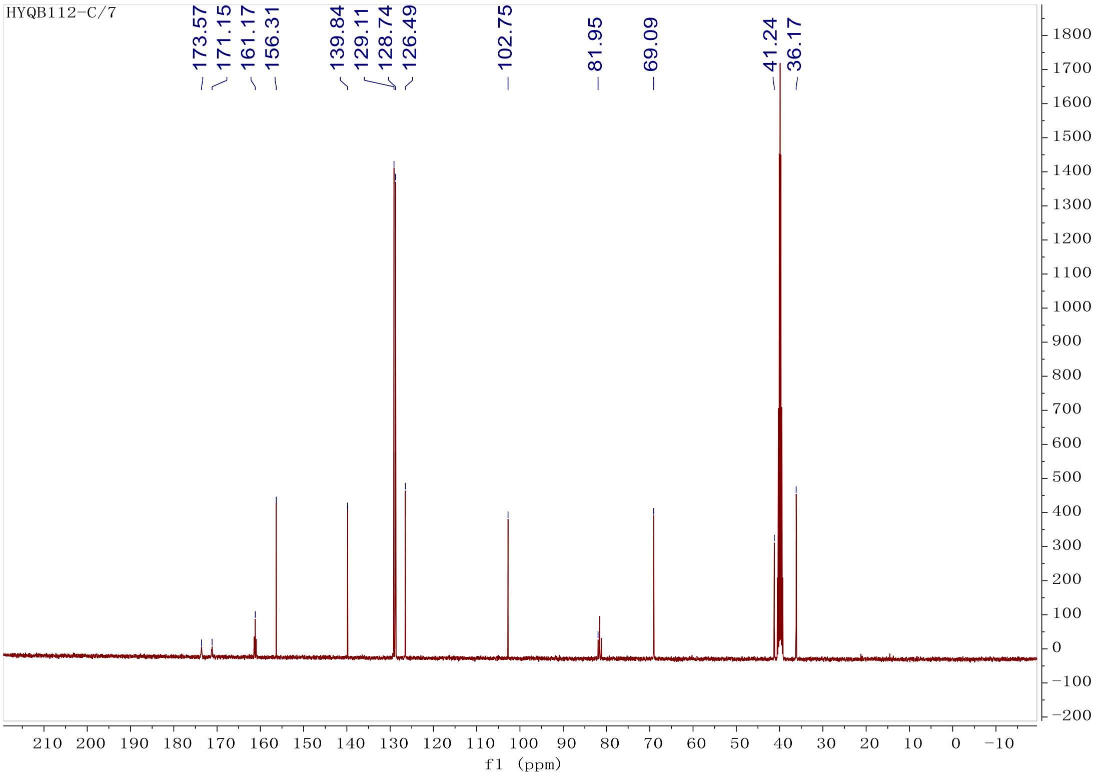
HYQB112-C/7
173.57
171.15
161.17
156.31
139.84
129.11
128.74
126.49
102.75
81.95
69.09
41.24
36.17
f1 (ppm)

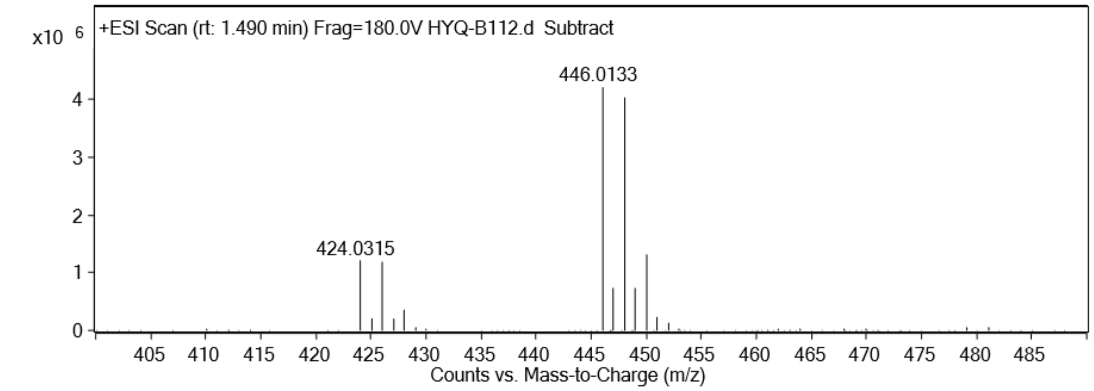

Peak List

m/z	z	Abund
145.9326	1	1976611.5
147.9297	1	1877281
165.1024	1	3386468.5
293.0016	1	1977598.25
294.9988	1	1875690.88
424.0315	1	1224144.75
426.0287	1	1186664.63
446.0133	1	4218536.5
448.0106	1	4041666.5
450.0078	1	1302441

Compound 23

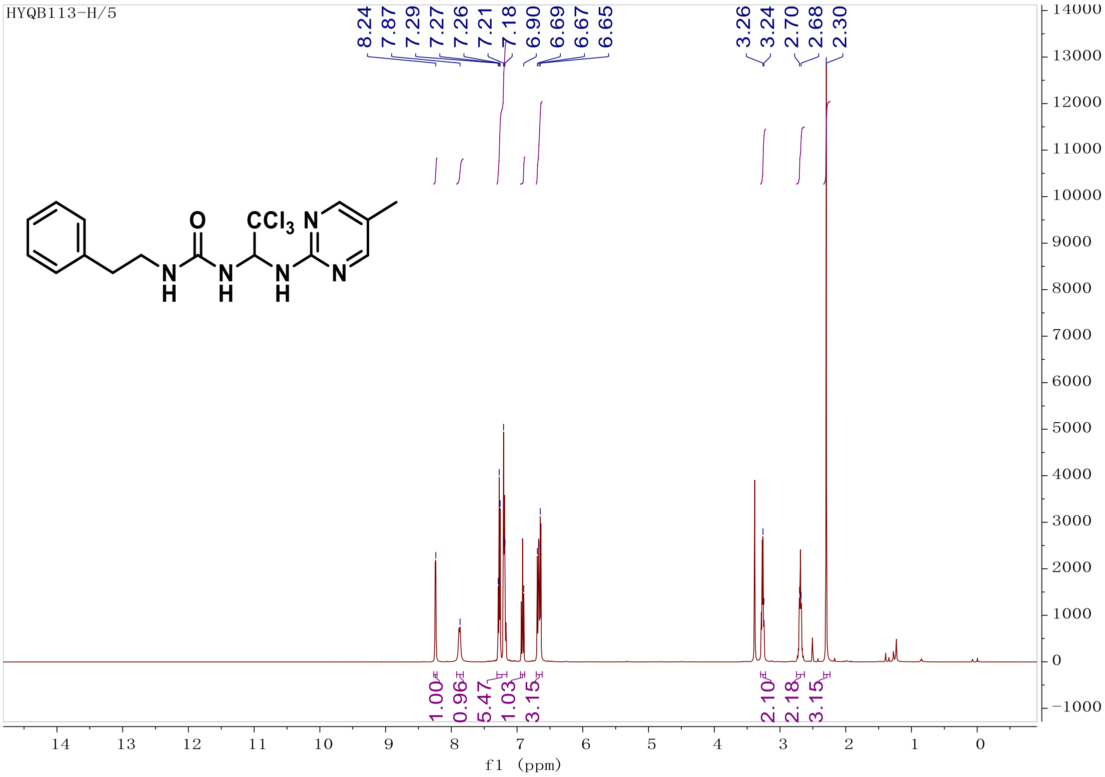

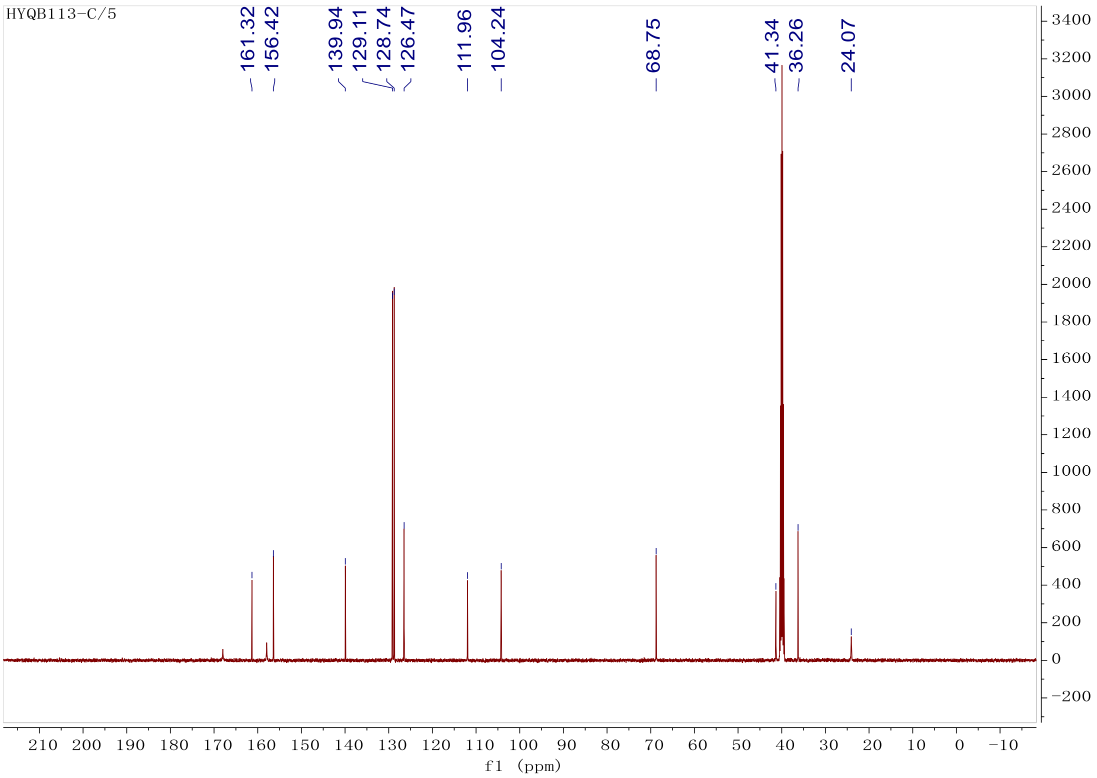

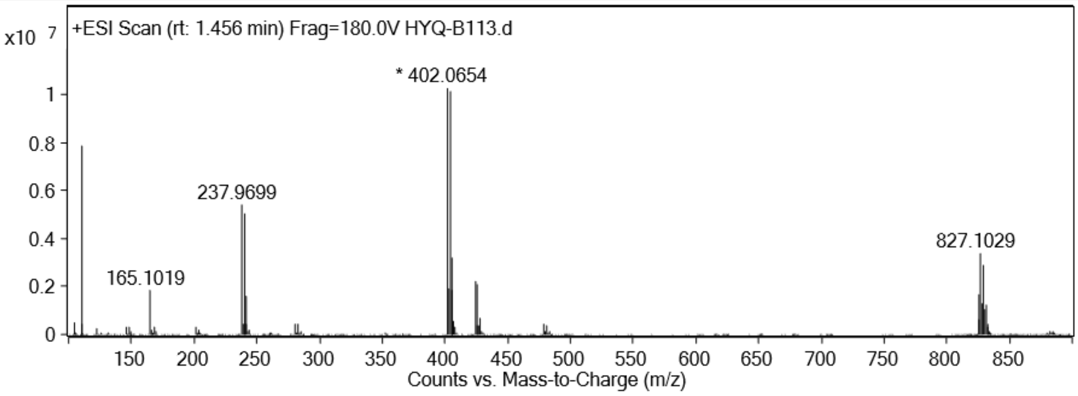

Peak List

m/z	z	Abund
110.0711	1	7882334
237.9699	1	5373167
239.967	1	5044480
402.0654	1	10249819
404.0626	1	10105609
406.0596	1	3168678
424.0469	1	2190224.25
426.0441	1	2118229.25
827.1029	1	3394894
829.1002	1	2891291

Compound24

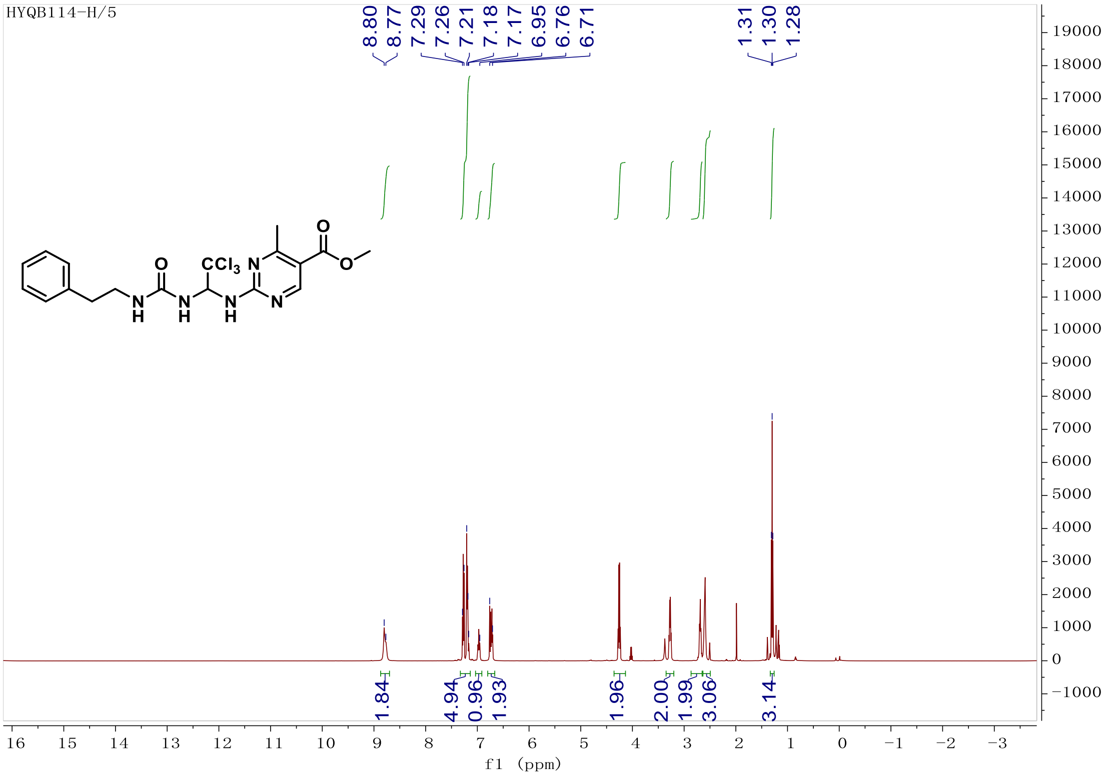

HYQB114-H/5
8.80
8.77
7.29
7.26
7.21
7.18
7.17
6.95
6.76
6.71
1.31
1.30
1.28
1.84
4.94
0.96
1.93
1.96
2.00
1.99
3.06
3.14
CCl3
O
N
H
f1 (ppm)

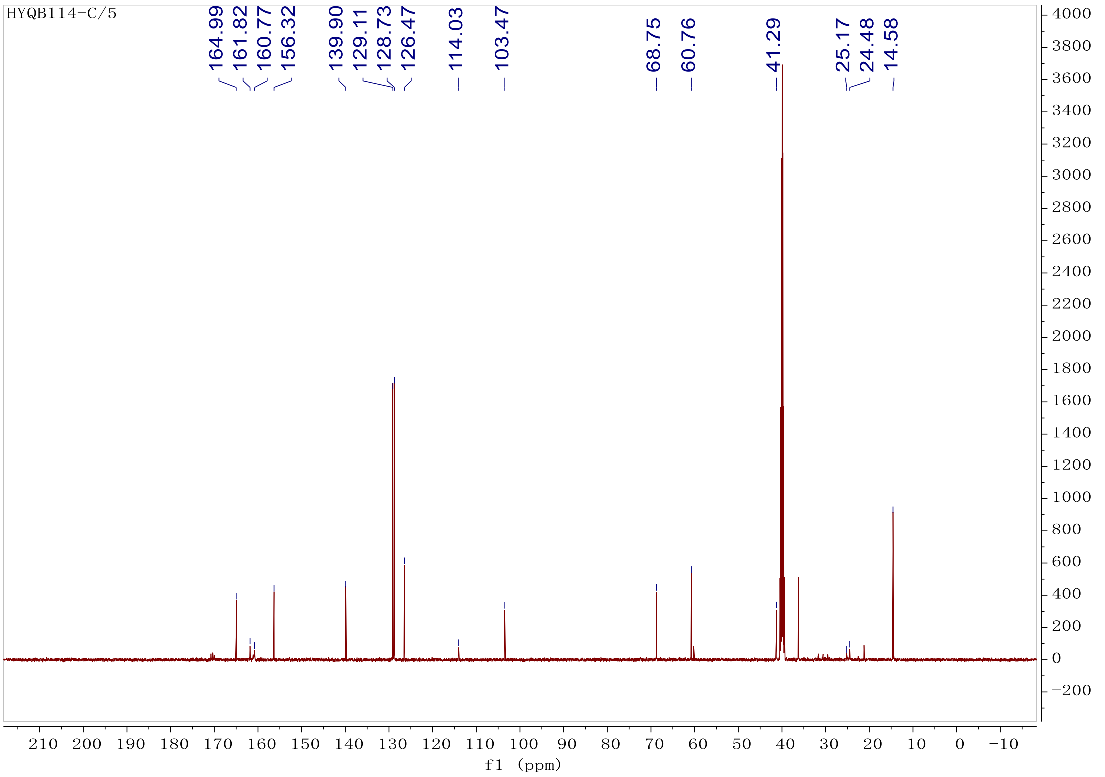

HYQB114-C/5
164.99
161.82
160.77
156.32
139.90
129.11
128.73
126.47
114.03
103.47
68.75
60.76
41.29
25.17
24.48
14.58
f1 (ppm)

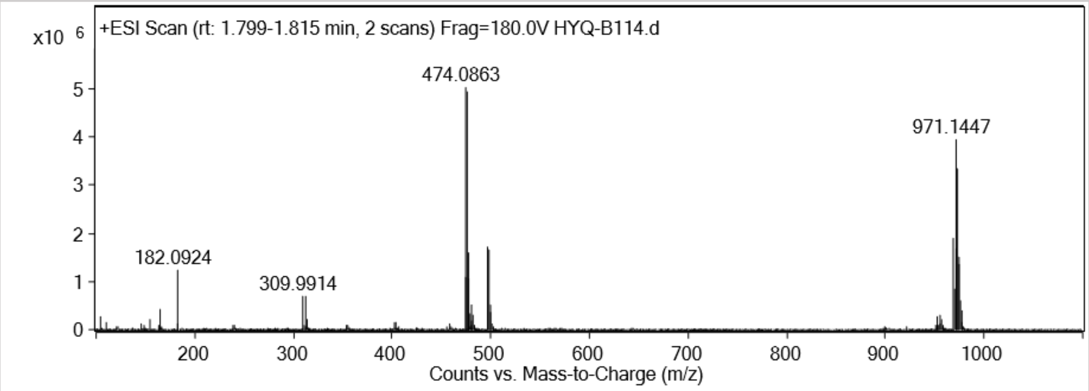

Peak List

m/z	z	Abund
474.0863	1	5031508
476.0836	1	4927891
478.0811	1	1595754.25
496.0683	1	1714726.5
498.0656	1	1669565.75
969.1469	1	1910078.5
971.1447	1	3947774
972.1475	1	1686540.25
973.1424	1	3334149.25
975.1403	1	1520962.25

Compound 25

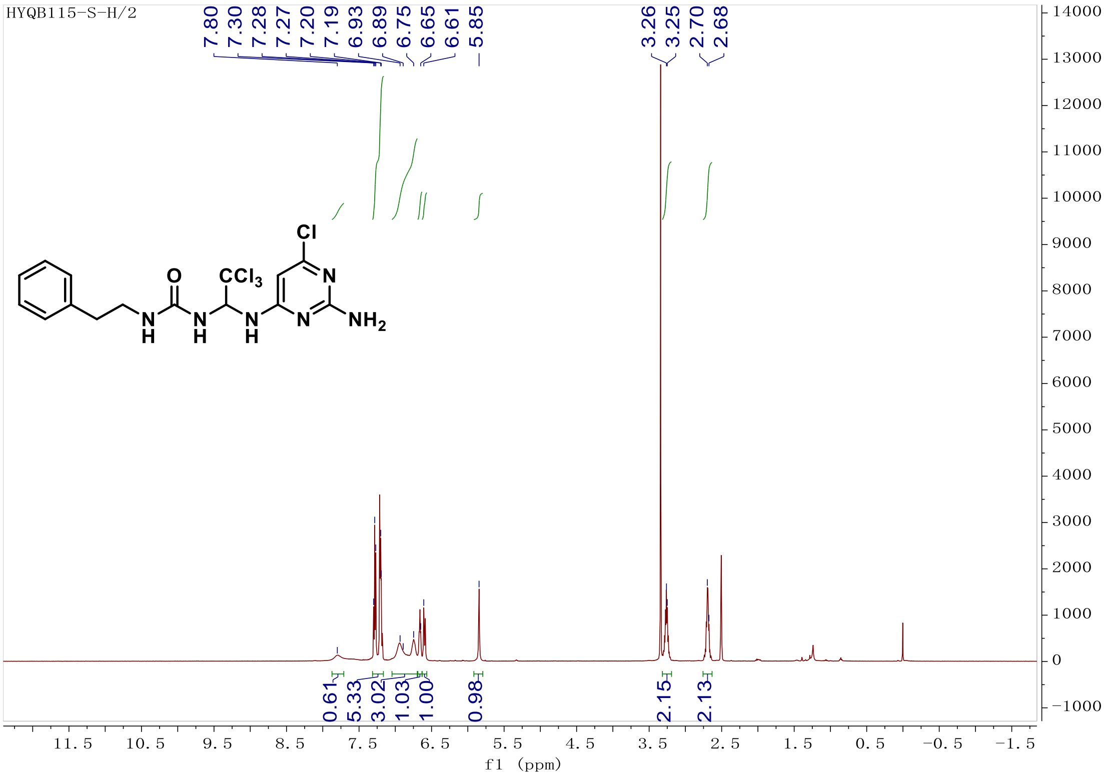

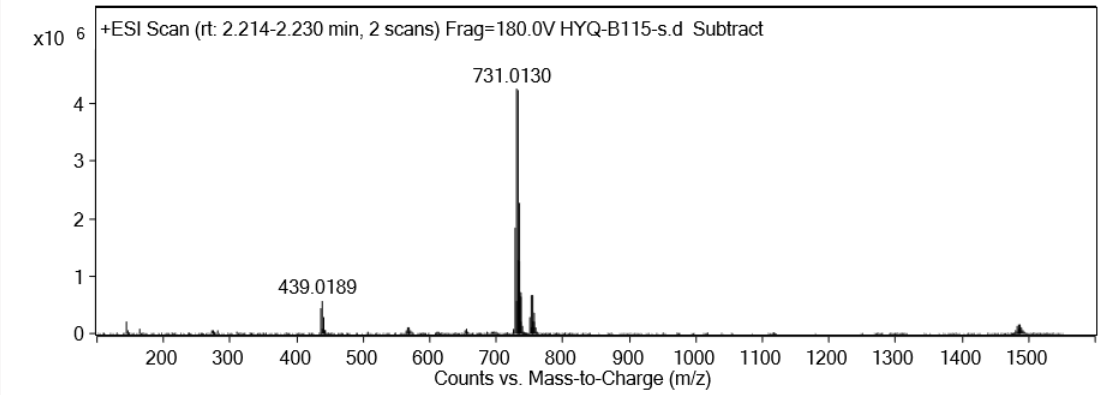

Peak List

m/z	z	Abund
729.0162	1	1839294.75
731.013	1	4260150.5
732.0158	1	1281294.25
733.0103	1	4241561.5
734.013	1	1251993.5
735.0077	1	2261063.25
736.0103	1	649382.75
737.0056	1	727508.88
752.9949	1	659895.44
754.9923	1	674211.88

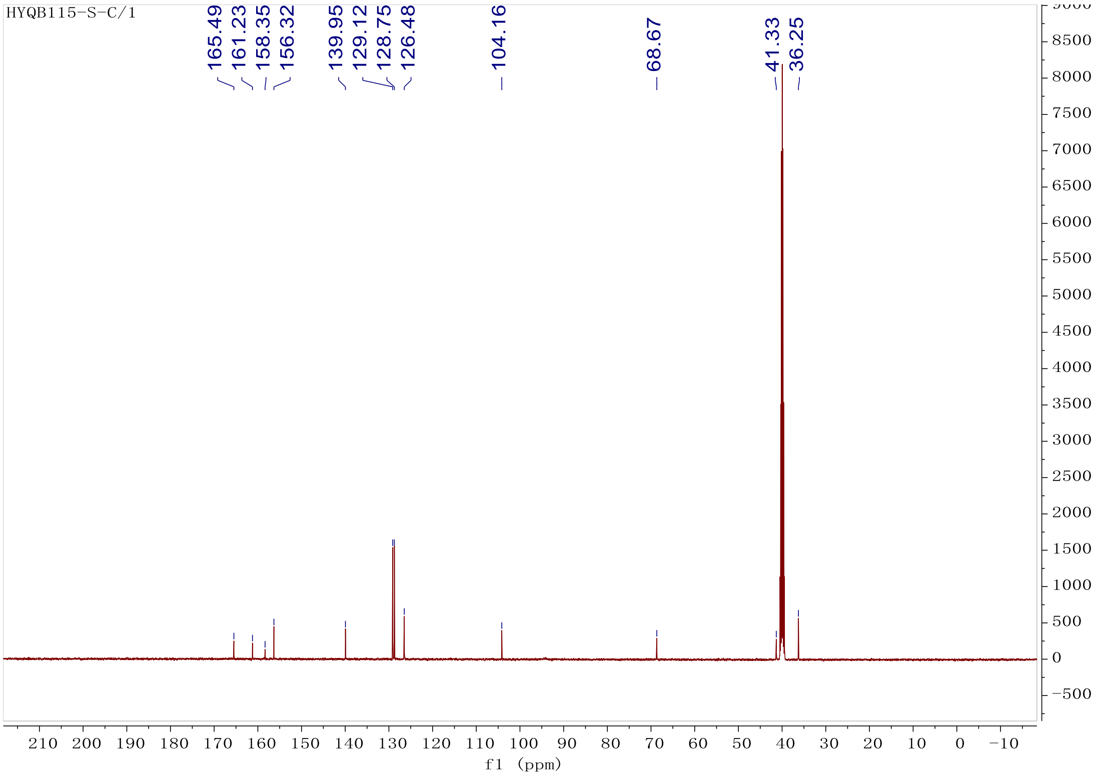

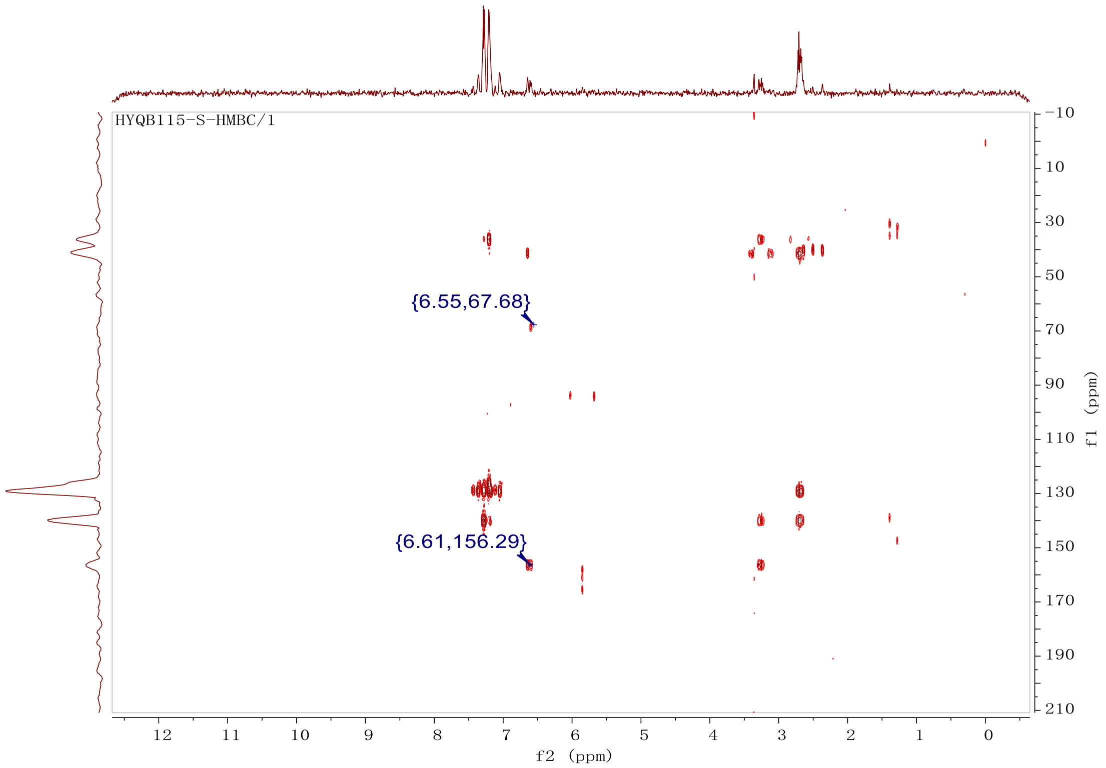
HYQB115-S-HMBC/1
{6.55,67.68}
{6.61,156.29}
f2 (ppm)
f1 (ppm)

Compund 26

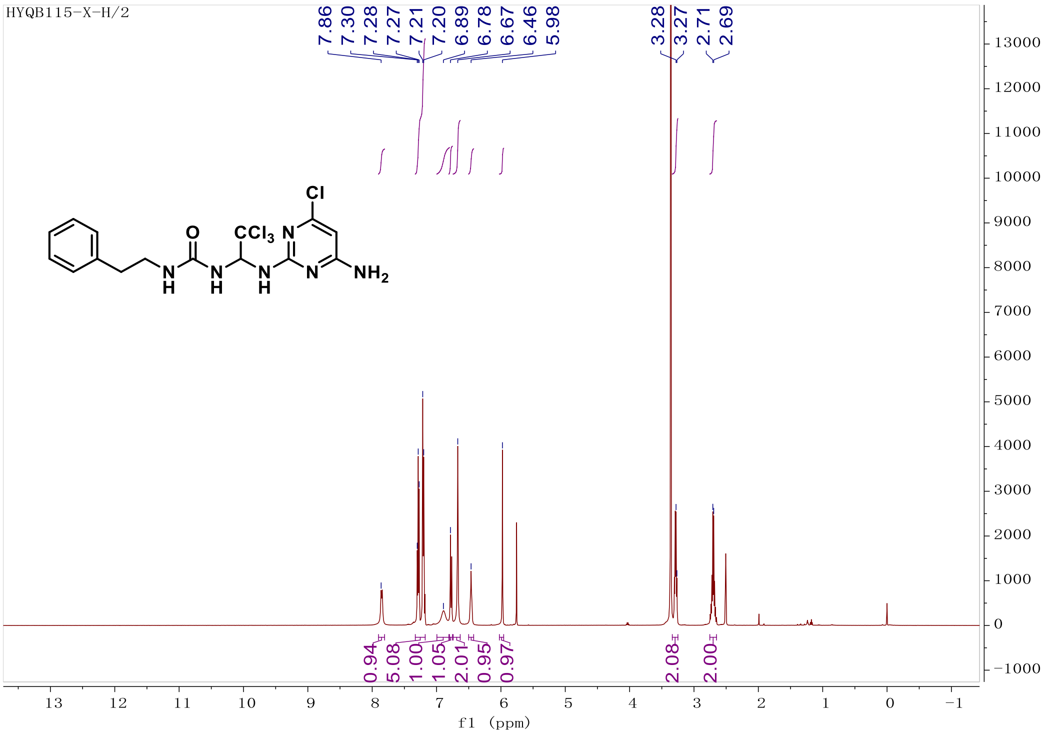
HYQB115-X-H/2
7.86
7.30
7.28
7.27
7.21
7.20
6.89
6.78
6.67
6.46
5.98
3.28
3.27
2.71
2.69
0.94
5.08
1.00
1.05
2.01
0.95
0.97
2.08
2.00
f1 (ppm)

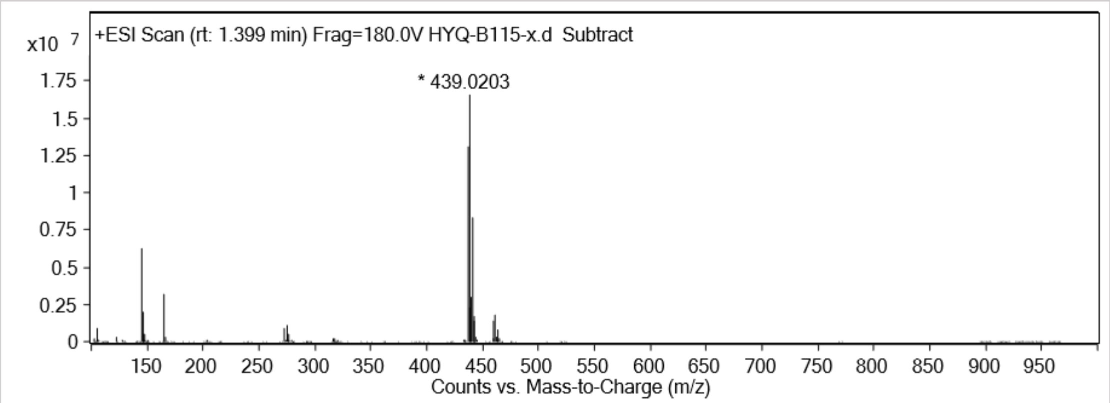

Peak List

m/z	z	Abund
145.0277	1	6224761
165.1024	1	3181428.25
437.0226	1	13036265
439.0203	1	16558550
440.0221	1	2990025.25
441.0166	1	8315304.5

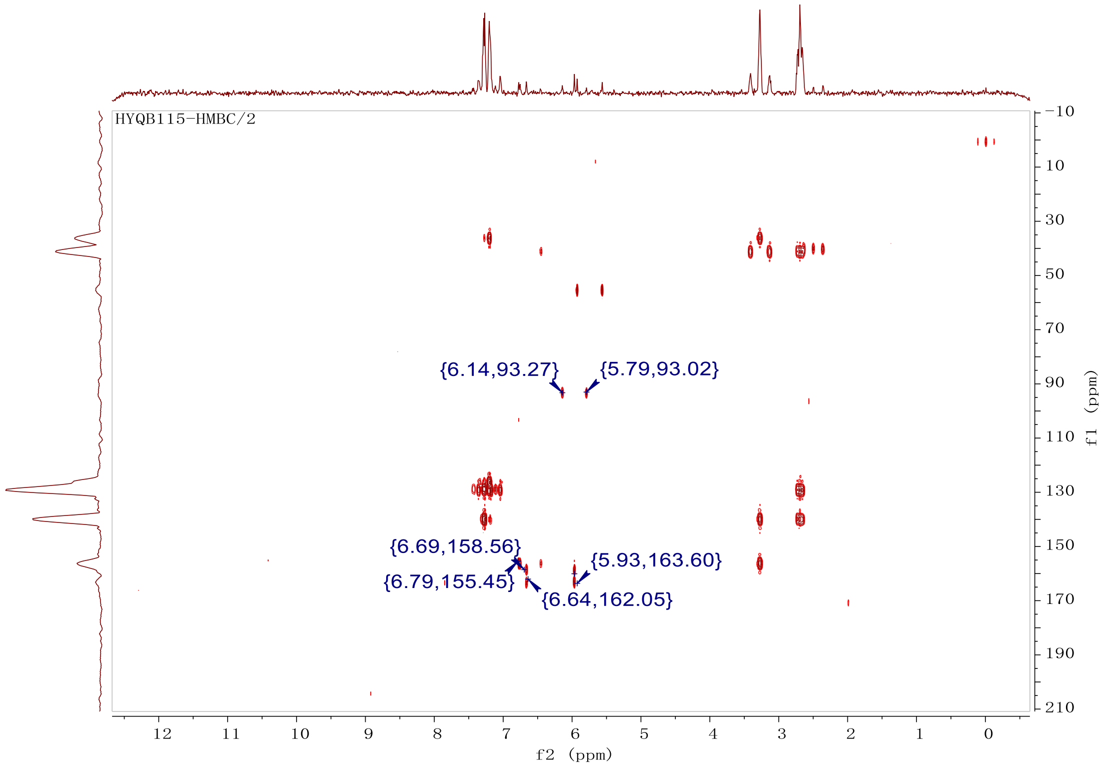

Compound 27

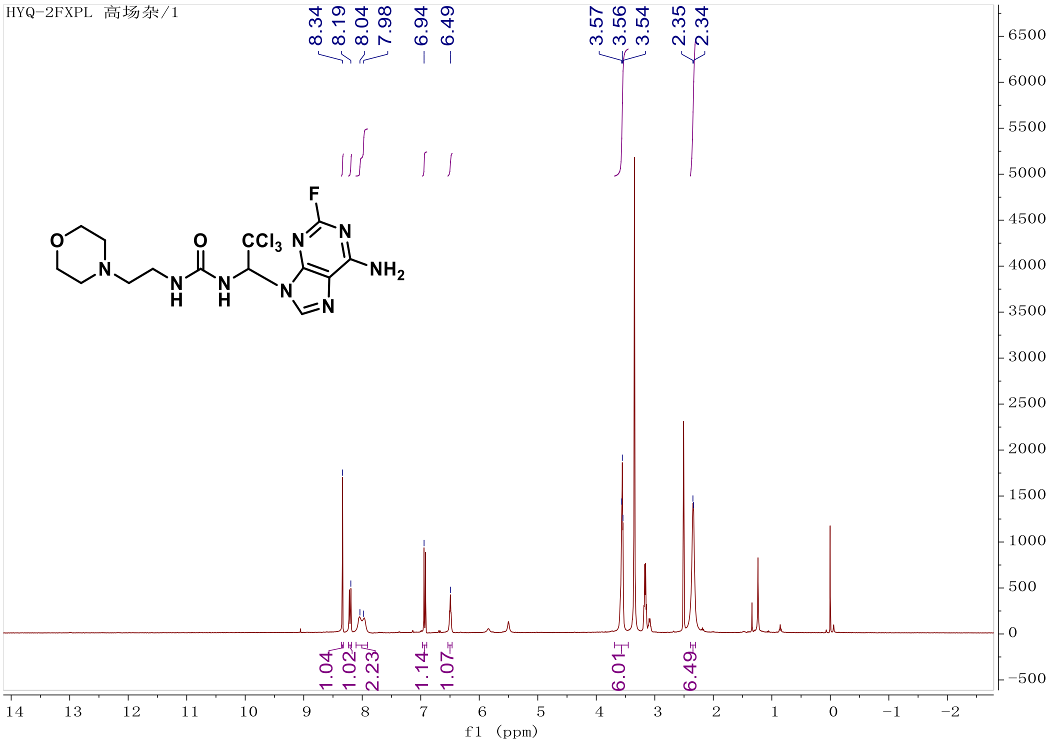
HYQ-2FXPL 高场杂/1
8.34
8.19
8.04
7.98
6.94
6.49
3.57
3.56
3.54
2.35
2.34
1.04
1.02
2.23
1.14
1.07
6.01
6.49
F
N
N
CCl3
O
O
N
N
H
N
H
N
N
NH2
f1 (ppm)

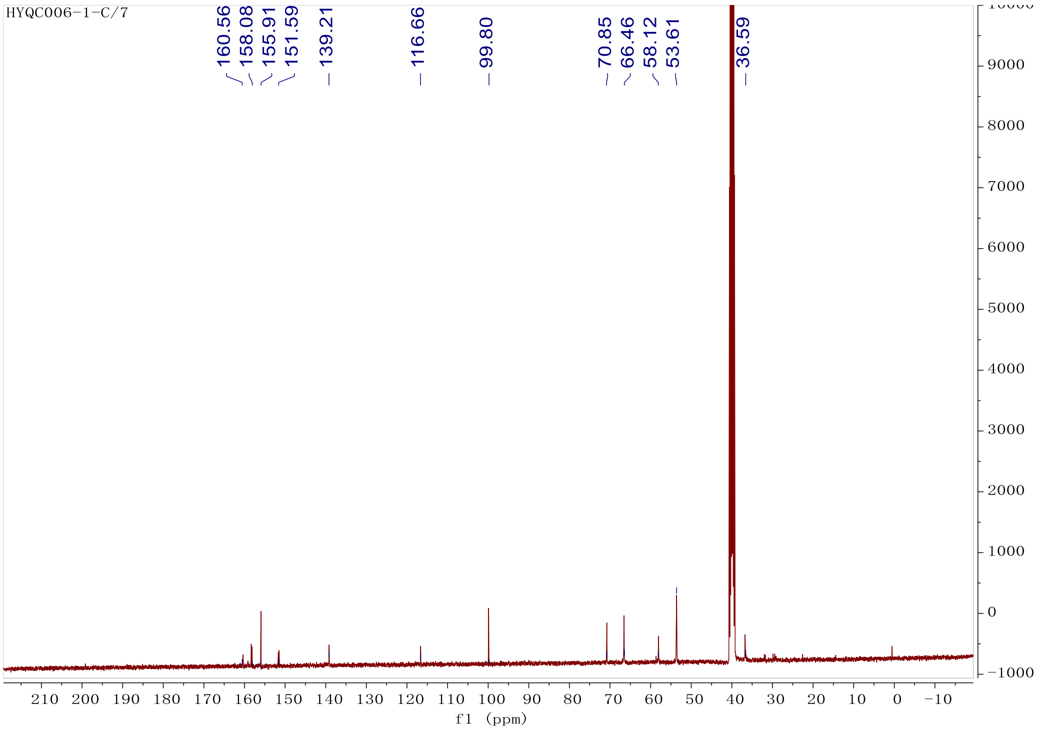
HYQC006-1-C/7
160.56
158.08
155.91
151.59
139.21
116.66
99.80
70.85
66.46
58.12
53.61
36.59
f1 (ppm)

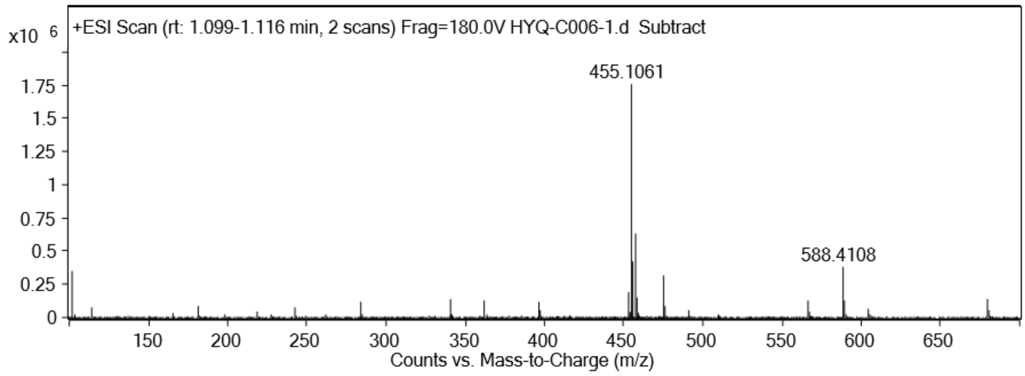

Peak List

m/z	z	Abund
102.128	1	343051.34
453.3445	1	190114.03
455.1061	1	1757479.13
456.1092	1	424070.75
457.1041	1	629228.38
475.3263	1	319590.78
588.4108	1	377484.72
701.4951	1	378013.75

Compound 28

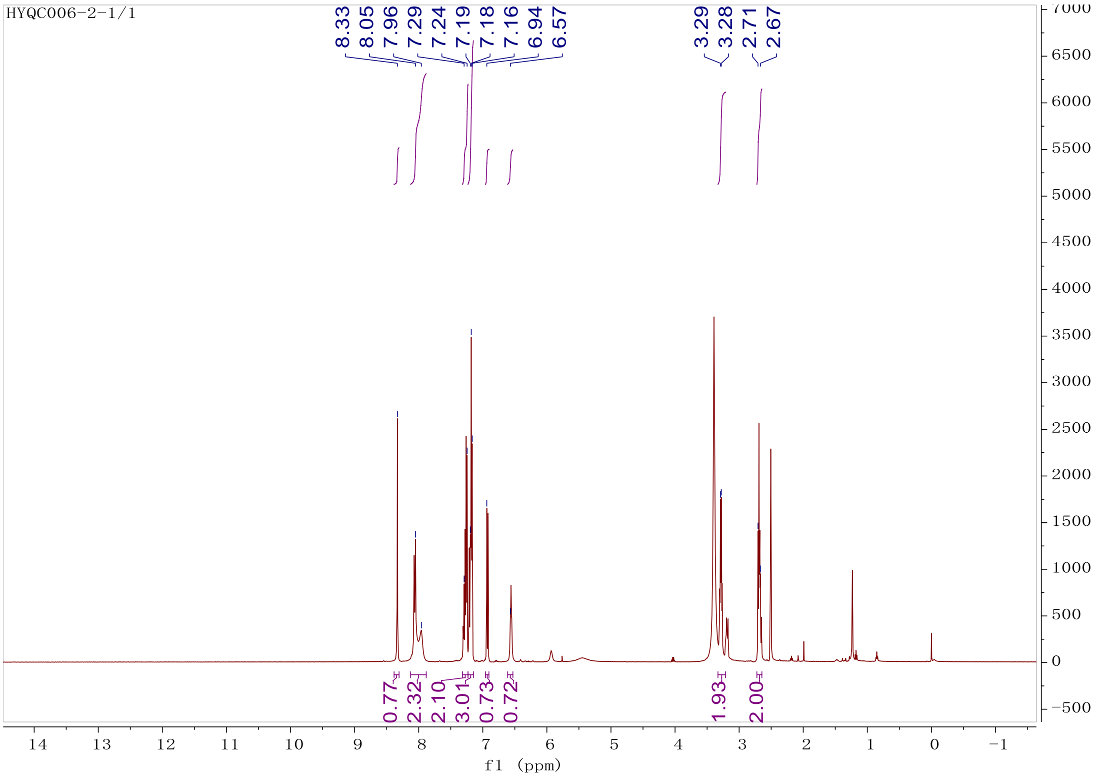

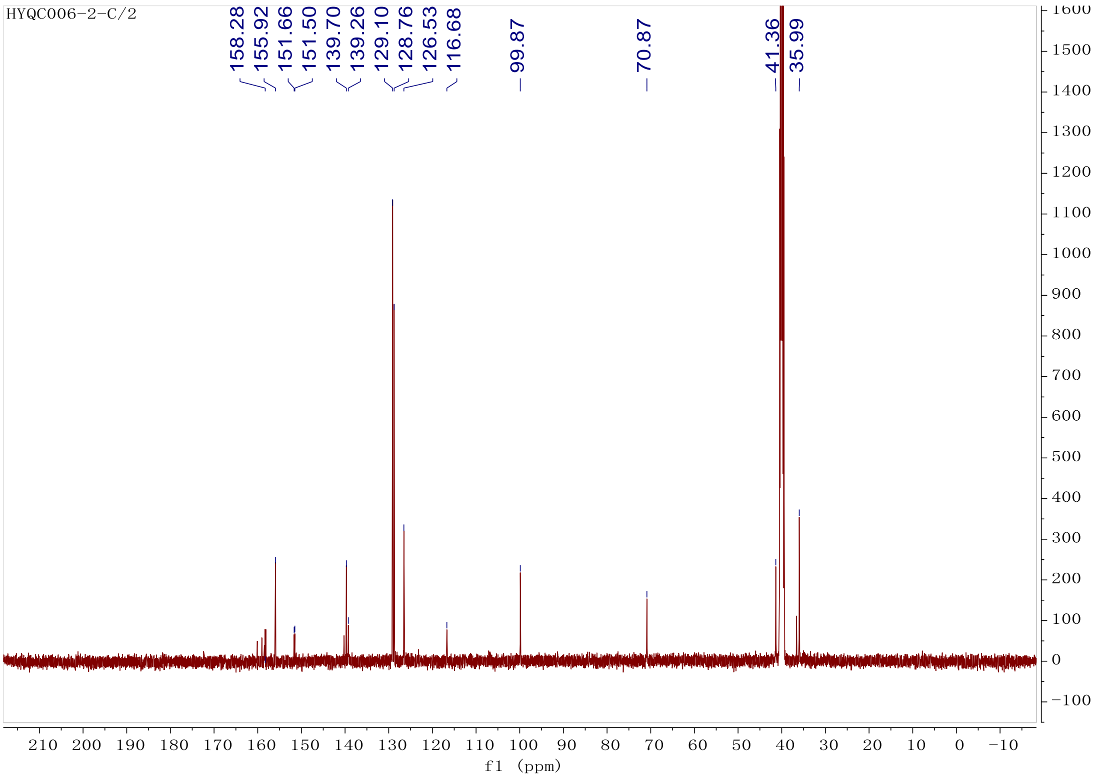

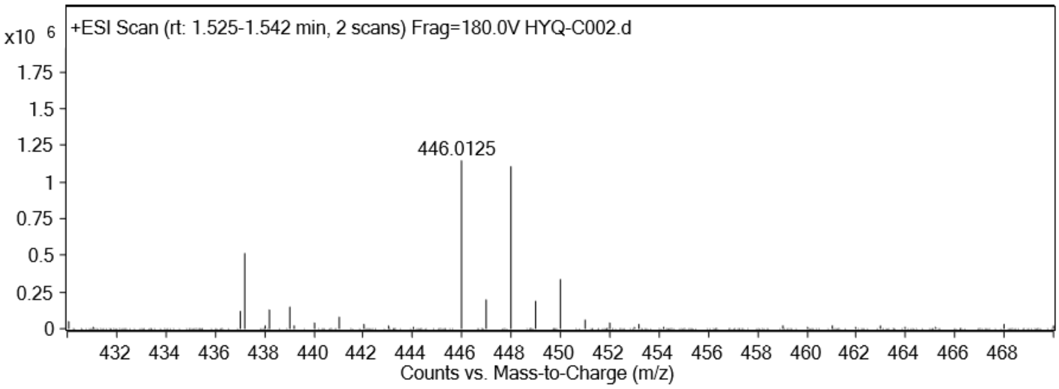

Peak List

m/z	z	Abund
110.0712	1	1342267.25
237.9701	1	912757.88
239.9672	1	842811.38
347.0093	1	811730.19
402.065	1	1648292.38
404.0622	1	1641297
424.0468	1	1364902.75
426.044	1	1314010.13
446.0125	1	1147873.13
448.0097	1	1103846.75

Compound 29

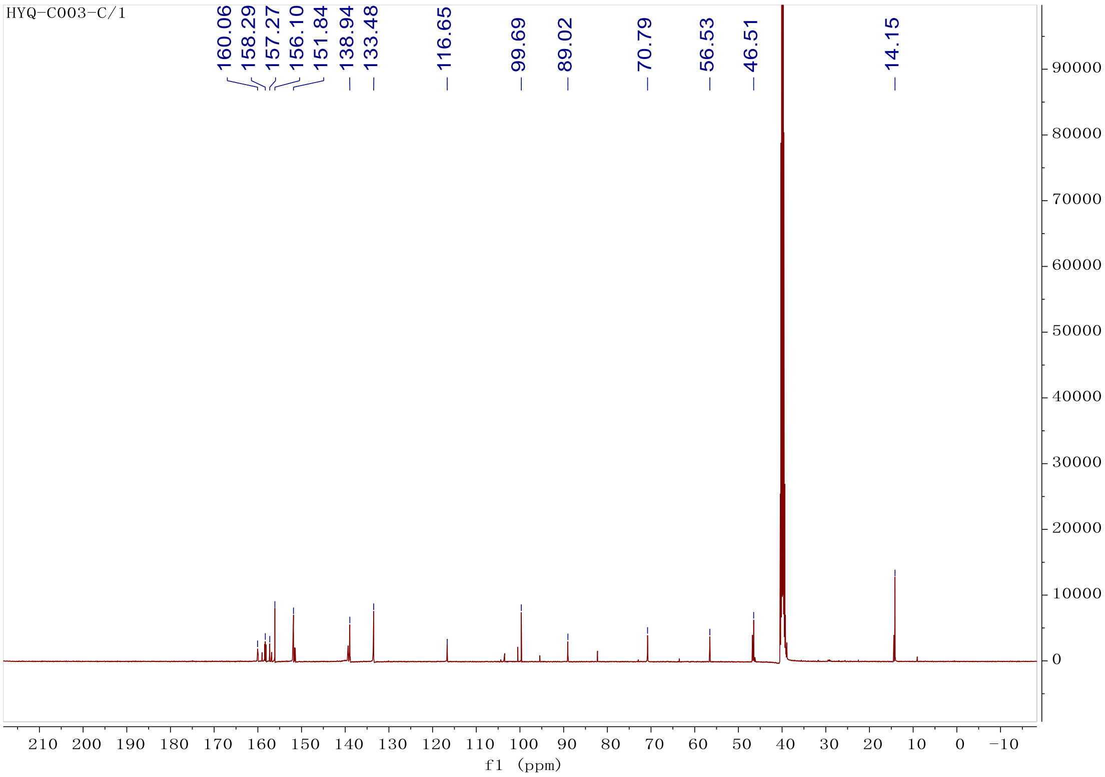
HYQ-C003-C/1
160.06
158.29
157.27
156.10
151.84
138.94
133.48
116.65
99.69
89.02
70.79
56.53
46.51
14.15
f1 (ppm)

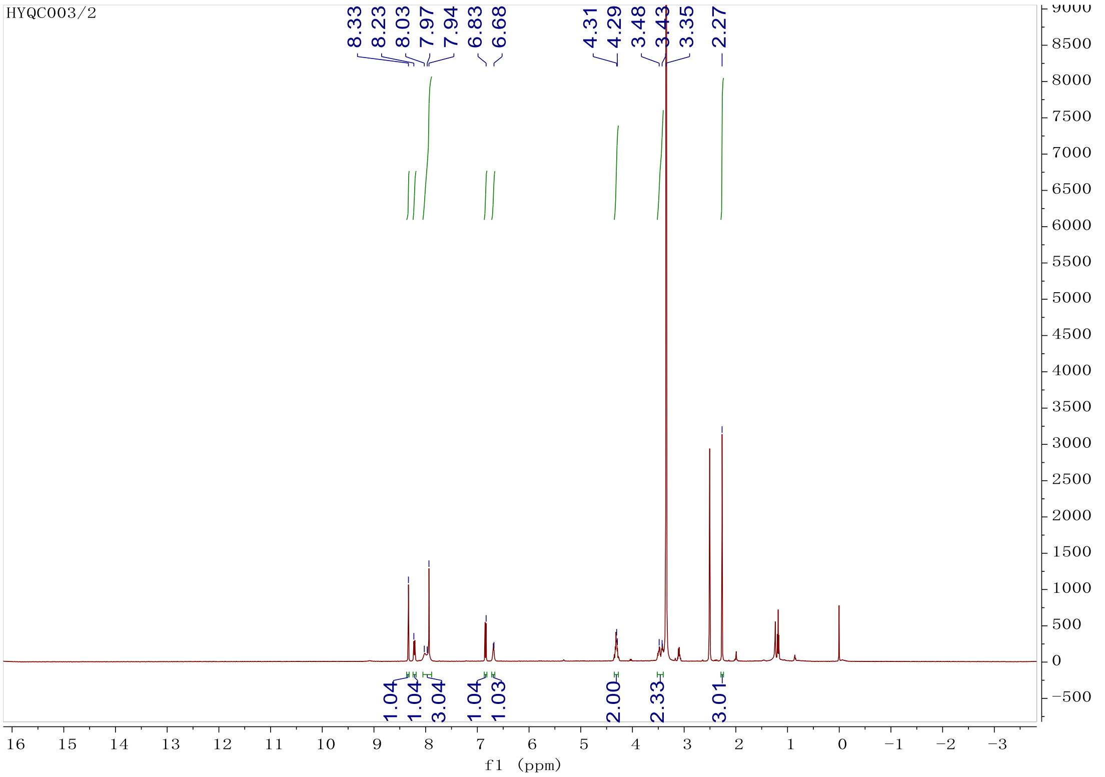
HYQC003/2
8.33
8.23
8.03
7.97
7.94
6.83
6.68
4.31
4.29
3.48
3.43
3.35
2.27
1.04
1.04
3.04
1.04
1.03
2.00
2.33
3.01
f1 (ppm)

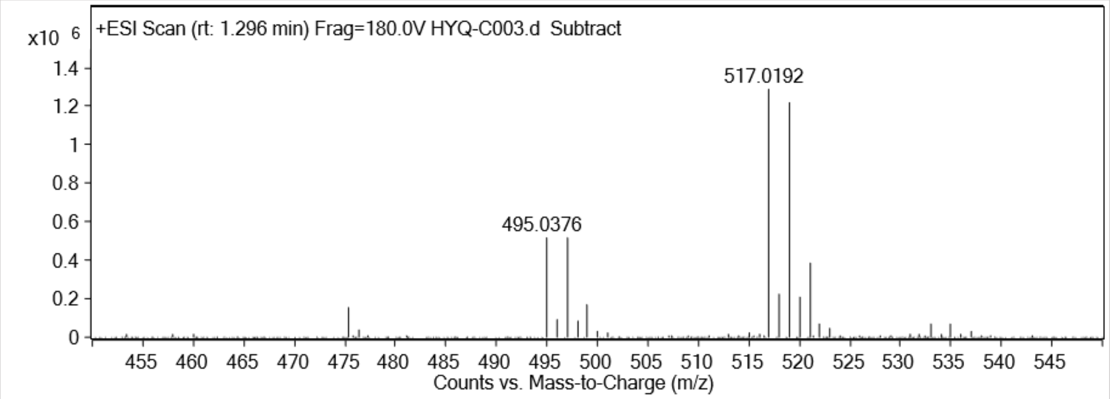

Peak List

m/z	z	Abund
154.0523	1	1044147.88
246.9804	1	1928471.25
248.9774	1	1832825.75
250.9748	1	538648.38
341.9924	1	1035881.13
343.9896	1	1010645.06
374.0187	1	1452116.75
376.0158	1	1405670
517.0192	1	1290025.25
519.0166	1	1223145.38

Compound 30

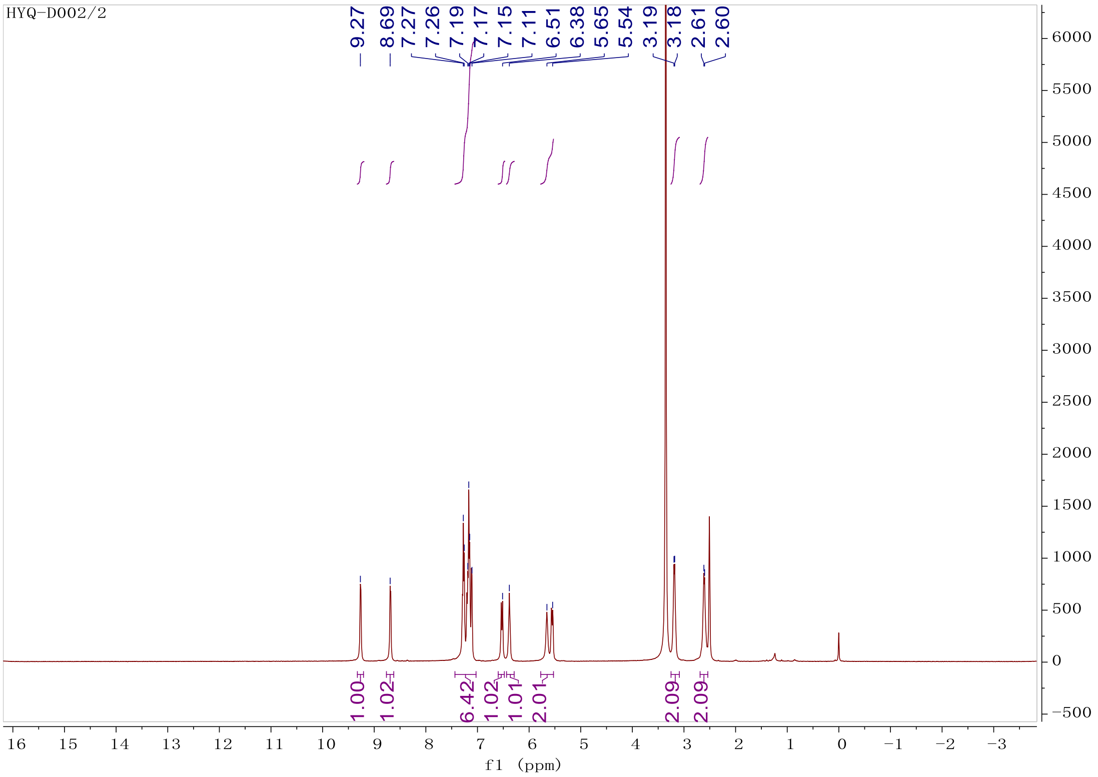

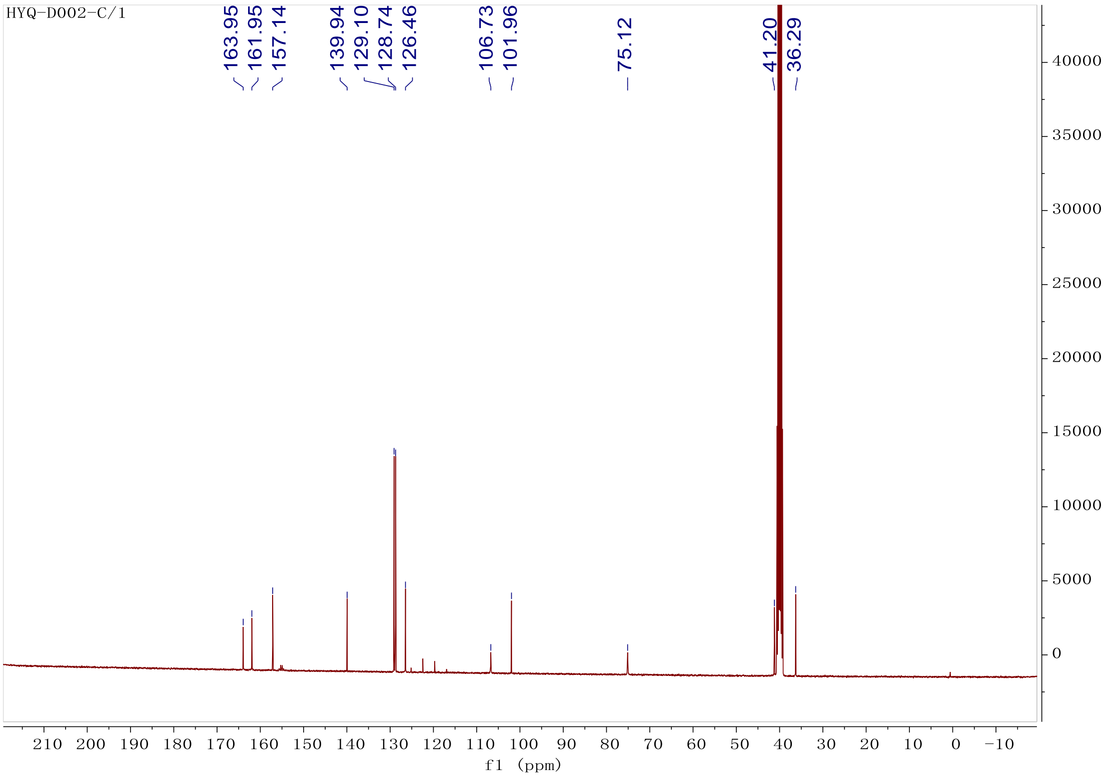

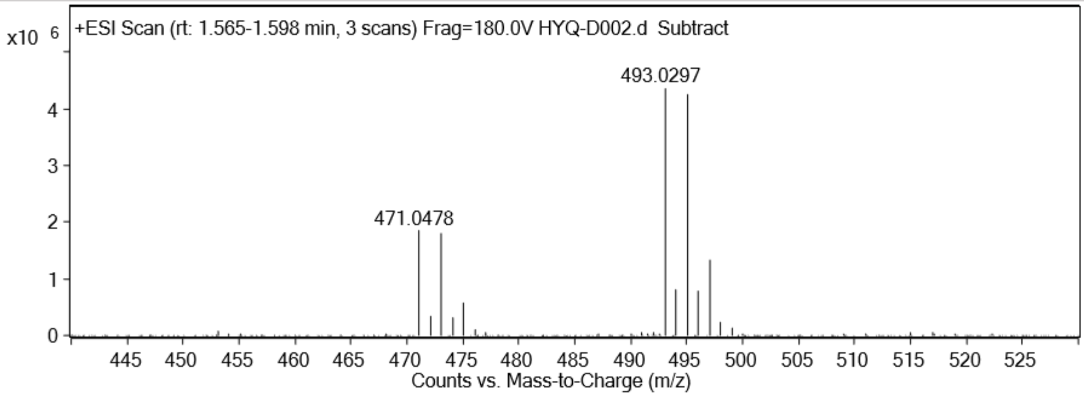

Peak List

m/z	z	Abund
165.1022	1	2057486.63
179.0539	1	2366741.75
409.0957	1	1080793.38
471.0478	1	1852333.88
473.045	1	1798070.88
493.0297	1	4356592.5
494.0325	1	820580.25
495.027	1	4253897
496.0299	1	778167.38
497.0245	1	1321068.63
